# Pleiotropy of genetic variants on obesity and smoking phenotypes: Results from the Oncoarray Project of The International Lung Cancer Consortium

**DOI:** 10.1371/journal.pone.0185660

**Published:** 2017-09-28

**Authors:** Tao Wang, Jee-Young Moon, Yiqun Wu, Christopher I. Amos, Rayjean J. Hung, Adonina Tardon, Angeline Andrew, Chu Chen, David C. Christiani, Demetrios Albanes, Erik H. F. M. van der Heijden, Eric Duell, Gadi Rennert, Gary Goodman, Geoffrey Liu, James D. Mckay, Jian-Min Yuan, John K. Field, Jonas Manjer, Kjell Grankvist, Lambertus A. Kiemeney, Loic Le Marchand, M. Dawn Teare, Matthew B. Schabath, Mattias Johansson, Melinda C. Aldrich, Michael Davies, Mikael Johansson, Ming-Sound Tsao, Neil Caporaso, Philip Lazarus, Stephen Lam, Stig E. Bojesen, Susanne Arnold, Xifeng Wu, Xuchen Zong, Yun-Chul Hong, Gloria Y. F. Ho

**Affiliations:** 1 Department of Epidemiology & Population Health, Albert Einstein College of Medicine, Bronx, New York, United States of America; 2 Department of Epidemiology & Biostatistics, School of public health, Peking University Health Science Center, Beijing, China; 3 Community and Family Medicine, Geisel School of Medicine, Dartmouth College, Hanover, New Hampshire, United States of America; 4 Lunenfeld-Tanenbaum Research Institute, Sinai Health System; Division of Epidemiology, Dalla Lana School of Public Health, University of Toronto, Toronto, Ontario, Canada; 5 IUOPA. University of Oviedo and CIBERESP. Oviedo, Spain; 6 Norris Cotton Cancer Center, Hanover, New Hampshire, United States of America; 7 Fred Hutchinson Cancer Research Center, Seattle, Washington, United States of America; 8 Harvard School of Public Health, Boston, Massachusetts, United States of America; 9 National Cancer Institute, Bethesda, United States of America; 10 Radboud university medical center, Nijmegen, Netherlands; 11 Catalan Institute of Oncology (ICO), Barcelona, Spain; 12 Carmel Medical Center, Haifa, Israel; 13 International Agency for Research on Cancer (IARC), Lyon, France; 14 University of Pittsburgh Cancer Institute, Pittsburgh, Pennsylvania, United States of America; 15 Roy Castle Lung Cancer Research Programme, Department of Molecular & Clinical Cancer Medicine, The University of Liverpool, Liverpool, UK; 16 Department of surgery, Unit for breast surgery, Lund University, Malmö, Skåne University Hospital Malmö, Malmö, Sweden; 17 Department of Medical Biosciences, Umeå University, Umeå, Sweden; 18 University of Hawaii Cancer Center, Honolulu, Hawai'I, United States of America; 19 University Of Sheffield, Sheffield, South Yorkshire, United Kingdom; 20 Department of Cancer Epidemiology, H. Lee Moffitt Cancer Center and Research Institute, Tampa, Florida, United States of America; 21 Department of Thoracic Surgery, Division of Epidemiology, Vanderbilt University Medical Center, Nashville, Tennessee, United States of America; 22 Princess Margaret Cancer Center, Toronto, Ontario, Canada; 23 Washington State University College of Pharmacy, Washington, United States of America; 24 British Columbia Cancer Agency, Vancouver, British Columbia, Canada; 25 Copenhagen General Population Study, Herlev and Gentofte Hospital, Copenhagen, Denmark; 26 Department of Clinical Biochemistry, Herlev and Gentofte Hospital, Copenhagen University Hospital, Copenhagen, Denmark; 27 Faculty of Health and Medical Sciences, University of Copenhagen, Copenhagen, Denmark; 28 Markey Cancer Center, Lexington, Kentucky, United States of America; 29 The University of Texas MD Anderson Cancer Center, Texas, Houston, United States of America; 30 Department of Preventive Medicine, Seoul National University College of Medicine, Seoul, Korea; 31 Merinoff Center for Patient-Oriented Research, The Feinstein Institute for Medical Research, New York, United States of America; 32 Epidemiology and Research, Northwell Health, New York, United States of America; 33 Hofstra Northwell School of Medicine, New York, United States of America; McMaster University, CANADA

## Abstract

Obesity and cigarette smoking are correlated through complex relationships. Common genetic causes may contribute to these correlations. In this study, we selected 241 loci potentially associated with body mass index (BMI) based on the Genetic Investigation of ANthropometric Traits (GIANT) consortium data and calculated a BMI genetic risk score (BMI-GRS) for 17,037 individuals of European descent from the Oncoarray Project of the International Lung Cancer Consortium (ILCCO). Smokers had a significantly higher BMI-GRS than never-smokers (p = 0.016 and 0.010 before and after adjustment for BMI, respectively). The BMI-GRS was also positively correlated with pack-years of smoking (p<0.001) in smokers. Based on causal network inference analyses, seven and five of 241 SNPs were classified to pleiotropic models for BMI/smoking status and BMI/pack-years, respectively. Among them, three and four SNPs associated with smoking status and pack-years (p<0.05), respectively, were followed up in the ever-smoking data of the Tobacco, Alcohol and Genetics (TAG) consortium. Among these seven candidate SNPs, one SNP (rs11030104, *BDNF*) achieved statistical significance after Bonferroni correction for multiple testing, and three suggestive SNPs (rs13021737, *TMEM18*; rs11583200, *ELAVL4*; and rs6990042, *SGCZ*) achieved a nominal statistical significance. Our results suggest that there is a common genetic component between BMI and smoking, and pleiotropy analysis can be useful to identify novel genetic loci of complex phenotypes.

## Introduction

Both obesity and cigarette smoking are risk factors for many human diseases, including multiple cancers.[1–4] There are complex sources of correlations between smoking behavior and obesity.[5,6] In general, current smokers tend to have a lower body mass index (BMI) than never-smokers, while smoking cessation is associated with weight gain.[7–9] The reasons for the association between BMI and smoking status may involve smoking-induced appetite suppression via neural pathways [10] and increased energy expenditure via energy-regulating hormonal feedback loops.[11,12] On the other hand, heavy smokers tend to have a greater BMI than light smokers; an observation that is seemingly contradictory to the metabolic effects of smoking,[7,13] but may be partially attributed to the unhealthy behaviors associated with heavy smoking. Another reason for the correlation between smoking behavior and obesity is that there may be common underlying biological causes. There is growing evidence suggesting that obesity may be partially due to addiction to food.[14,15] One plausible common mechanism for obesity and smoking is brain reward effects arising from neuronal activity within the dopamine system.[16] In any case, the reasons for the relationship between BMI and smoking behavior remain uncertain.

Shared genetic susceptibility may offer another explanation for the correlation between obesity and smoking. Both smoking and obesity have significant genetic components. In the past, large-scale genome-wide association studies (GWAS) on obesity or variables related to smoking characteristics (e.g., smoking status, age started smoking, and pack-years of smoking, etc) have successfully identified multiple loci associated with these phenotypes.[17–25] Yet, total variation in obesity or smoking traits explained by these GWAS loci is still limited.[20,26–29] The remaining genetic variants still need to be identified. It was estimated that the genetic correlation between smoking status and BMI was 0.20.[30] In a previous study in Iceland, the genetic risk score (GRS) of 32 common variants identified in GWAS of BMI was associated with smoking initiation and the number of cigarettes smoked per day (CPD), suggesting that smoking and BMI may share common genetic components.[31] However, this study in Iceland only observed correlations of BMI associated SNPs with smoking variables, without accounting for possible causal relationships between SNPs, BMI and smoking variables. We hypothesized that analyzing pleiotropic effects on BMI and smoking behavior may discover novel genetic loci, otherwise undiscovered in GWAS with stringent genome-wide significance, which in turn would further elucidate genetic architectures underlying both smoking behavior and obesity. In this study, leveraging existing genotyping, BMI, and smoking data from a lung cancer consortium, we confirmed the association between the BMI-GRS and smoking-related variables with adjustment for BMI and important covariates, and used causal network inference to identify potential genetic loci with pleiotropic effects on both BMI and smoking-related phenotypes.

## Materials and methods

### Study population

The International Lung Cancer Consortium (ILCCO) was established in 2004 with the goal of sharing comparable research data and maximizing research efficiency (http://ilcco.iarc.fr). To further characterize cancer genetic architecture of common cancers, a custom OncoArray (http://oncoarray.dartmouth.edu) genotyping chip that includes 550K markers was designed to genotype samples in collaboration with other cancer consortia under The National Cancer Institute (NCI) initiative on the Genetic Associations and Mechanisms in Oncology (GAME-ON). In this study, we analyzed OncoArray genotypic data of 36,000 subjects of European descent in ILCCO; among them, 17,037 provided individual epidemiological data and were of European descent.

### OncoArray genotyping, quality control and imputation

The GAME-ON OncoArray chip was previously described.[32] In brief, it includes a GWAS backbone and a customized panel for dense mapping of known susceptibility regions, rare variants from sequencing experiments, pharmacogenetic markers and cancer related traits including smoking and BMI. The genotyping quality control of Oncoarray data was previously described.[33] After filtering out SNPs by success rate and genotype distribution deviation from the expected by Hardy-Weinberg equilibrium, 517,482 SNPs were available for analysis. Standard quality control procedures were used to exclude underperforming samples (2,408), unexpected duplicated or related samples (2,411), samples with sex error (316) and non-Caucasians (8,240). After quality control, 17,037 subjects with full information on both BMI and smoking status, and other important covariates (age, sex, study sites, and lung cancer status) were kept for analysis. Genotype data were imputed by the GAME-ON data coordinating center for all scans for over 10 million SNPs using data from the 1000 Genomes Project (Phase 3, October 2014) as reference.[34,35]. The data were imputed in a two-stage procedure using SHAPEIT [36] to derive phased genotypes, and IMPUTEv2 to perform imputation of the phased data. [35] Genotypes were aligned to the positive strand in both imputation and actual genotyping.

### SNP selection and derivation of the BMI-GRS

We first identified a large set of 4,961 SNPs associated with BMI with p<10^−5^ based on results from the Genetic Investigation of ANthropometric Traits (GIANT) consortium, a large collaborative GWAS on human body size and shape. We then pruned SNPs by applying a threshold value of r^2^ = 0.2 and requiring selected SNPs at least 500Kb apart to reduce redundancy and obtained a subset of 241 independent SNPs that were at least 500Kb apart (S1 Table). To calculate the BMI-GRS, each SNP was recoded as 0, 1, or 2 according to the number of risk alleles (BMI increasing alleles). The BMI-GRS was calculated using the equation: GRS = (weight1×SNP1 + weight 2×SNP2 + … + weight n×SNPn), where n is the total number of SNPs. Both un-weighted and weighted BMI-GRSs were calculated in which the weight is 1 for all SNPs and the weight is the β coefficient of each individual SNP on BMI derived from GIANT for the un-weighted and weighted GRS, respectively. The results of un-weighted and weighted GRS were largely similar and we presented un-weighted BMI-GRS in the Results. To examine the robustness of the results based on the BMI-GRS of our selected SNPs, we also calculated the BMI-GRS based on 97 BMI-associated SNPs which reached genome-wide significant levels (P< 5×10–8) in the GIANT BMI GWAS including up to 322,154 European descents and 17,072 non-European descents [37].

### Statistical analysis

Age, sex, smoking statuses, pack-years, BMI, and BMI categories were compared between lung cancer statuses by student t-test and Chi-square test for categorical variables. All statistical tests are two-sided. The analyses were performed using R (v2.6).

### Association of BMI with smoking phenotypes

Linear regression model was applied for comparison of BMI among individuals with different smoking categories (never-smokers, current smokers and ex-smokers) with adjustment for age, sex, and study sites. Adjusted means of BMI of individuals with different smoking categories and their 95% confidence intervals (CIs) were calculated using the *lm* function in the statistical software R with a fixed intercept of zero. Additional stratification analyses by lung cancer status were performed.

### Association of the BMI-GRS with BMI and smoking phenotypes

Linear regression was also used to compare the BMI-GRS between different BMI categories (underweight, <18.5; Normal, 18.5–24.9; Overweight, 25.0–29.9; and Obese, ≥ 30) by adjusting for age, sex, study sites, and top four genetic principal components. Although our analyses were performed only for participants of European descent, study sites and top four genetic principle components generated using common SNPs were included in the regression models for BMI-GRS in order to further limit the effects of any possible cryptic population stratification that might cause inflation of test statistics. Trend tests were performed by analyzing the BMI categories as a continuous variable in the regression model. A similar regression analysis was also performed to compare the BMI-GRS between individuals with different smoking categories (never-smokers, current-smokers, and ex-smokers). Partial correlation coefficients between the BMI-GRS and pack-years of smoking were estimated by Pearson correlation coefficients of their residuals from linear regression models after adjusting for age, sex, study sites, and top four genetic principal components. Additional stratification analyses were performed by lung cancer status.

### Identifying candidate pleiotropic SNPs for BMI and smoking phenotypes

**Fig 1:**
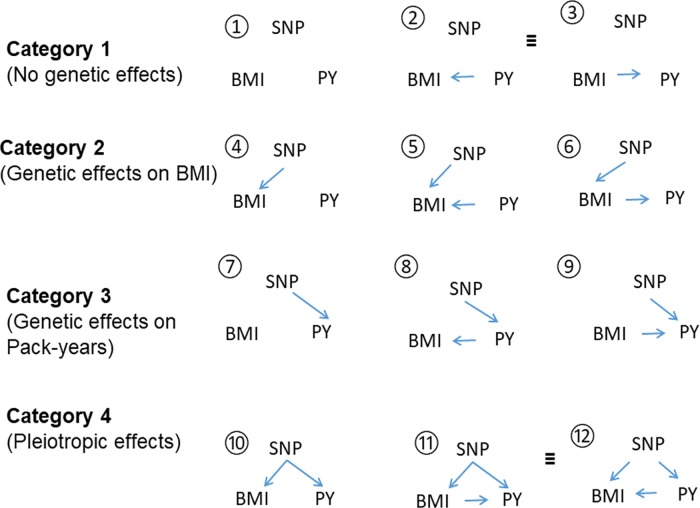
Twelve possbile directed acyclic graphs (DAGs) of one SNP, BMI and pack-years (PY) of smoking. Possible DAGs between one SNP, BMI and PY. The DAGs are categorized into 4 groups. SNPs in Category 1 (DAGs of 1, 2, and 3) do not have effects on either BMI or pack-years. SNPs in Category 2 (DAGs of 4, 5, and 6) have direct effects on BMI, but not PY. SNPs in Category 3 (DAGs of 7, 8, and 9) have direct effects on PY, but not BMI. SNPs in Category 4 (DAGs of 10, 11, and 12) have pleiotropic effects on BMI and PY. ≡ represents models that are not differentiable.

We used a causal network inference model to identify possible pleiotropic SNPs for both BMI/pack-years and BMI/smoking status (smokers versus non-smokers), respectively.[38] We describe the approach here using BMI and pack-years as an example. A similar approach was used to identify pleiotropic SNPs for BMI and remaining smoking traits. Specifically, we modeled 12 possible directed acyclic graphs (DAGs) of the genotype value of a SNP on BMI and/or pack-years (**Fig 1**). We classified these DAGs into four categories: (1) the SNP did not have effects on either BMI or pack-years, (2) the SNP had direct effects on BMI, but not pack-years, (3) the SNP had direct effects on pack-years, but not BMI, and (4) the SNP had pleiotropic effects on both BMI and pack-years.

Based on a given DAG, we fit two linear regression models for BMI and pack-years, respectively, with adjustment for sex, age, study sites, and genetic principal components (PCs). For example, two linear regression models for the DAG of SNP → pack-years → BMI (DAG 8 with gentic effects on pack-years only) are

BMI∼Age+Sex+StudySites+PCs+Packyears,

Packyears∼Age+Sex+StudySites+PCs+SNP.

To identify the model that was the most supported by the data, we calculated AIC for each DAG

-2loglik(RegressionModel1)–2loglik(RegressionModel2)+2*numberofedges.

We then compared the minimum AIC values of four categories. SNPs with at least 2 of the minimum AIC value of category 4 (model 10, 11, or 12) less than other categories were further examined for their association with pack-years. Similar analyses were performed for smoking status using logistic regression. Those SNPs that achieved a nominal statistical significance (p<0.05) were considered as candidate pleiotropic SNPs, and further validated for their associations with ever-smoking using the independent database from TAG (The Tobacco, Alcohol and Genetics) consortium.(https://www.med.unc.edu/pgc/results-and-downloads)

## Results

### Characteristics of the study population

In our analysis, 17,037 subjects of European descent from 17 study sites had full information on both BMI and smoking status, and other important covariates (age, sex, study sites, and lung cancer status). As expected, compared to the controls, lung cancer cases were older, had a higher proportion of smokers, and were slightly leaner (**Table 1)**.

**Table 1:** Characteristics of 17,037 European-descent subjects in the OncoArray Project and epidemiologic data.

	Cases	Controls	P-values
N	9,633	7,404	
Male (%)	5,461 (56.7)	42,71 (57.7)	0.199
Age (sd)	65.2 (10.2)	61.1 (10.1)	<0.001
Smoking type (%)			
Never-smokers	1,101 (11.4)	2,353 (31.8)	<0.001
Ex-smokers	3,934 (40.8)	2,782 (37.6)	
Current smokers	4,598 (47.7)	2,269 (30.6)	
Pack-years of smoking among smokers (sd)	47.8 (31.3)	33.1 (26.5)	<0.001
BMI, kg/m^2^ (sd)	26.3 (4.9)	26.9 (4.8)	<0.001
BMI categories (kg/m^2^)			<0.001
Under weight (<18.5)	268 (2.8)	66 (0.9)	
Normal (18.5–24.9)	3,862 (40.1)	2,708 (36.6)	
Over weight (25–29.9)	3,684 (38.2)	3,154 (42.6)	
Obese (≥30)	1,819 (18.9)	1,476 (19.9)	

The Basic characteristics of the subjects were described as mean (sd) for continuous variables, and number (proportion, %) for category variables. The p-values were obtained by student t-test for continuous variables and Çhi-square test for category variables.

### Association of BMI and smoking variables

**Fig 2:**
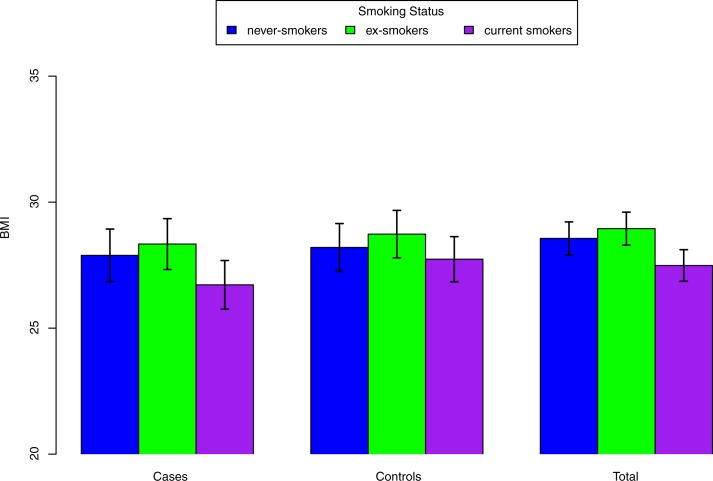
Adjusted means of BMI (95% CIs) for never-smokers, ex-smokers, and current smokers with adjustment for age, sex, and study sites. Bar represents mean±s.d.

We compared BMI levels among never-smokers, ex-smokers, and current smokers. As expected and as compared to never-smokers, ex-smokers had a significantly higher BMI (difference from never-smokers = 0.39 kg/m^2^, p = 2.64×10^−4^), while current smokers were leaner (difference from never-smokers = -1.08 kg/m^2^, p = 8.20×10^−24^) after adjustment for age, sex and study sites. Such differences in BMI by smoking status were similar for cases and controls (**Fig 2**).

BMI and pack-years of smoking were positively correlated in both current smokers and ex-smokers after adjustment for age, sex and study sites, and the correlations were stronger in ex-smokers than those in current-smokers (**Table 2**). Specifically, the partial coefficient of pack-years of smoking and BMI was 0.054 (95%CI 0.027–0.075) and 0.112 (95%CI 0.088–0.136) for current smokers and ex-smokers, respectively. The correlations between BMI and pack-years were similar for cases and controls.

**Table 2:** Partial correlations between BMI and pack-years of smoking by smoking status.

	Current Smokers				Ex-smokers			
	n	Coef	95%CI	P	N	Coef	95%CI	P
All*	6,577	0.054	0.027–0.075	<0.001	6,245	0.112	0.088–0.136	<0.001
Cases**	4,396	0.052	0.023–0.082	<0.001	3,682	0.106	0.074–0.138	<0.001
Controls**	2,181	0.072	0.030–0.114	<0.001	2,563	0.140	0.102–0.178	<0.001

A table for the partial correlation coefficients between BMI and pack-years in smokers.

* For all subjects, the analysis was adjusted for age, sex, study sites and disease status.

** For cases and controls, the analyses were adjusted for age, sex, and study sites.

### Association of the BMI-GRS with BMI

**Fig 3:**
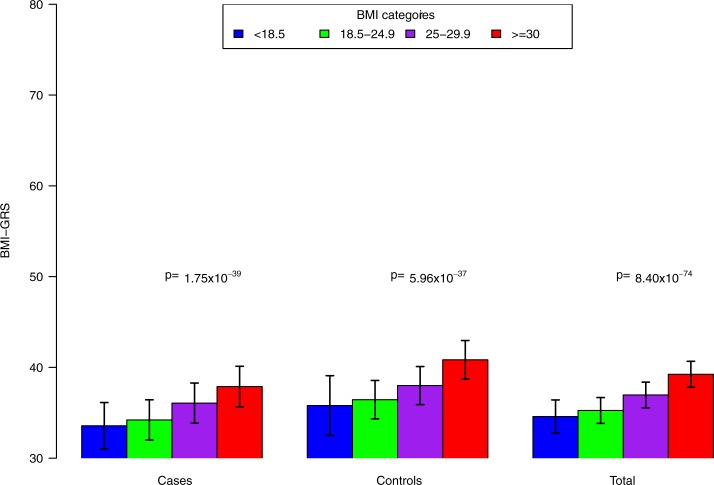
Adjusted means of the BMI-GRS (95% CIs) by BMI category after adjustment for age, sex, study sites, genetic principal components, smoking status, and pack-years of smoking. Bar represents mean±s.d.

We first confirmed if the BMI-GRS based on 241 SNPs identified in GIANT was associated with BMI in the OncoArray Project population. Comparing the BMI-GRS of individuals in different BMI categories with adjustment for age, sex, study sites, genetic principal components, smoking types, and pack-years (**Fig 3**), we found that the BMI-GRS significantly increased from the categories underweight (BMI<18.5), to normal weight (BMI 18.5–24.9), to overweight (BMI 25–29.9), and to obese (BMI ≥30) with p_trend_ = 8.40×10^−74^. Similar associations between the BMI-GRS and BMI categories were found in cases and controls (p_trend_ = 1.75×10^−39^ and p _trend_ = 5.96×10^−37^ for cases and controls, respectively).

### Association of the BMI-GRS with smoking phenotypes

The BMI-GRS of smokers that include both ex-smokers and current smokers, was first compared with that of never-smokers. Smokers had a significantly higher BMI-GRS than never-smokers (regression coefficient of ex-smokers: 0.516, p-value = 0.016) before adjustment for BMI. After further adjustment for BMI, the association between the BMI-GRS and smoking was strengthened (regression coefficient 0.545, p-value = 0.010). Ex-smokers and current smokers were then compared separately with that of never-smokers. Ex-smokers and current smokers had a similar BMI-GRS (regression coefficient 0.059, p-value = 0.750), but both of them had a significantly higher BMI-GRS than never-smokers (regression coefficient of ex-smokers: 0.490, p-value = 0.032; regression coefficient of current smokers: 0.549, p-value = 0.021) before adjustment for BMI. After further adjustment for BMI, the association between the BMI-GRS and current smoking was strengthened (regression coefficient 0.838, p-value = 3.9×10^−4^), while the association between the BMI-GRS and ex-smoking was somewhat attenuated (regression coefficient 0.321, p-value = 0.157). The association patterns based on different BMI-GRSs were largely consistent (S2 Table). The results were also similar when analyses were stratified by lung cancer status.

There was also a significantly positive association between the BMI-GRS and pack-years of smoking among smokers (**Table 3**). The associations were similar in cases and controls. After stratification by smoking status, the association between the BMI-GRS and pack-years tended to be stronger in current smokers (correlation coefficient 0.024, p = 0.049) than in ex-smokers (correlation coefficient 0.009, p = 0.472). The results based on different BMI-GRSs were largely consistent (S3 Table).

**Table 3:** Partial correlations between pack-years of smoking and BMI-GRS.

Category*	Coef	95%CI	p-value
Total (n = 12,822)**	0.022	0.004–0.039	0.014
Stratified by smoking categories**			
Current smokers (n = 6,575)	0.024	0.0001–0.048	0.049
Ex-smokers (n = 6,245)	0.009	-0.016–0.034	0.472
Stratified by disease status***			
Cases (n = 8,078)	0.018	-0.004–0.040	0.109
Controls (n = 4,744)	0.031	0.003–0.060	0.030

*The partial correlation coefficients between BMI-GRS and pack-years were calculated in smokers.

** The correlation coefficients were adjusted for age, sex, BMI, study sites, genetic principal components, and disease status.

*** The correlation coefficients were adjusted for age, sex, BMI, study sites, and genetic principal components.

### Identifying pleiotropic SNPs for both BMI and smoking status

**Fig 4:**
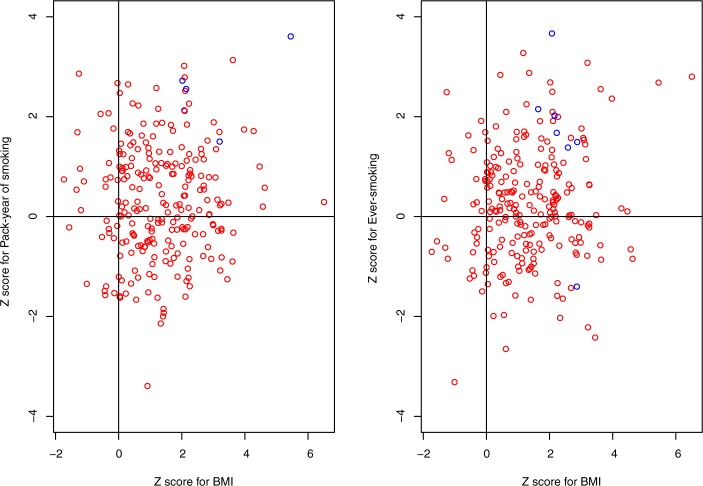
The Z statistics for associations of 241 SNPs with BMI, pack-years of smoking, and smoking. The vertical axis represents Z scores for associations of SNPs with pack-years of smoking or smoking. The horizontal axis represents Z scores for associations of SNPs with BMI. All Z scores were adjusted by age, sex, study sites, genetic principal components and lung cancer disease status. The blue dots were SNPs that were determined to be pleiotropic with further validation in TAG.

The above analyses suggested that among the 241 SNPs composed of the BMI-GRS (or in linkage disequilibrium with the 241 SNPs), there may be some pleiotropic SNPs that have direct effects on smoking and BMI. To identify the pleiotropic loci (DAGs 10, 11, or 12 in Fig 1), we first used network inference to determine the possible causal models of 241 SNPs. In total, five SNPs were classfied into category four to be pleiotropic for both BMI and pack-years of smoking, and seven SNPs were classfied into category four to be pleiotropic for both BMI and smoking by network inference. We then examined the associations of each of these SNPs with BMI and pack-years of smoking (or smoking status) with adjustment for age, sex, study sites, genetic principal components and lung cancer disease status. (**Fig 4**). There were four and three SNPs associated with pack-years of smoking and smoking status with p<0.05, respectively (**Table 4**). The SNPs classified as Category 4 and associated with smoking status or pack-years with a nominal significance (p<0.05) were considered as candidate SNPs of pleiotropy. The associations of these candidate pleiotropic SNPs with BMI were quite similar with and without adjustment for pack-years or smoking status (S4 Table). We validated these SNPs using data from the TAG consortium. Of the total of seven candidate pleiotropic SNPs for BMI and pack-years or smoking status, rs11030104 (BDNF) was associated with ever-smoking in TAG data after Bonferroni correction (p = 0.002), and rs13021737 (TMEM18), rs11583200 (ELAVL4) and rs6990042 (GCZ) achieved a nominal significance of 0.05 (p-values were 0.018, 0.008, and 0.043, respectively). Another interesting SNP that achieved a nominal significance in TAG data (p = 0.020) was rs12016871 (MTIF3), but it did not achieve statistical significance with smoking status in the OncoArray Project dataset (p = 0.161).

**Table 4:** SNPs with pleiotropic effects on BMI and smoking varibles among 241 SNPs composed of the BMI-GRS.

SNP	chr	position	Gene	OncoArray Project				GIANT		TAG	
				Association direction with smoking phenotype	p-value of association with smoking variable	Association direction withBMI	p-value association with BMI	Association Direction with BMI	p-value association with BMI	Association direction with ever-smoking	p-value association with ever- smoking
**SNPs pleiotropic for pack-years and BMI**											
rs13021737	2	632348	TMEM18	Positive	3.0e-4	Positive	5.0E-08	Positive	5.4e-54	Positive	0.018
rs1528435	2	181550962	AC009478.1	Positive	6.4e-3	Positive	0.043	Positive	4.8e-09	Negative	0.936
rs11583200	1	50559820	ELAVL4	Positive	0.011	Positive	0.036	Positive	6.0e-09	Positive	0.008
rs3888190	16	28889486	ATP2A1	Positive	0.034	Positive	0.038	Positive	3.5e-25	Negative	0.606
rs11165643	1	96924097	PTBP2	Positive	0.133	Positive	0.001	Positive	1.4e-13	Positive	0.282
**SNPs pleiotropic for smoking status and BMI**											
rs11030104	11	27684517	BDNF	Positive	2.4e-4	Positive	0.038	Positive	6.7e-30	Positive	2.0e-4
rs6990042	8	14173974	SGCZ	Positive	0.031	Positive	0.101	Positive	4.5e-07	Positive	0.045
rs9275595	6	32681355	XXbac-BPG254F23.7	Positive	0.043	Positive	0.031	Positive	5.6e-06	Positive	0.214
rs7550711	1	110082886	GPR61	Positive	0.094	Positive	0.026	Positive	5.1e-14	Positive	0.594
rs929641	2	58792377	LINC01122	Positive	0.136	Positive	0.004	Positive	5.1e-08	Positive	0.761
rs12016871	13	28017782	MTIF3	Negative	0.161	Positive	0.004	Positive	9.3e-11	Negative	0.020
rs12220375	10	104901491	NT5C2	Positive	0.168	Positive	0.010	Positive	1.8e-09	Positive	0.142

The SNPs presented in this table were classified as Categoriy four (DAGs of 10,11,12 in Fig 1). The association directions and p-values of these SNPs with BMI in GIANT and with ever-smoking in TAG are also presented. SNPs in the shadow were statistically signficant for the association with pack-years of smoking or smoking status in the OncoArray Project population (p<0.05) and with ever-smoking data of TAG consortium with a nominal significance of p<0.05.

## Discussion

In summary, we calculated the BMI-GRS for subjects who had OncoArray data of ILCCO using 241 common SNPs potentially associated with BMI and demonstrated that the BMI-GRS was associated with increased propensity to smoke as well as elevated pack-years after adjusting for the potential confounding effects of BMI. These results were consistent with those from a previous study in Iceland in which the GRS of 32 SNPs identified in GWAS was found to be associated with two smoking phenotypes, smoking initiation and the number of cigarettes smoked per day. The observed associations between the BMI-GRS and smoking variables could not be due to confounding of BMI, because the association of the BMI-GRS with smoking varaibles remained statistically significant after adjustment for BMI. Moverover, the BMI-GRS was positively associated with current smoking, which was opposite to what would be expected if the association between BMI-GRS and current smoking was due to the confounding effects of BMI, as current smoking and BMI was negatively correlated. Instead, the associations between BMI-GRS and smoking indicate that some loci that composed the BMI-GRS may directly contribute to smoking behavior, and may have pleiotropic effects on both BMI and smoking variables.

Using causal network inference, we identified 4 loci that may have pleitropic effects on BMI and pack-years of smoking and 3 loci with potential pleitropic effects on BMI and smoking status. Among them, one locus (BDNF) achieved a statistical significance after Bonferroni correction (p<0.007), and three loci (TMEM18, ELAVL4, and SGCZ) achieved a nominal significance (p<0.05). in ever-smoking data from the TAG consortium. The result of *BDNF* (brain derived neurotrophic factor) locus on chromosome 11 was consistent with prior studies that had shown strong associations of this locus with BMI [18,39] and various smoking phenotypes.[17] Evidence from epidemiological studies [40] and animal studies [41] also indicate associations of *BDNF* gene with other substance abuse related disorders, eating disorders, and schizophrenia. The protein *BDNF* belongs to a neurotrophin family growth factors [42] and is the most abundant of the neurotrophins in the brain with high concentrations in the hippocampus and cerebral cortex.[43,44] *BDNF* expression in the brain is regulated by the serotonergic[45] and the dopaminergic[46] neurotransmitter systems which are known to be involved in nicotine use, addictive behaviors, mood and food intake. [47–50]

The *TMEM18* (transmembrane protein 18) locus is another known GWAS locus of BMI.[19] The Icelandic study that examined 32 GWAS loci of BMI had found significant associations of *TMEM18* with both smoking initiation and cigarettes per day, an observation that was consistent with what we found [31]. The function of *TMEM18* is largely unknown. *TMEM18* is highly expressed in neural tissue and has been hypothesized to play a role in energy homeostasis via neural pathways controlling food intake [51].

**Fig 5:**
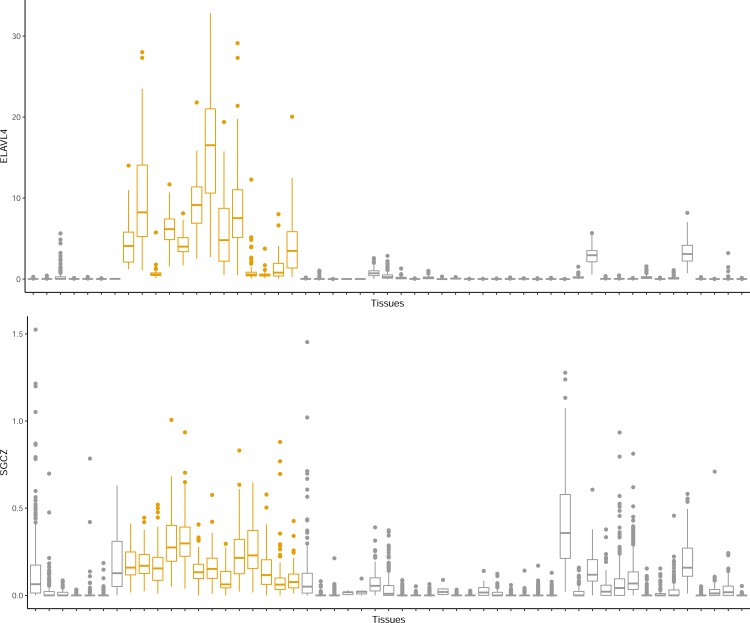
The gene expressions of ELAVL4 and SGCZ across different tissue in GTEx database. The bar shows the expression median and interalquartile range in different tissues. The yellow bars repsent tissues in brain.

To our knowledge, both *ELAVL4* (ELAV Like RNA Binding Protein 4) and *SCGZ* (Sarcoglycan zeta) loci have not been associated with smoking bahavior in GWAS. We examined the GTEx database, and both *ELAVL4* ad *SGCZ* are highly expressed in multiple brain tissues (**Fig 5**). The *ELAVL4* gene is known to be associated with hallucinogen abuse, paraneoplastic neurologic disorders, and Parkinson disease [52]. Although there was suggestive evidence of association between *SCGZ locus and BMI*, it has not been considered as the GWAS BMI locus;[20] however, a previous copy number variation (CNV) analysis in two African American populations had identified a CNV overlapping with *SGCZ* gene region to be signficantly associated with BMI.[53] Previously, *SGCZ* and other genes invovled in cell adhesion processes were linked to addiction vulnerability.[54] Cell adhesion mechanisms are central for properly establishing and regulating neuronal connections during development and can play major roles in mnemonic processes in adults [55–57]. In addition to reward processes, there are growing bodies of data implicating that cell-adhesion and related memory-like processes play important roles in substance dependence.[54,55,57,58]

Future studies of identification of pleiotropic genes on both BMI and smoking phenotypes may focus on pathways of those candidate loci, in particular *BDNF* gene. Among 241 SNPs, there was one SNP (rs3800229) in the locus of *FOXO3* that can be inactivated by signaling pathways acctived by neurotrophins (such as *BDNF*).[59,60] This SNP was assciated with pack-years in the OncoArray Project data and ever-smoking in TAG data with a nominal signficance of p<0.05 (data not shown), but this SNP did not achived the cut-off to be classified into the pleiotropic categoriy. Nevertheless, our finding on associatons of *BDNF* suggests the regulatory pathway of *BDNF* and its other target loci may play a role in both smoking behavior and BMI.

In general, genetic variants, BMI and smoking phenotypes are in complex relationships. In addtion to pleiotropic effects of genetic varaints on BMI and smoking phentoypes, there may also be interactions between genetic variants and smokings on BMI. For example, a recent study identified several novel BMI loci by accounting for SNP-smoking interactions. [61] In the presence of such interaction, one would also expect assoications between a SNP and smoking status in BMI-based ascertained samples, although the SNPs is not associated with smoking status in the general population. A future study fully accounting for these relationships may reveal additional novel loci of obesity and smoking phenotypes.

In summary, we identified four potential loci that may have pleiotropic effects on BMI and smoking traits. All four potential pleiotropic loci on BMI and smoking phenotypes are expressed in the human brain, and prior experimental evidence indicates that these genes are invovled in relevant complex brain functions, e.g. brain’s reward circutry and neural cell adhesion mechanisms. The biological functions of these genes support our findings. Future studies of confirmation of these loci may suggest targets for searching new drugs for controlling smoking and eating behaviors. Sequencing these genes and other genes in relevant pathways may be helpful for identifying funtional variants that have pleiotropic effects on both BMI and smoking behavior.

## Supporting information

S1 Table 241 selected SNPs and the AIC values of different DAGs and minimum AIC values of different categories. (DOCX)

S2 Table The comparison of associations between different BMI-GRSs and smoking categories (n = 17,037). (DOCX)

S3 Table The comparison of partial correlations between different BMI-GRSs and pack-years of smoking in smokers. (DOCX)

S4 Table The comparison of associations between seven candidate pleiotropic SNPs and BMI before and after adjustment for smoking phenotypes. (DOCX)
