# Supplementary material for: Pleiotropy of genetic variants on obesity and smoking phenotypes: Results from the Oncoarray Project of The International Lung Cancer Consortium
Source: PLoS One. 2017 Sep 28;12(9):e0185660. doi: 10.1371/journal.pone.0185660 (PMC5619832; doi:10.1371/journal.pone.0185660)
Supplement: S1 Table — (DOCX) [file pone.0185660.s001.docx]

S1 Table: 241 selected SNPs and the AIC values of different DAGs and minimum AIC values of different categories

| **SNP** | **chrom** | **position** | **gene** | **DAG1** | **DAG2** | **DAG3** | **DAG4** | **DAG5** | **DAG6** | **DAG7** | **DAG8** | **DAG9** | **DAG**  **10** | **DAG**  **11** | **DAG**  **12** | **Category 1** | **Category 2** | **Category 3** | **Category 4** |
| --- | --- | --- | --- | --- | --- | --- | --- | --- | --- | --- | --- | --- | --- | --- | --- | --- | --- | --- | --- |
| **BMI/Smoking status** | | | | | | | | | | | | | | | | | | | |
| rs7550711 | 1 | 110082886 | GPR61 | 40862.0 | 40859.0 | 40859.2 | 40859.9 | 40856.6 | 40856.7 | 40853.6 | 40853.6 | 40857.0 | 40854.4 | 40851.4 | 40851.2 | 40859.0 | 40856.6 | 40853.6 | 40851.2 |
| rs1546924 | 1 | 112273485 | FAM212B | 40862.0 | 40859.0 | 40859.2 | 40864.0 | 40861.3 | 40861.0 | 40858.3 | 40858.5 | 40861.2 | 40863.3 | 40860.5 | 40860.3 | 40859.0 | 40861.0 | 40858.3 | 40860.3 |
| rs4357530 | 1 | 151103153 | SEMA6C | 40862.0 | 40859.0 | 40859.2 | 40863.2 | 40864.0 | 40860.2 | 40861.0 | 40861.2 | 40860.3 | 40865.2 | 40862.3 | 40862.2 | 40859.0 | 40860.2 | 40860.3 | 40862.2 |
| rs4568876 | 1 | 32152518 | COL16A1 | 40862.0 | 40859.0 | 40859.2 | 40859.6 | 40862.1 | 40856.5 | 40859.1 | 40859.1 | 40856.8 | 40859.6 | 40856.7 | 40856.5 | 40859.0 | 40856.5 | 40856.8 | 40856.5 |
| rs4061073 | 1 | 54696743 | SSBP3 | 40862.0 | 40859.0 | 40859.2 | 40863.5 | 40863.9 | 40860.5 | 40860.9 | 40861.0 | 40860.7 | 40865.4 | 40862.5 | 40862.4 | 40859.0 | 40860.5 | 40860.7 | 40862.4 |
| rs2481665 | 1 | 62594677 | INADL | 40862.0 | 40859.0 | 40859.2 | 40863.7 | 40863.7 | 40860.7 | 40860.7 | 40860.9 | 40860.9 | 40865.4 | 40862.5 | 40862.4 | 40859.0 | 40860.7 | 40860.7 | 40862.4 |
| rs11165643 | 1 | 96924097 |  | 40862.0 | 40859.0 | 40859.2 | 40854.7 | 40860.1 | 40851.5 | 40857.1 | 40857.0 | 40851.8 | 40852.7 | 40849.7 | 40849.5 | 40859.0 | 40851.5 | 40851.8 | 40849.5 |
| rs10779751 | 1 | 11284336 | MTOR | 40862.0 | 40859.0 | 40859.2 | 40855.3 | 40862.4 | 40852.2 | 40859.4 | 40859.4 | 40852.5 | 40855.7 | 40852.7 | 40852.5 | 40859.0 | 40852.2 | 40852.5 | 40852.5 |
| rs10920678 | 1 | 190239907 | BRINP3 | 40862.0 | 40859.0 | 40859.2 | 40862.7 | 40863.3 | 40859.7 | 40860.3 | 40860.4 | 40859.8 | 40864.0 | 40861.1 | 40860.9 | 40859.0 | 40859.7 | 40859.8 | 40860.9 |
| rs11583200 | 1 | 50559820 | ELAVL4 | 40862.0 | 40859.0 | 40859.2 | 40857.7 | 40860.7 | 40854.5 | 40857.7 | 40857.7 | 40854.8 | 40856.3 | 40853.4 | 40853.2 | 40859.0 | 40854.5 | 40854.8 | 40853.2 |
| rs1361739 | 1 | 96289734 |  | 40862.0 | 40859.0 | 40859.2 | 40864.0 | 40863.2 | 40861.0 | 40860.2 | 40860.4 | 40861.2 | 40865.2 | 40862.3 | 40862.2 | 40859.0 | 40861.0 | 40860.2 | 40862.2 |
| rs2088518 | 1 | 77951330 | AK5 | 40862.0 | 40859.0 | 40859.2 | 40862.0 | 40861.7 | 40858.9 | 40858.7 | 40858.7 | 40859.2 | 40861.6 | 40858.7 | 40858.6 | 40859.0 | 40858.9 | 40858.7 | 40858.6 |
| rs2275426 | 1 | 46487552 | MAST2 | 40862.0 | 40859.0 | 40859.2 | 40863.5 | 40863.3 | 40860.5 | 40860.3 | 40860.4 | 40860.6 | 40864.7 | 40861.9 | 40861.7 | 40859.0 | 40860.5 | 40860.3 | 40861.7 |
| rs2820292 | 1 | 201784287 | NAV1 | 40862.0 | 40859.0 | 40859.2 | 40860.0 | 40863.5 | 40857.0 | 40860.4 | 40860.6 | 40857.2 | 40861.4 | 40858.6 | 40858.4 | 40859.0 | 40857.0 | 40857.2 | 40858.4 |
| rs284227 | 1 | 82379446 | ADGRL2 | 40862.0 | 40859.0 | 40859.2 | 40858.7 | 40863.2 | 40855.6 | 40860.2 | 40860.2 | 40855.8 | 40859.8 | 40856.9 | 40856.7 | 40859.0 | 40855.6 | 40855.8 | 40856.7 |
| rs4130548 | 1 | 78463868 | DNAJB4 | 40862.0 | 40859.0 | 40859.2 | 40863.3 | 40863.3 | 40860.3 | 40860.3 | 40860.5 | 40860.4 | 40864.6 | 40861.7 | 40861.6 | 40859.0 | 40860.3 | 40860.3 | 40861.6 |
| rs543874 | 1 | 177889480 | SEC16B | 40862.0 | 40859.0 | 40859.2 | 40854.7 | 40863.9 | 40851.7 | 40860.9 | 40861.1 | 40851.9 | 40856.6 | 40853.8 | 40853.6 | 40859.0 | 40851.7 | 40851.9 | 40853.6 |
| rs657452 | 1 | 49589847 | AGBL4 | 40862.0 | 40859.0 | 40859.2 | 40863.6 | 40861.4 | 40860.5 | 40858.3 | 40858.5 | 40860.7 | 40862.9 | 40860.0 | 40859.8 | 40859.0 | 40860.5 | 40858.3 | 40859.8 |
| rs7531118 | 1 | 72837239 |  | 40862.0 | 40859.0 | 40859.2 | 40846.2 | 40863.7 | 40843.3 | 40860.7 | 40861.0 | 40843.4 | 40847.9 | 40845.1 | 40845.0 | 40859.0 | 40843.3 | 40843.4 | 40845.0 |
| rs7551507 | 1 | 74995225 | TNNI3K | 40862.0 | 40859.0 | 40859.2 | 40859.2 | 40863.8 | 40856.1 | 40860.8 | 40860.9 | 40856.3 | 40860.9 | 40858.0 | 40857.9 | 40859.0 | 40856.1 | 40856.3 | 40857.9 |
| rs9660180 | 1 | 1723031 | GNB1 | 40862.0 | 40859.0 | 40859.2 | 40863.9 | 40864.0 | 40860.9 | 40861.0 | 40861.1 | 40861.0 | 40865.8 | 40862.9 | 40862.8 | 40859.0 | 40860.9 | 40861.0 | 40862.8 |
| rs977747 | 1 | 47684677 | TAL1 | 40862.0 | 40859.0 | 40859.2 | 40860.1 | 40863.6 | 40857.0 | 40860.6 | 40860.7 | 40857.2 | 40861.6 | 40858.7 | 40858.6 | 40859.0 | 40857.0 | 40857.2 | 40858.6 |
| rs995258 | 1 | 97431052 |  | 40862.0 | 40859.0 | 40859.2 | 40862.0 | 40863.7 | 40859.0 | 40860.7 | 40860.9 | 40859.2 | 40863.7 | 40860.9 | 40860.7 | 40859.0 | 40859.0 | 40859.2 | 40860.7 |
| rs17406900 | 2 | 203784202 | CARF | 40862.0 | 40859.0 | 40859.2 | 40864.0 | 40862.2 | 40861.0 | 40859.2 | 40859.4 | 40861.2 | 40864.2 | 40861.3 | 40861.2 | 40859.0 | 40861.0 | 40859.2 | 40861.2 |
| rs715 | 2 | 211543055 | CPS1 | 40862.0 | 40859.0 | 40859.2 | 40857.8 | 40863.7 | 40854.8 | 40860.7 | 40860.8 | 40855.0 | 40859.5 | 40856.6 | 40856.4 | 40859.0 | 40854.8 | 40855.0 | 40856.4 |
| rs10182181 | 2 | 25150296 |  | 40862.0 | 40859.0 | 40859.2 | 40853.9 | 40864.0 | 40850.9 | 40861.0 | 40861.2 | 40851.0 | 40855.9 | 40853.0 | 40852.9 | 40859.0 | 40850.9 | 40851.0 | 40852.9 |
| rs1979755 | 2 | 42708405 | KCNG3 | 40862.0 | 40859.0 | 40859.2 | 40862.4 | 40862.8 | 40859.5 | 40859.8 | 40860.0 | 40859.6 | 40863.2 | 40860.4 | 40860.2 | 40859.0 | 40859.5 | 40859.6 | 40860.2 |
| rs1016287 | 2 | 59305625 |  | 40862.0 | 40859.0 | 40859.2 | 40862.9 | 40863.5 | 40859.9 | 40860.5 | 40860.7 | 40860.1 | 40864.4 | 40861.6 | 40861.4 | 40859.0 | 40859.9 | 40860.1 | 40861.4 |
| rs10166736 | 2 | 227900419 | COL4A4 | 40862.0 | 40859.0 | 40859.2 | 40864.0 | 40863.9 | 40861.0 | 40860.9 | 40861.1 | 40861.1 | 40865.9 | 40863.0 | 40862.8 | 40859.0 | 40861.0 | 40860.9 | 40862.8 |
| rs10929925 | 2 | 6155557 |  | 40862.0 | 40859.0 | 40859.2 | 40863.3 | 40863.0 | 40860.4 | 40860.0 | 40860.2 | 40860.5 | 40864.3 | 40861.5 | 40861.3 | 40859.0 | 40860.4 | 40860.0 | 40861.3 |
| rs11126666 | 2 | 26928811 | KCNK3 | 40862.0 | 40859.0 | 40859.2 | 40858.0 | 40864.0 | 40855.0 | 40861.0 | 40861.2 | 40855.1 | 40860.0 | 40857.1 | 40857.0 | 40859.0 | 40855.0 | 40855.1 | 40857.0 |
| rs11677911 | 2 | 237905331 |  | 40862.0 | 40859.0 | 40859.2 | 40864.0 | 40862.3 | 40861.0 | 40859.3 | 40859.5 | 40861.2 | 40864.3 | 40861.4 | 40861.3 | 40859.0 | 40861.0 | 40859.3 | 40861.3 |
| rs12622013 | 2 | 79501362 | CTNNA2 | 40862.0 | 40859.0 | 40859.2 | 40863.6 | 40863.3 | 40860.6 | 40860.3 | 40860.4 | 40860.7 | 40864.8 | 40862.0 | 40861.8 | 40859.0 | 40860.6 | 40860.3 | 40861.8 |
| rs13021737 | 2 | 632348 |  | 40862.0 | 40859.0 | 40859.2 | 40829.6 | 40860.1 | 40826.2 | 40857.0 | 40856.8 | 40826.8 | 40827.6 | 40824.4 | 40824.2 | 40859.0 | 40826.2 | 40826.8 | 40824.2 |
| rs13417156 | 2 | 62848319 | AC092155.4 | 40862.0 | 40859.0 | 40859.2 | 40863.7 | 40864.0 | 40860.7 | 40861.0 | 40861.2 | 40860.8 | 40865.7 | 40862.8 | 40862.7 | 40859.0 | 40860.7 | 40860.8 | 40862.7 |
| rs1460676 | 2 | 164567689 | FIGN | 40862.0 | 40859.0 | 40859.2 | 40863.9 | 40863.5 | 40860.9 | 40860.4 | 40860.6 | 40861.0 | 40865.3 | 40862.5 | 40862.3 | 40859.0 | 40860.9 | 40860.4 | 40862.3 |
| rs1528435 | 2 | 181550962 | AC009478.1 | 40862.0 | 40859.0 | 40859.2 | 40858.8 | 40860.5 | 40855.7 | 40857.5 | 40857.5 | 40856.0 | 40857.3 | 40854.3 | 40854.1 | 40859.0 | 40855.7 | 40856.0 | 40854.1 |
| rs1554622 | 2 | 219606218 | TTLL4 | 40862.0 | 40859.0 | 40859.2 | 40863.6 | 40864.0 | 40860.6 | 40861.0 | 40861.2 | 40860.7 | 40865.6 | 40862.7 | 40862.6 | 40859.0 | 40860.6 | 40860.7 | 40862.6 |
| rs1561277 | 2 | 136092061 | ZRANB3 | 40862.0 | 40859.0 | 40859.2 | 40864.0 | 40863.9 | 40861.0 | 40860.9 | 40861.1 | 40861.2 | 40865.9 | 40863.1 | 40862.9 | 40859.0 | 40861.0 | 40860.9 | 40862.9 |
| rs17203016 | 2 | 208255518 | AC007879.5 | 40862.0 | 40859.0 | 40859.2 | 40864.0 | 40864.0 | 40861.0 | 40861.0 | 40861.2 | 40861.2 | 40866.0 | 40863.2 | 40863.0 | 40859.0 | 40861.0 | 40861.0 | 40863.0 |
| rs2890652 | 2 | 142959931 |  | 40862.0 | 40859.0 | 40859.2 | 40863.6 | 40864.0 | 40860.6 | 40861.0 | 40861.2 | 40860.7 | 40865.5 | 40862.7 | 40862.5 | 40859.0 | 40860.6 | 40860.7 | 40862.5 |
| rs4372836 | 2 | 28973883 | PPP1CB | 40862.0 | 40859.0 | 40859.2 | 40864.0 | 40864.0 | 40861.0 | 40861.0 | 40861.1 | 40861.2 | 40866.0 | 40863.1 | 40863.0 | 40859.0 | 40861.0 | 40861.0 | 40863.0 |
| rs4667682 | 2 | 172127920 |  | 40862.0 | 40859.0 | 40859.2 | 40862.5 | 40863.7 | 40859.5 | 40860.7 | 40860.9 | 40859.6 | 40864.1 | 40861.3 | 40861.1 | 40859.0 | 40859.5 | 40859.6 | 40861.1 |
| rs4988235 | 2 | 136608646 | MCM6 | 40862.0 | 40859.0 | 40859.2 | 40863.9 | 40864.0 | 40860.9 | 40861.0 | 40861.2 | 40861.1 | 40865.9 | 40863.1 | 40862.9 | 40859.0 | 40860.9 | 40861.0 | 40862.9 |
| rs6705646 | 2 | 165567695 | COBLL1 | 40862.0 | 40859.0 | 40859.2 | 40864.0 | 40862.3 | 40861.0 | 40859.3 | 40859.4 | 40861.1 | 40864.2 | 40861.4 | 40861.2 | 40859.0 | 40861.0 | 40859.3 | 40861.2 |
| rs6713510 | 2 | 227034499 |  | 40862.0 | 40859.0 | 40859.2 | 40858.2 | 40862.7 | 40855.1 | 40859.7 | 40859.8 | 40855.4 | 40858.9 | 40856.0 | 40855.8 | 40859.0 | 40855.1 | 40855.4 | 40855.8 |
| rs7599312 | 2 | 213413231 |  | 40862.0 | 40859.0 | 40859.2 | 40864.0 | 40864.0 | 40861.0 | 40861.0 | 40861.1 | 40861.1 | 40865.9 | 40863.0 | 40862.9 | 40859.0 | 40861.0 | 40861.0 | 40862.9 |
| rs929641 | 2 | 58792377 | LINC01122 | 40862.0 | 40859.0 | 40859.2 | 40856.0 | 40857.0 | 40852.7 | 40854.0 | 40853.9 | 40853.1 | 40851.0 | 40847.8 | 40847.7 | 40859.0 | 40852.7 | 40853.1 | 40847.7 |
| rs968059 | 2 | 35404011 | AC012593.1 | 40862.0 | 40859.0 | 40859.2 | 40863.9 | 40863.4 | 40860.8 | 40860.4 | 40860.6 | 40861.0 | 40865.2 | 40862.4 | 40862.2 | 40859.0 | 40860.8 | 40860.4 | 40862.2 |
| rs972540 | 2 | 207244783 |  | 40862.0 | 40859.0 | 40859.2 | 40863.7 | 40863.1 | 40860.7 | 40860.1 | 40860.3 | 40860.9 | 40864.8 | 40862.0 | 40861.8 | 40859.0 | 40860.7 | 40860.1 | 40861.8 |
| rs7640424 | 3 | 107820063 |  | 40862.0 | 40859.0 | 40859.2 | 40864.0 | 40863.9 | 40861.0 | 40860.9 | 40861.1 | 40861.2 | 40865.9 | 40863.1 | 40862.9 | 40859.0 | 40861.0 | 40860.9 | 40862.9 |
| rs2124499 | 3 | 123093541 | ADCY5 | 40862.0 | 40859.0 | 40859.2 | 40863.8 | 40863.1 | 40860.8 | 40860.1 | 40860.2 | 40861.0 | 40864.9 | 40862.0 | 40861.8 | 40859.0 | 40860.8 | 40860.1 | 40861.8 |
| rs7620457 | 3 | 183747266 | HTR3D | 40862.0 | 40859.0 | 40859.2 | 40861.9 | 40862.2 | 40858.9 | 40859.2 | 40859.4 | 40859.0 | 40862.0 | 40859.2 | 40859.1 | 40859.0 | 40858.9 | 40859.0 | 40859.1 |
| rs2710323 | 3 | 52815905 | ITIH1 | 40862.0 | 40859.0 | 40859.2 | 40861.1 | 40861.1 | 40857.9 | 40858.1 | 40858.2 | 40858.2 | 40860.2 | 40857.2 | 40857.0 | 40859.0 | 40857.9 | 40858.1 | 40857.0 |
| rs2612012 | 3 | 53745625 | CACNA1D | 40862.0 | 40859.0 | 40859.2 | 40856.1 | 40863.8 | 40853.1 | 40860.8 | 40860.9 | 40853.3 | 40857.9 | 40855.0 | 40854.8 | 40859.0 | 40853.1 | 40853.3 | 40854.8 |
| rs2371767 | 3 | 64718258 | ADAMTS9-AS2 | 40862.0 | 40859.0 | 40859.2 | 40857.0 | 40863.0 | 40853.9 | 40860.0 | 40860.0 | 40854.1 | 40857.9 | 40855.0 | 40854.8 | 40859.0 | 40853.9 | 40854.1 | 40854.8 |
| rs13078960 | 3 | 85807590 | CADM2 | 40862.0 | 40859.0 | 40859.2 | 40863.8 | 40860.1 | 40860.7 | 40857.1 | 40857.2 | 40860.9 | 40861.9 | 40859.0 | 40858.8 | 40859.0 | 40860.1 | 40857.1 | 40858.8 |
| rs1436351 | 3 | 104617973 |  | 40862.0 | 40859.0 | 40859.2 | 40854.5 | 40864.0 | 40851.5 | 40861.0 | 40861.2 | 40851.6 | 40856.4 | 40853.6 | 40853.4 | 40859.0 | 40851.5 | 40851.6 | 40853.4 |
| rs1516725 | 3 | 185824004 | ETV5 | 40862.0 | 40859.0 | 40859.2 | 40853.7 | 40863.6 | 40850.8 | 40860.6 | 40860.9 | 40850.8 | 40855.3 | 40852.5 | 40852.3 | 40859.0 | 40850.8 | 40850.8 | 40852.3 |
| rs16851483 | 3 | 141275436 | RASA2 | 40862.0 | 40859.0 | 40859.2 | 40862.7 | 40861.3 | 40859.7 | 40858.3 | 40858.6 | 40859.8 | 40862.0 | 40859.2 | 40859.0 | 40859.0 | 40859.7 | 40858.3 | 40859.0 |
| rs2365389 | 3 | 61236462 | FHIT | 40862.0 | 40859.0 | 40859.2 | 40859.0 | 40864.0 | 40856.0 | 40861.0 | 40861.1 | 40856.1 | 40861.0 | 40858.1 | 40857.9 | 40859.0 | 40856.0 | 40856.1 | 40857.9 |
| rs3849570 | 3 | 81792112 | GBE1 | 40862.0 | 40859.0 | 40859.2 | 40863.0 | 40863.6 | 40860.0 | 40860.6 | 40860.7 | 40860.2 | 40864.5 | 40861.7 | 40861.6 | 40859.0 | 40860.0 | 40860.2 | 40861.6 |
| rs4395360 | 3 | 157318257 | PQLC2L | 40862.0 | 40859.0 | 40859.2 | 40861.4 | 40863.0 | 40858.4 | 40860.0 | 40860.1 | 40858.6 | 40862.4 | 40859.5 | 40859.3 | 40859.0 | 40858.4 | 40858.6 | 40859.3 |
| rs6804842 | 3 | 25106437 | AC133680.1 | 40862.0 | 40859.0 | 40859.2 | 40862.3 | 40855.8 | 40859.5 | 40852.8 | 40853.1 | 40859.5 | 40856.1 | 40853.4 | 40853.2 | 40859.0 | 40855.8 | 40852.8 | 40853.2 |
| rs7611238 | 3 | 195072918 | ACAP2 | 40862.0 | 40859.0 | 40859.2 | 40857.5 | 40864.0 | 40854.5 | 40861.0 | 40861.2 | 40854.7 | 40859.5 | 40856.6 | 40856.5 | 40859.0 | 40854.5 | 40854.7 | 40856.5 |
| rs7613875 | 3 | 49971514 | MON1A | 40862.0 | 40859.0 | 40859.2 | 40863.9 | 40864.0 | 40860.9 | 40861.0 | 40861.1 | 40861.0 | 40865.8 | 40863.0 | 40862.8 | 40859.0 | 40860.9 | 40861.0 | 40862.8 |
| rs7649970 | 3 | 12392272 | PPARG | 40862.0 | 40859.0 | 40859.2 | 40859.8 | 40864.0 | 40856.8 | 40861.0 | 40861.2 | 40857.0 | 40861.8 | 40859.0 | 40858.8 | 40859.0 | 40856.8 | 40857.0 | 40858.8 |
| rs876424 | 3 | 131637676 | CPNE4 | 40862.0 | 40859.0 | 40859.2 | 40860.7 | 40863.9 | 40857.6 | 40860.9 | 40861.1 | 40857.8 | 40862.5 | 40859.7 | 40859.5 | 40859.0 | 40857.6 | 40857.8 | 40859.5 |
| rs9867325 | 3 | 136618909 | NCK1 | 40862.0 | 40859.0 | 40859.2 | 40864.0 | 40862.7 | 40860.9 | 40859.7 | 40859.8 | 40861.1 | 40864.6 | 40861.7 | 40861.6 | 40859.0 | 40860.9 | 40859.7 | 40861.6 |
| rs9880211 | 3 | 136107549 | STAG1 | 40862.0 | 40859.0 | 40859.2 | 40863.4 | 40862.7 | 40860.4 | 40859.7 | 40859.8 | 40860.6 | 40864.1 | 40861.2 | 40861.1 | 40859.0 | 40860.4 | 40859.7 | 40861.1 |
| rs13107325 | 4 | 103188709 | SLC39A8 | 40862.0 | 40859.0 | 40859.2 | 40855.3 | 40863.4 | 40852.3 | 40860.4 | 40860.6 | 40852.4 | 40856.6 | 40853.9 | 40853.7 | 40859.0 | 40852.3 | 40852.4 | 40853.7 |
| rs11727676 | 4 | 145659064 | HHIP | 40862.0 | 40859.0 | 40859.2 | 40863.2 | 40862.4 | 40860.2 | 40859.4 | 40859.6 | 40860.3 | 40863.5 | 40860.7 | 40860.6 | 40859.0 | 40860.2 | 40859.4 | 40860.6 |
| rs4833079 | 4 | 38654681 |  | 40862.0 | 40859.0 | 40859.2 | 40859.6 | 40863.0 | 40856.7 | 40859.9 | 40860.2 | 40856.8 | 40860.5 | 40857.8 | 40857.6 | 40859.0 | 40856.7 | 40856.8 | 40857.6 |
| rs10009336 | 4 | 44480783 |  | 40862.0 | 40859.0 | 40859.2 | 40862.5 | 40864.0 | 40859.5 | 40861.0 | 40861.2 | 40859.7 | 40864.5 | 40861.6 | 40861.5 | 40859.0 | 40859.5 | 40859.7 | 40861.5 |
| rs17001654 | 4 | 77129568 | SCARB2 | 40862.0 | 40859.0 | 40859.2 | 40864.0 | 40862.9 | 40861.0 | 40859.9 | 40860.0 | 40861.1 | 40864.8 | 40861.9 | 40861.8 | 40859.0 | 40861.0 | 40859.9 | 40861.8 |
| rs13130484 | 4 | 45175691 |  | 40862.0 | 40859.0 | 40859.2 | 40842.5 | 40862.3 | 40839.7 | 40859.3 | 40859.7 | 40839.6 | 40842.8 | 40840.1 | 40840.0 | 40859.0 | 40839.7 | 40839.6 | 40840.0 |
| rs2391518 | 4 | 130817060 | RP11-422J15.1 | 40862.0 | 40859.0 | 40859.2 | 40863.2 | 40864.0 | 40860.2 | 40861.0 | 40861.2 | 40860.3 | 40865.2 | 40862.3 | 40862.1 | 40859.0 | 40860.2 | 40860.3 | 40862.1 |
| rs6864049 | 5 | 124330522 |  | 40862.0 | 40859.0 | 40859.2 | 40862.0 | 40862.2 | 40858.9 | 40859.2 | 40859.2 | 40859.1 | 40862.1 | 40859.2 | 40859.0 | 40859.0 | 40858.9 | 40859.1 | 40859.0 |
| rs6870983 | 5 | 87697533 | TMEM161B-AS1 | 40862.0 | 40859.0 | 40859.2 | 40857.6 | 40863.8 | 40854.5 | 40860.8 | 40860.9 | 40854.7 | 40859.3 | 40856.4 | 40856.3 | 40859.0 | 40854.5 | 40854.7 | 40856.3 |
| rs11951673 | 5 | 95861012 | CAST | 40862.0 | 40859.0 | 40859.2 | 40853.0 | 40864.0 | 40849.9 | 40861.0 | 40861.2 | 40850.1 | 40855.0 | 40852.1 | 40851.9 | 40859.0 | 40849.9 | 40850.1 | 40851.9 |
| rs150992 | 5 | 98275197 | CTD-2007H13.3 | 40862.0 | 40859.0 | 40859.2 | 40864.0 | 40864.0 | 40861.0 | 40861.0 | 40861.2 | 40861.1 | 40865.9 | 40863.1 | 40862.9 | 40859.0 | 40861.0 | 40861.0 | 40862.9 |
| rs2112347 | 5 | 75015242 | POC5 | 40862.0 | 40859.0 | 40859.2 | 40863.7 | 40864.0 | 40860.7 | 40861.0 | 40861.2 | 40860.8 | 40865.7 | 40862.8 | 40862.7 | 40859.0 | 40860.7 | 40860.8 | 40862.7 |
| rs288232 | 5 | 107419548 | FBXL17 | 40862.0 | 40859.0 | 40859.2 | 40863.8 | 40864.0 | 40860.8 | 40861.0 | 40861.2 | 40861.0 | 40865.8 | 40862.9 | 40862.8 | 40859.0 | 40860.8 | 40861.0 | 40862.8 |
| rs7715256 | 5 | 153537893 | MFAP3 | 40862.0 | 40859.0 | 40859.2 | 40851.9 | 40864.0 | 40848.9 | 40861.0 | 40861.2 | 40849.0 | 40853.9 | 40851.0 | 40850.9 | 40859.0 | 40848.9 | 40849.0 | 40850.9 |
| rs6569648 | 6 | 130349119 | L3MBTL3 | 40862.0 | 40859.0 | 40859.2 | 40863.8 | 40856.8 | 40860.7 | 40853.8 | 40853.9 | 40860.9 | 40858.5 | 40855.6 | 40855.5 | 40859.0 | 40856.8 | 40853.8 | 40855.5 |
| rs13201877 | 6 | 137675541 |  | 40862.0 | 40859.0 | 40859.2 | 40858.1 | 40862.0 | 40855.0 | 40859.0 | 40859.1 | 40855.2 | 40858.1 | 40855.1 | 40855.0 | 40859.0 | 40855.0 | 40855.2 | 40855.0 |
| rs13191362 | 6 | 163033350 | PARK2 | 40862.0 | 40859.0 | 40859.2 | 40860.5 | 40863.8 | 40857.5 | 40860.8 | 40861.0 | 40857.6 | 40862.2 | 40859.4 | 40859.3 | 40859.0 | 40857.5 | 40857.6 | 40859.3 |
| rs943466 | 6 | 33731787 |  | 40862.0 | 40859.0 | 40859.2 | 40863.9 | 40863.9 | 40860.9 | 40860.9 | 40861.0 | 40861.1 | 40865.7 | 40862.9 | 40862.7 | 40859.0 | 40860.9 | 40860.9 | 40862.7 |
| rs206936 | 6 | 34302869 | NUDT3 | 40862.0 | 40859.0 | 40859.2 | 40854.2 | 40863.4 | 40851.3 | 40860.4 | 40860.6 | 40851.4 | 40855.5 | 40852.8 | 40852.6 | 40859.0 | 40851.3 | 40851.4 | 40852.6 |
| rs1358980 | 6 | 43764551 |  | 40862.0 | 40859.0 | 40859.2 | 40863.5 | 40864.0 | 40860.5 | 40861.0 | 40861.2 | 40860.6 | 40865.5 | 40862.6 | 40862.5 | 40859.0 | 40860.5 | 40860.6 | 40862.5 |
| rs17665162 | 6 | 50275258 |  | 40862.0 | 40859.0 | 40859.2 | 40862.3 | 40863.9 | 40859.3 | 40860.9 | 40861.1 | 40859.4 | 40864.2 | 40861.3 | 40861.2 | 40859.0 | 40859.3 | 40859.4 | 40861.2 |
| rs200807 | 6 | 97903674 |  | 40862.0 | 40859.0 | 40859.2 | 40863.6 | 40856.5 | 40860.5 | 40853.5 | 40853.6 | 40860.7 | 40858.0 | 40855.1 | 40854.9 | 40859.0 | 40856.5 | 40853.5 | 40854.9 |
| rs2033529 | 6 | 40348653 |  | 40862.0 | 40859.0 | 40859.2 | 40859.2 | 40863.7 | 40856.2 | 40860.7 | 40860.8 | 40856.4 | 40860.9 | 40858.0 | 40857.8 | 40859.0 | 40856.2 | 40856.4 | 40857.8 |
| rs2228213 | 6 | 12124855 | HIVEP1 | 40862.0 | 40859.0 | 40859.2 | 40864.0 | 40863.9 | 40861.0 | 40860.9 | 40861.0 | 40861.1 | 40865.8 | 40863.0 | 40862.8 | 40859.0 | 40861.0 | 40860.9 | 40862.8 |
| rs3800229 | 6 | 108996963 | FOXO3 | 40862.0 | 40859.0 | 40859.2 | 40857.9 | 40861.3 | 40854.7 | 40858.3 | 40858.2 | 40855.0 | 40857.1 | 40854.0 | 40853.9 | 40859.0 | 40854.7 | 40855.0 | 40853.9 |
| rs539958 | 6 | 160772842 | SLC22A3 | 40862.0 | 40859.0 | 40859.2 | 40864.0 | 40857.6 | 40861.0 | 40854.6 | 40854.8 | 40861.2 | 40859.6 | 40856.7 | 40856.5 | 40859.0 | 40857.6 | 40854.6 | 40856.5 |
| rs6457796 | 6 | 34828553 | UHRF1BP1 | 40862.0 | 40859.0 | 40859.2 | 40860.6 | 40863.8 | 40857.5 | 40860.8 | 40860.9 | 40857.7 | 40862.4 | 40859.5 | 40859.3 | 40859.0 | 40857.5 | 40857.7 | 40859.3 |
| rs6903387 | 6 | 46348834 | RCAN2 | 40862.0 | 40859.0 | 40859.2 | 40862.5 | 40864.0 | 40859.5 | 40861.0 | 40861.2 | 40859.7 | 40864.5 | 40861.7 | 40861.5 | 40859.0 | 40859.5 | 40859.7 | 40861.5 |
| rs9275595 | 6 | 32681355 | XXbac-BPG254F23.7 | 40862.0 | 40859.0 | 40859.2 | 40858.3 | 40855.1 | 40855.1 | 40852.1 | 40852.0 | 40855.5 | 40851.4 | 40848.3 | 40848.2 | 40859.0 | 40855.1 | 40852.0 | 40848.2 |
| rs9364687 | 6 | 163817911 |  | 40862.0 | 40859.0 | 40859.2 | 40863.1 | 40864.0 | 40860.0 | 40861.0 | 40861.1 | 40860.2 | 40865.0 | 40862.1 | 40862.0 | 40859.0 | 40860.0 | 40860.2 | 40862.0 |
| rs9374842 | 6 | 120185665 |  | 40862.0 | 40859.0 | 40859.2 | 40863.2 | 40863.1 | 40860.3 | 40860.1 | 40860.2 | 40860.4 | 40864.3 | 40861.4 | 40861.3 | 40859.0 | 40860.3 | 40860.1 | 40861.3 |
| rs943005 | 6 | 50865820 | RP4-753D5.3 | 40862.0 | 40859.0 | 40859.2 | 40862.4 | 40864.0 | 40859.4 | 40861.0 | 40861.2 | 40859.6 | 40864.4 | 40861.5 | 40861.4 | 40859.0 | 40859.4 | 40859.6 | 40861.4 |
| rs11771526 | 7 | 32342618 | PDE1C | 40862.0 | 40859.0 | 40859.2 | 40864.0 | 40863.8 | 40861.0 | 40860.8 | 40860.9 | 40861.2 | 40865.7 | 40862.9 | 40862.7 | 40859.0 | 40861.0 | 40860.8 | 40862.7 |
| rs10269783 | 7 | 49616203 |  | 40862.0 | 40859.0 | 40859.2 | 40863.6 | 40863.9 | 40860.6 | 40860.9 | 40861.0 | 40860.8 | 40865.5 | 40862.6 | 40862.4 | 40859.0 | 40860.6 | 40860.8 | 40862.4 |
| rs2245368 | 7 | 76608143 | UPK3B | 40862.0 | 40859.0 | 40859.2 | 40847.4 | 40862.5 | 40844.2 | 40859.5 | 40859.4 | 40844.6 | 40847.8 | 40844.8 | 40844.6 | 40859.0 | 40844.2 | 40844.6 | 40844.6 |
| rs9641123 | 7 | 93197732 | CALCR | 40862.0 | 40859.0 | 40859.2 | 40852.1 | 40863.7 | 40849.0 | 40860.7 | 40860.8 | 40849.3 | 40853.8 | 40850.8 | 40850.7 | 40859.0 | 40849.0 | 40849.3 | 40850.7 |
| rs6465468 | 7 | 95169514 | ASB4 | 40862.0 | 40859.0 | 40859.2 | 40863.9 | 40864.0 | 40860.9 | 40861.0 | 40861.2 | 40861.0 | 40865.9 | 40863.0 | 40862.9 | 40859.0 | 40860.9 | 40861.0 | 40862.9 |
| rs10499694 | 7 | 50614173 | DDC | 40862.0 | 40859.0 | 40859.2 | 40863.0 | 40863.2 | 40860.0 | 40860.2 | 40860.3 | 40860.2 | 40864.1 | 40861.3 | 40861.1 | 40859.0 | 40860.0 | 40860.2 | 40861.1 |
| rs1167827 | 7 | 75163169 | HIP1 | 40862.0 | 40859.0 | 40859.2 | 40863.0 | 40859.1 | 40859.9 | 40856.1 | 40856.2 | 40860.1 | 40860.0 | 40857.1 | 40857.0 | 40859.0 | 40859.1 | 40856.1 | 40857.0 |
| rs1593312 | 7 | 131584453 | AC009518.4 | 40862.0 | 40859.0 | 40859.2 | 40859.6 | 40864.0 | 40856.6 | 40861.0 | 40861.2 | 40856.7 | 40861.5 | 40858.7 | 40858.6 | 40859.0 | 40856.6 | 40856.7 | 40858.6 |
| rs1830074 | 7 | 6718674 | RP11-611L7.2 | 40862.0 | 40859.0 | 40859.2 | 40864.0 | 40862.0 | 40861.0 | 40859.0 | 40859.1 | 40861.2 | 40864.0 | 40861.1 | 40861.0 | 40859.0 | 40861.0 | 40859.0 | 40861.0 |
| rs3779273 | 7 | 77828940 | MAGI2 | 40862.0 | 40859.0 | 40859.2 | 40863.7 | 40862.5 | 40860.7 | 40859.5 | 40859.6 | 40860.8 | 40864.1 | 40861.3 | 40861.1 | 40859.0 | 40860.7 | 40859.5 | 40861.1 |
| rs6990042 | 8 | 14173974 | SGCZ | 40862.0 | 40859.0 | 40859.2 | 40859.6 | 40858.6 | 40856.8 | 40855.6 | 40856.0 | 40856.8 | 40856.2 | 40853.5 | 40853.4 | 40859.0 | 40856.8 | 40855.6 | 40853.4 |
| rs11997175 | 8 | 33770070 | RP11-317N12.1 | 40862.0 | 40859.0 | 40859.2 | 40864.0 | 40864.0 | 40861.0 | 40861.0 | 40861.1 | 40861.1 | 40865.9 | 40863.1 | 40862.9 | 40859.0 | 40861.0 | 40861.0 | 40862.9 |
| rs11787111 | 8 | 65192451 | RP11-32K4.1 | 40862.0 | 40859.0 | 40859.2 | 40861.2 | 40850.5 | 40858.0 | 40847.5 | 40847.4 | 40858.4 | 40849.7 | 40846.5 | 40846.4 | 40859.0 | 40850.5 | 40847.4 | 40846.4 |
| rs16907751 | 8 | 81375457 |  | 40862.0 | 40859.0 | 40859.2 | 40861.5 | 40864.0 | 40858.5 | 40861.0 | 40861.1 | 40858.6 | 40863.4 | 40860.6 | 40860.4 | 40859.0 | 40858.5 | 40858.6 | 40860.4 |
| rs12680842 | 8 | 95582606 | RP11-267M23.4 | 40862.0 | 40859.0 | 40859.2 | 40863.5 | 40863.8 | 40860.5 | 40860.8 | 40860.9 | 40860.7 | 40865.3 | 40862.4 | 40862.2 | 40859.0 | 40860.5 | 40860.7 | 40862.2 |
| rs10156366 | 8 | 77246607 |  | 40862.0 | 40859.0 | 40859.2 | 40859.8 | 40861.9 | 40856.9 | 40858.9 | 40859.2 | 40856.9 | 40859.6 | 40856.9 | 40856.7 | 40859.0 | 40856.9 | 40856.9 | 40856.7 |
| rs17149279 | 8 | 9195638 | RP11-115J16.1 | 40862.0 | 40859.0 | 40859.2 | 40862.8 | 40864.0 | 40859.8 | 40861.0 | 40861.2 | 40860.0 | 40864.8 | 40862.0 | 40861.8 | 40859.0 | 40859.8 | 40860.0 | 40861.8 |
| rs2060604 | 8 | 76650334 |  | 40862.0 | 40859.0 | 40859.2 | 40850.7 | 40864.0 | 40847.7 | 40861.0 | 40861.2 | 40847.8 | 40852.7 | 40849.8 | 40849.7 | 40859.0 | 40847.7 | 40847.8 | 40849.7 |
| rs3134353 | 8 | 101947453 | YWHAZ | 40862.0 | 40859.0 | 40859.2 | 40864.0 | 40862.5 | 40861.0 | 40859.5 | 40859.7 | 40861.2 | 40864.5 | 40861.7 | 40861.5 | 40859.0 | 40861.0 | 40859.5 | 40861.5 |
| rs4389974 | 8 | 112381796 |  | 40862.0 | 40859.0 | 40859.2 | 40860.9 | 40862.7 | 40857.8 | 40859.7 | 40859.8 | 40858.0 | 40861.5 | 40858.6 | 40858.4 | 40859.0 | 40857.8 | 40858.0 | 40858.4 |
| rs6985539 | 8 | 62081223 | CLVS1 | 40862.0 | 40859.0 | 40859.2 | 40862.9 | 40859.3 | 40859.8 | 40856.3 | 40856.4 | 40860.1 | 40860.2 | 40857.3 | 40857.1 | 40859.0 | 40859.3 | 40856.3 | 40857.1 |
| rs733594 | 8 | 85077686 | RP11-120I21.3 | 40862.0 | 40859.0 | 40859.2 | 40859.8 | 40863.1 | 40856.8 | 40860.1 | 40860.3 | 40856.9 | 40860.8 | 40858.0 | 40857.9 | 40859.0 | 40856.8 | 40856.9 | 40857.9 |
| rs7844647 | 8 | 34503776 |  | 40862.0 | 40859.0 | 40859.2 | 40863.9 | 40864.0 | 40860.9 | 40861.0 | 40861.2 | 40861.1 | 40865.9 | 40863.0 | 40862.9 | 40859.0 | 40860.9 | 40861.0 | 40862.9 |
| rs10760279 | 9 | 126105291 |  | 40862.0 | 40859.0 | 40859.2 | 40859.5 | 40861.9 | 40856.5 | 40858.9 | 40859.2 | 40856.6 | 40859.3 | 40856.6 | 40856.4 | 40859.0 | 40856.5 | 40856.6 | 40856.4 |
| rs10733682 | 9 | 129460914 | LMX1B | 40862.0 | 40859.0 | 40859.2 | 40856.9 | 40863.9 | 40853.9 | 40860.9 | 40861.1 | 40854.1 | 40858.7 | 40855.9 | 40855.8 | 40859.0 | 40853.9 | 40854.1 | 40855.8 |
| rs2270204 | 9 | 131042734 | SWI5 | 40862.0 | 40859.0 | 40859.2 | 40862.9 | 40863.0 | 40860.0 | 40859.9 | 40860.1 | 40860.1 | 40863.8 | 40861.0 | 40860.9 | 40859.0 | 40860.0 | 40859.9 | 40860.9 |
| rs10975870 | 9 | 6880263 | KDM4C | 40862.0 | 40859.0 | 40859.2 | 40863.1 | 40864.0 | 40860.1 | 40861.0 | 40861.1 | 40860.3 | 40865.0 | 40862.2 | 40862.1 | 40859.0 | 40860.1 | 40860.3 | 40862.1 |
| rs10971721 | 9 | 33827694 | UBE2R2 | 40862.0 | 40859.0 | 40859.2 | 40864.0 | 40859.9 | 40861.0 | 40856.9 | 40857.1 | 40861.1 | 40861.9 | 40859.1 | 40858.9 | 40859.0 | 40859.9 | 40856.9 | 40858.9 |
| rs1928295 | 9 | 120378483 |  | 40862.0 | 40859.0 | 40859.2 | 40858.9 | 40862.6 | 40855.8 | 40859.6 | 40859.6 | 40856.0 | 40859.4 | 40856.4 | 40856.3 | 40859.0 | 40855.8 | 40856.0 | 40856.3 |
| rs2183825 | 9 | 28412375 | LINGO2 | 40862.0 | 40859.0 | 40859.2 | 40859.8 | 40863.6 | 40856.9 | 40860.6 | 40860.8 | 40857.0 | 40861.4 | 40858.6 | 40858.4 | 40859.0 | 40856.9 | 40857.0 | 40858.4 |
| rs4740619 | 9 | 15634326 | CCDC171 | 40862.0 | 40859.0 | 40859.2 | 40863.1 | 40861.7 | 40860.1 | 40858.7 | 40858.9 | 40860.2 | 40862.7 | 40859.9 | 40859.8 | 40859.0 | 40860.1 | 40858.7 | 40859.8 |
| rs6477694 | 9 | 111932342 | FRRS1L | 40862.0 | 40859.0 | 40859.2 | 40859.3 | 40863.3 | 40856.2 | 40860.2 | 40860.3 | 40856.4 | 40860.5 | 40857.5 | 40857.4 | 40859.0 | 40856.2 | 40856.4 | 40857.4 |
| rs17094222 | 10 | 102395440 |  | 40862.0 | 40859.0 | 40859.2 | 40857.2 | 40862.4 | 40854.3 | 40859.4 | 40859.7 | 40854.3 | 40857.5 | 40854.8 | 40854.6 | 40859.0 | 40854.3 | 40854.3 | 40854.6 |
| rs718948 | 10 | 126739338 | CTBP2 | 40862.0 | 40859.0 | 40859.2 | 40859.9 | 40863.9 | 40856.9 | 40860.9 | 40861.0 | 40857.0 | 40861.7 | 40858.9 | 40858.7 | 40859.0 | 40856.9 | 40857.0 | 40858.7 |
| rs11191343 | 10 | 104345225 | SUFU | 40862.0 | 40859.0 | 40859.2 | 40863.2 | 40862.3 | 40860.1 | 40859.3 | 40859.4 | 40860.3 | 40863.4 | 40860.5 | 40860.4 | 40859.0 | 40860.1 | 40859.3 | 40860.4 |
| rs12220375 | 10 | 104901491 | NT5C2 | 40862.0 | 40859.0 | 40859.2 | 40857.0 | 40858.2 | 40853.8 | 40855.2 | 40855.1 | 40854.2 | 40853.1 | 40850.1 | 40849.9 | 40859.0 | 40853.8 | 40854.2 | 40849.9 |
| rs751008 | 10 | 129142417 | DOCK1 | 40862.0 | 40859.0 | 40859.2 | 40863.8 | 40863.8 | 40860.8 | 40860.8 | 40860.9 | 40860.9 | 40865.5 | 40862.7 | 40862.5 | 40859.0 | 40860.8 | 40860.8 | 40862.5 |
| rs7899106 | 10 | 87410904 | GRID1 | 40862.0 | 40859.0 | 40859.2 | 40861.4 | 40864.0 | 40858.4 | 40861.0 | 40861.2 | 40858.6 | 40863.4 | 40860.6 | 40860.4 | 40859.0 | 40858.4 | 40858.6 | 40860.4 |
| rs7903146 | 10 | 114758349 | TCF7L2 | 40862.0 | 40859.0 | 40859.2 | 40855.8 | 40864.0 | 40852.8 | 40861.0 | 40861.2 | 40852.9 | 40857.8 | 40854.9 | 40854.8 | 40859.0 | 40852.8 | 40852.9 | 40854.8 |
| rs1557765 | 11 | 17403639 | NCR3LG1 | 40862.0 | 40859.0 | 40859.2 | 40863.9 | 40863.4 | 40860.9 | 40860.4 | 40860.5 | 40861.1 | 40865.3 | 40862.4 | 40862.2 | 40859.0 | 40860.9 | 40860.4 | 40862.2 |
| rs10742752 | 11 | 45438374 | RP11-430H10.4 | 40862.0 | 40859.0 | 40859.2 | 40857.0 | 40864.0 | 40854.0 | 40861.0 | 40861.1 | 40854.2 | 40858.9 | 40856.1 | 40856.0 | 40859.0 | 40854.0 | 40854.2 | 40856.0 |
| rs10540 | 11 | 494662 | RNH1 | 40862.0 | 40859.0 | 40859.2 | 40863.9 | 40862.3 | 40860.8 | 40859.3 | 40859.5 | 40861.0 | 40864.2 | 40861.3 | 40861.1 | 40859.0 | 40860.8 | 40859.3 | 40861.1 |
| rs10840100 | 11 | 8669437 | TRIM66 | 40862.0 | 40859.0 | 40859.2 | 40859.9 | 40861.9 | 40856.8 | 40858.9 | 40859.0 | 40857.0 | 40859.8 | 40856.8 | 40856.7 | 40859.0 | 40856.8 | 40857.0 | 40856.7 |
| rs11030104 | 11 | 27684517 | BDNF | 40862.0 | 40859.0 | 40859.2 | 40859.4 | 40857.7 | 40856.2 | 40854.7 | 40854.7 | 40856.5 | 40855.1 | 40852.1 | 40851.9 | 40859.0 | 40856.2 | 40854.7 | 40851.9 |
| rs11607976 | 11 | 69279111 |  | 40862.0 | 40859.0 | 40859.2 | 40862.2 | 40863.8 | 40859.2 | 40860.8 | 40860.9 | 40859.4 | 40864.0 | 40861.1 | 40861.0 | 40859.0 | 40859.2 | 40859.4 | 40861.0 |
| rs12286929 | 11 | 115022404 |  | 40862.0 | 40859.0 | 40859.2 | 40853.0 | 40863.5 | 40850.0 | 40860.5 | 40860.7 | 40850.1 | 40854.4 | 40851.6 | 40851.5 | 40859.0 | 40850.0 | 40850.1 | 40851.5 |
| rs1552224 | 11 | 72433098 | ARAP1 | 40862.0 | 40859.0 | 40859.2 | 40860.3 | 40864.0 | 40857.3 | 40861.0 | 40861.2 | 40857.5 | 40862.3 | 40859.4 | 40859.3 | 40859.0 | 40857.3 | 40857.5 | 40859.3 |
| rs1816537 | 11 | 112968651 | NCAM1 | 40862.0 | 40859.0 | 40859.2 | 40863.4 | 40860.6 | 40860.4 | 40857.6 | 40857.8 | 40860.5 | 40861.9 | 40859.1 | 40859.0 | 40859.0 | 40860.4 | 40857.6 | 40859.0 |
| rs2176598 | 11 | 43864278 | HSD17B12 | 40862.0 | 40859.0 | 40859.2 | 40863.4 | 40864.0 | 40860.4 | 40861.0 | 40861.2 | 40860.6 | 40865.4 | 40862.5 | 40862.4 | 40859.0 | 40860.4 | 40860.6 | 40862.4 |
| rs2845885 | 11 | 63869062 | MACROD1 | 40862.0 | 40859.0 | 40859.2 | 40864.0 | 40863.9 | 40861.0 | 40860.9 | 40861.1 | 40861.2 | 40865.9 | 40863.1 | 40862.9 | 40859.0 | 40861.0 | 40860.9 | 40862.9 |
| rs3817334 | 11 | 47650993 | MTCH2 | 40862.0 | 40859.0 | 40859.2 | 40852.0 | 40860.4 | 40849.2 | 40857.3 | 40857.7 | 40849.1 | 40850.3 | 40847.6 | 40847.5 | 40859.0 | 40849.2 | 40849.1 | 40847.5 |
| rs4757142 | 11 | 13325695 | ARNTL | 40862.0 | 40859.0 | 40859.2 | 40858.9 | 40863.0 | 40855.9 | 40860.0 | 40860.2 | 40856.0 | 40859.8 | 40857.0 | 40856.9 | 40859.0 | 40855.9 | 40856.0 | 40856.9 |
| rs17033633 | 12 | 103656343 | C12orf42 | 40862.0 | 40859.0 | 40859.2 | 40864.0 | 40863.0 | 40861.0 | 40860.0 | 40860.2 | 40861.2 | 40865.0 | 40862.2 | 40862.0 | 40859.0 | 40861.0 | 40860.0 | 40862.0 |
| rs1502337 | 12 | 111062852 | TCTN1 | 40862.0 | 40859.0 | 40859.2 | 40859.4 | 40863.9 | 40856.4 | 40860.9 | 40861.1 | 40856.5 | 40861.2 | 40858.4 | 40858.2 | 40859.0 | 40856.4 | 40856.5 | 40858.2 |
| rs11057405 | 12 | 122781897 | CLIP1 | 40862.0 | 40859.0 | 40859.2 | 40863.4 | 40864.0 | 40860.4 | 40861.0 | 40861.2 | 40860.6 | 40865.4 | 40862.6 | 40862.4 | 40859.0 | 40860.4 | 40860.6 | 40862.4 |
| rs11170468 | 12 | 39430048 |  | 40862.0 | 40859.0 | 40859.2 | 40863.4 | 40862.5 | 40860.4 | 40859.5 | 40859.6 | 40860.6 | 40863.9 | 40861.0 | 40860.8 | 40859.0 | 40860.4 | 40859.5 | 40860.8 |
| rs285575 | 12 | 41921665 | PDZRN4 | 40862.0 | 40859.0 | 40859.2 | 40849.4 | 40864.0 | 40846.4 | 40861.0 | 40861.2 | 40846.5 | 40851.3 | 40848.5 | 40848.3 | 40859.0 | 40846.4 | 40846.5 | 40848.3 |
| rs11611246 | 12 | 939480 | WNK1 | 40862.0 | 40859.0 | 40859.2 | 40863.7 | 40861.2 | 40860.7 | 40858.2 | 40858.3 | 40860.9 | 40862.9 | 40860.0 | 40859.8 | 40859.0 | 40860.7 | 40858.2 | 40859.8 |
| rs10773049 | 12 | 124506631 | FAM101A | 40862.0 | 40859.0 | 40859.2 | 40862.5 | 40864.0 | 40859.5 | 40861.0 | 40861.2 | 40859.7 | 40864.5 | 40861.7 | 40861.5 | 40859.0 | 40859.5 | 40859.7 | 40861.5 |
| rs11065987 | 12 | 112072424 |  | 40862.0 | 40859.0 | 40859.2 | 40852.3 | 40863.6 | 40849.2 | 40860.6 | 40860.7 | 40849.4 | 40853.8 | 40850.9 | 40850.7 | 40859.0 | 40849.2 | 40849.4 | 40850.7 |
| rs11168854 | 12 | 49485373 | DHH | 40862.0 | 40859.0 | 40859.2 | 40861.8 | 40864.0 | 40858.8 | 40861.0 | 40861.2 | 40859.0 | 40863.8 | 40861.0 | 40860.8 | 40859.0 | 40858.8 | 40859.0 | 40860.8 |
| rs11247009 | 12 | 132701184 | GALNT9 | 40862.0 | 40859.0 | 40859.2 | 40863.9 | 40864.0 | 40860.9 | 40861.0 | 40861.2 | 40861.0 | 40865.8 | 40863.0 | 40862.8 | 40859.0 | 40860.9 | 40861.0 | 40862.8 |
| rs17630235 | 12 | 112591686 | TRAFD1 | 40862.0 | 40859.0 | 40859.2 | 40853.4 | 40862.8 | 40850.2 | 40859.8 | 40859.8 | 40850.5 | 40854.1 | 40851.1 | 40851.0 | 40859.0 | 40850.2 | 40850.5 | 40851.0 |
| rs2579106 | 12 | 90628230 |  | 40862.0 | 40859.0 | 40859.2 | 40859.2 | 40863.9 | 40856.2 | 40860.9 | 40861.0 | 40856.4 | 40861.1 | 40858.2 | 40858.1 | 40859.0 | 40856.2 | 40856.4 | 40858.1 |
| rs2720298 | 12 | 50189807 |  | 40862.0 | 40859.0 | 40859.2 | 40857.1 | 40863.0 | 40854.2 | 40860.0 | 40860.3 | 40854.3 | 40858.1 | 40855.4 | 40855.2 | 40859.0 | 40854.2 | 40854.3 | 40855.2 |
| rs7138803 | 12 | 50247468 |  | 40862.0 | 40859.0 | 40859.2 | 40858.3 | 40863.3 | 40855.2 | 40860.3 | 40860.4 | 40855.4 | 40859.5 | 40856.6 | 40856.4 | 40859.0 | 40855.2 | 40855.4 | 40856.4 |
| rs9651934 | 12 | 89355709 |  | 40862.0 | 40859.0 | 40859.2 | 40861.7 | 40863.9 | 40858.7 | 40860.9 | 40861.1 | 40858.8 | 40863.6 | 40860.7 | 40860.6 | 40859.0 | 40858.7 | 40858.8 | 40860.6 |
| rs9540493 | 13 | 66205704 |  | 40862.0 | 40859.0 | 40859.2 | 40858.7 | 40861.2 | 40855.6 | 40858.2 | 40858.2 | 40855.9 | 40857.8 | 40854.9 | 40854.7 | 40859.0 | 40855.6 | 40855.9 | 40854.7 |
| rs1441264 | 13 | 79580919 |  | 40862.0 | 40859.0 | 40859.2 | 40863.7 | 40863.4 | 40860.7 | 40860.4 | 40860.6 | 40860.8 | 40865.1 | 40862.2 | 40862.1 | 40859.0 | 40860.7 | 40860.4 | 40862.1 |
| rs1164586 | 13 | 97049141 | HS6ST3 | 40862.0 | 40859.0 | 40859.2 | 40860.8 | 40863.7 | 40857.7 | 40860.7 | 40860.8 | 40857.9 | 40862.5 | 40859.6 | 40859.4 | 40859.0 | 40857.7 | 40857.9 | 40859.4 |
| rs12429545 | 13 | 54102206 |  | 40862.0 | 40859.0 | 40859.2 | 40855.0 | 40862.1 | 40852.1 | 40859.1 | 40859.4 | 40852.1 | 40855.1 | 40852.4 | 40852.2 | 40859.0 | 40852.1 | 40852.1 | 40852.2 |
| rs7332115 | 13 | 33147548 |  | 40862.0 | 40859.0 | 40859.2 | 40863.9 | 40864.0 | 40860.9 | 40861.0 | 40861.1 | 40861.0 | 40865.8 | 40863.0 | 40862.8 | 40859.0 | 40860.9 | 40861.0 | 40862.8 |
| rs9507983 | 13 | 28620036 | FLT3 | 40862.0 | 40859.0 | 40859.2 | 40863.9 | 40863.1 | 40860.9 | 40860.1 | 40860.2 | 40861.0 | 40865.0 | 40862.1 | 40862.0 | 40859.0 | 40860.9 | 40860.1 | 40862.0 |
| rs9563576 | 13 | 58670147 |  | 40862.0 | 40859.0 | 40859.2 | 40861.0 | 40862.5 | 40858.1 | 40859.5 | 40859.7 | 40858.2 | 40861.4 | 40858.7 | 40858.5 | 40859.0 | 40858.1 | 40858.2 | 40858.5 |
| rs12016871 | 13 | 28017782 | MTIF3 | 40862.0 | 40859.0 | 40859.2 | 40858.5 | 40859.5 | 40855.7 | 40856.5 | 40856.8 | 40855.7 | 40856.0 | 40853.3 | 40853.1 | 40859.0 | 40855.7 | 40855.7 | 40853.1 |
| rs17522122 | 14 | 33302882 | AKAP6 | 40862.0 | 40859.0 | 40859.2 | 40856.7 | 40864.0 | 40853.6 | 40861.0 | 40861.1 | 40853.8 | 40858.6 | 40855.7 | 40855.6 | 40859.0 | 40853.6 | 40853.8 | 40855.6 |
| rs12894211 | 14 | 69794551 | GALNT16 | 40862.0 | 40859.0 | 40859.2 | 40864.0 | 40863.8 | 40861.0 | 40860.8 | 40861.0 | 40861.2 | 40865.8 | 40863.0 | 40862.8 | 40859.0 | 40861.0 | 40860.8 | 40862.8 |
| rs3783890 | 14 | 93790276 | BTBD7 | 40862.0 | 40859.0 | 40859.2 | 40863.9 | 40864.0 | 40860.9 | 40860.9 | 40861.1 | 40861.0 | 40865.8 | 40862.9 | 40862.8 | 40859.0 | 40860.9 | 40860.9 | 40862.8 |
| rs10132280 | 14 | 25928179 |  | 40862.0 | 40859.0 | 40859.2 | 40860.8 | 40863.8 | 40857.8 | 40860.8 | 40860.9 | 40858.0 | 40862.6 | 40859.7 | 40859.5 | 40859.0 | 40857.8 | 40858.0 | 40859.5 |
| rs12885454 | 14 | 29736838 | RP11-562L8.1 | 40862.0 | 40859.0 | 40859.2 | 40861.7 | 40864.0 | 40858.7 | 40861.0 | 40861.1 | 40858.8 | 40863.6 | 40860.8 | 40860.6 | 40859.0 | 40858.7 | 40858.8 | 40860.6 |
| rs709400 | 14 | 104149475 | RP11-73M18.2 | 40862.0 | 40859.0 | 40859.2 | 40864.0 | 40863.1 | 40861.0 | 40860.1 | 40860.2 | 40861.2 | 40865.1 | 40862.2 | 40862.1 | 40859.0 | 40861.0 | 40860.1 | 40862.1 |
| rs7143963 | 14 | 103304425 | TRAF3 | 40862.0 | 40859.0 | 40859.2 | 40863.9 | 40863.7 | 40860.9 | 40860.7 | 40860.8 | 40861.0 | 40865.5 | 40862.7 | 40862.5 | 40859.0 | 40860.9 | 40860.7 | 40862.5 |
| rs7144011 | 14 | 79940383 | NRXN3 | 40862.0 | 40859.0 | 40859.2 | 40862.7 | 40863.0 | 40859.8 | 40860.0 | 40860.2 | 40859.9 | 40863.7 | 40860.9 | 40860.7 | 40859.0 | 40859.8 | 40859.9 | 40860.7 |
| rs8016859 | 14 | 30484722 | PRKD1 | 40862.0 | 40859.0 | 40859.2 | 40850.3 | 40863.2 | 40847.2 | 40860.2 | 40860.3 | 40847.5 | 40851.5 | 40848.5 | 40848.4 | 40859.0 | 40847.2 | 40847.5 | 40848.4 |
| rs7164727 | 15 | 73093991 | ADPGK-AS1 | 40862.0 | 40859.0 | 40859.2 | 40861.4 | 40863.6 | 40858.5 | 40860.6 | 40860.8 | 40858.6 | 40863.0 | 40860.2 | 40860.0 | 40859.0 | 40858.5 | 40858.6 | 40860.0 |
| rs12593036 | 15 | 81058652 |  | 40862.0 | 40859.0 | 40859.2 | 40864.0 | 40863.9 | 40861.0 | 40860.9 | 40861.1 | 40861.2 | 40865.9 | 40863.1 | 40862.9 | 40859.0 | 40861.0 | 40860.9 | 40862.9 |
| rs13329567 | 15 | 68104367 | MAP2K5 | 40862.0 | 40859.0 | 40859.2 | 40860.2 | 40863.1 | 40857.1 | 40860.1 | 40860.2 | 40857.3 | 40861.2 | 40858.3 | 40858.1 | 40859.0 | 40857.1 | 40857.3 | 40858.1 |
| rs1439620 | 15 | 93429646 | LINC01578 | 40862.0 | 40859.0 | 40859.2 | 40863.5 | 40863.1 | 40860.4 | 40860.1 | 40860.2 | 40860.6 | 40864.5 | 40861.6 | 40861.5 | 40859.0 | 40860.4 | 40860.1 | 40861.5 |
| rs3736485 | 15 | 51748610 | DMXL2 | 40862.0 | 40859.0 | 40859.2 | 40863.2 | 40864.0 | 40860.2 | 40861.0 | 40861.2 | 40860.4 | 40865.2 | 40862.4 | 40862.2 | 40859.0 | 40860.2 | 40860.4 | 40862.2 |
| rs4984406 | 15 | 95268494 |  | 40862.0 | 40859.0 | 40859.2 | 40858.7 | 40864.0 | 40855.6 | 40861.0 | 40861.1 | 40855.8 | 40860.6 | 40857.8 | 40857.6 | 40859.0 | 40855.6 | 40855.8 | 40857.6 |
| rs12446632 | 16 | 19935389 |  | 40862.0 | 40859.0 | 40859.2 | 40861.5 | 40864.0 | 40858.5 | 40861.0 | 40861.1 | 40858.6 | 40863.4 | 40860.6 | 40860.4 | 40859.0 | 40858.5 | 40858.6 | 40860.4 |
| rs1421085 | 16 | 53800954 | FTO | 40862.0 | 40859.0 | 40859.2 | 40829.4 | 40861.2 | 40826.1 | 40858.2 | 40858.0 | 40826.6 | 40828.6 | 40825.3 | 40825.2 | 40859.0 | 40826.1 | 40826.6 | 40825.2 |
| rs2307022 | 16 | 68381978 | PRMT7 | 40862.0 | 40859.0 | 40859.2 | 40864.0 | 40862.3 | 40861.0 | 40859.3 | 40859.5 | 40861.2 | 40864.3 | 40861.4 | 40861.3 | 40859.0 | 40861.0 | 40859.3 | 40861.3 |
| rs889398 | 16 | 69556715 |  | 40862.0 | 40859.0 | 40859.2 | 40859.3 | 40863.5 | 40856.4 | 40860.5 | 40860.7 | 40856.5 | 40860.8 | 40858.0 | 40857.8 | 40859.0 | 40856.4 | 40856.5 | 40857.8 |
| rs2650492 | 16 | 28333411 | SBK1 | 40862.0 | 40859.0 | 40859.2 | 40864.0 | 40864.0 | 40861.0 | 40861.0 | 40861.1 | 40861.2 | 40866.0 | 40863.1 | 40862.9 | 40859.0 | 40861.0 | 40861.0 | 40862.9 |
| rs12448257 | 16 | 3599655 | NLRC3 | 40862.0 | 40859.0 | 40859.2 | 40862.6 | 40861.8 | 40859.5 | 40858.8 | 40858.9 | 40859.8 | 40862.4 | 40859.5 | 40859.3 | 40859.0 | 40859.5 | 40858.8 | 40859.3 |
| rs2080454 | 16 | 49062590 |  | 40862.0 | 40859.0 | 40859.2 | 40861.9 | 40861.7 | 40859.0 | 40858.7 | 40858.9 | 40859.1 | 40861.6 | 40858.8 | 40858.6 | 40859.0 | 40859.0 | 40858.7 | 40858.6 |
| rs2885415 | 16 | 395396 | AXIN1 | 40862.0 | 40859.0 | 40859.2 | 40863.0 | 40862.6 | 40860.0 | 40859.6 | 40859.8 | 40860.1 | 40863.5 | 40860.7 | 40860.5 | 40859.0 | 40860.0 | 40859.6 | 40860.5 |
| rs3888190 | 16 | 28889486 | ATP2A1 | 40862.0 | 40859.0 | 40859.2 | 40859.9 | 40863.6 | 40856.9 | 40860.6 | 40860.7 | 40857.1 | 40861.5 | 40858.6 | 40858.4 | 40859.0 | 40856.9 | 40857.1 | 40858.4 |
| rs4787491 | 16 | 30015337 | INO80E | 40862.0 | 40859.0 | 40859.2 | 40864.0 | 40864.0 | 40861.0 | 40861.0 | 40861.2 | 40861.1 | 40866.0 | 40863.1 | 40863.0 | 40859.0 | 40861.0 | 40861.0 | 40863.0 |
| rs4985155 | 16 | 15129459 | PDXDC1 | 40862.0 | 40859.0 | 40859.2 | 40863.9 | 40859.6 | 40860.9 | 40856.6 | 40856.8 | 40861.1 | 40861.5 | 40858.7 | 40858.5 | 40859.0 | 40859.6 | 40856.6 | 40858.5 |
| rs7189501 | 16 | 6175429 | RBFOX1 | 40862.0 | 40859.0 | 40859.2 | 40861.9 | 40863.7 | 40859.0 | 40860.7 | 40860.9 | 40859.1 | 40863.6 | 40860.8 | 40860.6 | 40859.0 | 40859.0 | 40859.1 | 40860.6 |
| rs749767 | 16 | 31124407 | KAT8 | 40862.0 | 40859.0 | 40859.2 | 40862.3 | 40857.4 | 40859.2 | 40854.4 | 40854.4 | 40859.4 | 40857.7 | 40854.7 | 40854.5 | 40859.0 | 40857.4 | 40854.4 | 40854.5 |
| rs12150665 | 17 | 34914787 |  | 40862.0 | 40859.0 | 40859.2 | 40862.5 | 40864.0 | 40859.5 | 40861.0 | 40861.2 | 40859.7 | 40864.5 | 40861.7 | 40861.5 | 40859.0 | 40859.5 | 40859.7 | 40861.5 |
| rs7223966 | 17 | 61893398 | DDX42 | 40862.0 | 40859.0 | 40859.2 | 40863.7 | 40862.8 | 40860.8 | 40859.8 | 40859.9 | 40860.9 | 40864.5 | 40861.6 | 40861.5 | 40859.0 | 40860.8 | 40859.8 | 40861.5 |
| rs1000940 | 17 | 5283252 | RABEP1 | 40862.0 | 40859.0 | 40859.2 | 40862.7 | 40863.6 | 40859.7 | 40860.6 | 40860.8 | 40859.8 | 40864.2 | 40861.4 | 40861.2 | 40859.0 | 40859.7 | 40859.8 | 40861.2 |
| rs12940622 | 17 | 78615571 | RPTOR | 40862.0 | 40859.0 | 40859.2 | 40846.7 | 40861.7 | 40843.9 | 40858.7 | 40859.1 | 40843.8 | 40846.3 | 40843.7 | 40843.5 | 40859.0 | 40843.9 | 40843.8 | 40843.5 |
| rs16941731 | 17 | 45317022 |  | 40862.0 | 40859.0 | 40859.2 | 40864.0 | 40863.3 | 40861.0 | 40860.3 | 40860.4 | 40861.2 | 40865.2 | 40862.4 | 40862.2 | 40859.0 | 40861.0 | 40860.3 | 40862.2 |
| rs4986044 | 17 | 21261560 |  | 40862.0 | 40859.0 | 40859.2 | 40863.5 | 40862.7 | 40860.6 | 40859.7 | 40859.9 | 40860.7 | 40864.2 | 40861.3 | 40861.2 | 40859.0 | 40860.6 | 40859.7 | 40861.2 |
| rs6504108 | 17 | 46292923 | SKAP1 | 40862.0 | 40859.0 | 40859.2 | 40864.0 | 40863.6 | 40861.0 | 40860.6 | 40860.7 | 40861.2 | 40865.6 | 40862.7 | 40862.5 | 40859.0 | 40861.0 | 40860.6 | 40862.5 |
| rs9914578 | 17 | 2005136 | SMG6 | 40862.0 | 40859.0 | 40859.2 | 40862.1 | 40861.9 | 40859.1 | 40858.9 | 40859.1 | 40859.2 | 40861.9 | 40859.1 | 40859.0 | 40859.0 | 40859.1 | 40858.9 | 40859.0 |
| rs7226371 | 18 | 1850771 |  | 40862.0 | 40859.0 | 40859.2 | 40861.4 | 40862.3 | 40858.3 | 40859.3 | 40859.4 | 40858.6 | 40861.7 | 40858.7 | 40858.6 | 40859.0 | 40858.3 | 40858.6 | 40858.6 |
| rs7243357 | 18 | 56883319 | GRP | 40862.0 | 40859.0 | 40859.2 | 40860.8 | 40864.0 | 40857.8 | 40861.0 | 40861.2 | 40858.0 | 40862.8 | 40859.9 | 40859.8 | 40859.0 | 40857.8 | 40858.0 | 40859.8 |
| rs12454712 | 18 | 60845884 | BCL2 | 40862.0 | 40859.0 | 40859.2 | 40860.6 | 40863.8 | 40857.5 | 40860.8 | 40860.9 | 40857.7 | 40862.3 | 40859.4 | 40859.3 | 40859.0 | 40857.5 | 40857.7 | 40859.3 |
| rs12961799 | 18 | 12904399 | PTPN2 | 40862.0 | 40859.0 | 40859.2 | 40863.5 | 40863.7 | 40860.5 | 40860.7 | 40860.9 | 40860.7 | 40865.2 | 40862.4 | 40862.2 | 40859.0 | 40860.5 | 40860.7 | 40862.2 |
| rs1788820 | 18 | 21101944 | C18orf8 | 40862.0 | 40859.0 | 40859.2 | 40857.5 | 40862.9 | 40854.6 | 40859.9 | 40860.2 | 40854.6 | 40858.4 | 40855.6 | 40855.5 | 40859.0 | 40854.6 | 40854.6 | 40855.5 |
| rs555267 | 18 | 40992698 |  | 40862.0 | 40859.0 | 40859.2 | 40863.7 | 40863.8 | 40860.7 | 40860.8 | 40861.0 | 40860.8 | 40865.4 | 40862.6 | 40862.4 | 40859.0 | 40860.7 | 40860.8 | 40862.4 |
| rs6567160 | 18 | 57829135 | RNU4-17P | 40862.0 | 40859.0 | 40859.2 | 40850.9 | 40860.0 | 40847.6 | 40857.0 | 40856.9 | 40848.0 | 40848.8 | 40845.8 | 40845.6 | 40859.0 | 40847.6 | 40848.0 | 40845.6 |
| rs7239883 | 18 | 40147671 | LINC00907 | 40862.0 | 40859.0 | 40859.2 | 40861.2 | 40862.0 | 40858.1 | 40859.0 | 40859.0 | 40858.3 | 40861.1 | 40858.2 | 40858.0 | 40859.0 | 40858.1 | 40858.3 | 40858.0 |
| rs11672550 | 19 | 1937193 | CSNK1G2 | 40862.0 | 40859.0 | 40859.2 | 40864.0 | 40863.9 | 40860.9 | 40860.9 | 40861.0 | 40861.1 | 40865.8 | 40862.9 | 40862.8 | 40859.0 | 40860.9 | 40860.9 | 40862.8 |
| rs2304130 | 19 | 19789528 | ZNF101 | 40862.0 | 40859.0 | 40859.2 | 40863.5 | 40863.3 | 40860.5 | 40860.3 | 40860.4 | 40860.6 | 40864.7 | 40861.8 | 40861.7 | 40859.0 | 40860.5 | 40860.3 | 40861.7 |
| rs17513613 | 19 | 30286822 |  | 40862.0 | 40859.0 | 40859.2 | 40862.1 | 40863.6 | 40859.1 | 40860.6 | 40860.7 | 40859.2 | 40863.6 | 40860.8 | 40860.6 | 40859.0 | 40859.1 | 40859.2 | 40860.6 |
| rs33439 | 19 | 30945171 | ZNF536 | 40862.0 | 40859.0 | 40859.2 | 40863.8 | 40859.3 | 40860.8 | 40856.3 | 40856.5 | 40861.0 | 40861.1 | 40858.3 | 40858.1 | 40859.0 | 40859.3 | 40856.3 | 40858.1 |
| rs2075650 | 19 | 45395619 | TOMM40 | 40862.0 | 40859.0 | 40859.2 | 40864.0 | 40860.3 | 40861.0 | 40857.3 | 40857.4 | 40861.2 | 40862.3 | 40859.4 | 40859.3 | 40859.0 | 40860.3 | 40857.3 | 40859.3 |
| rs3810291 | 19 | 47569003 | ZC3H4 | 40862.0 | 40859.0 | 40859.2 | 40863.5 | 40864.0 | 40860.5 | 40861.0 | 40861.2 | 40860.6 | 40865.5 | 40862.6 | 40862.5 | 40859.0 | 40860.5 | 40860.6 | 40862.5 |
| rs11672660 | 19 | 46180184 | GIPR | 40862.0 | 40859.0 | 40859.2 | 40850.3 | 40863.9 | 40847.3 | 40860.9 | 40861.0 | 40847.5 | 40852.2 | 40849.3 | 40849.2 | 40859.0 | 40847.3 | 40847.5 | 40849.2 |
| rs14810 | 19 | 34304903 | KCTD15 | 40862.0 | 40859.0 | 40859.2 | 40863.0 | 40863.8 | 40860.0 | 40860.8 | 40861.0 | 40860.2 | 40864.8 | 40862.0 | 40861.8 | 40859.0 | 40860.0 | 40860.2 | 40861.8 |
| rs17724992 | 19 | 18454825 | PGPEP1 | 40862.0 | 40859.0 | 40859.2 | 40859.6 | 40864.0 | 40856.6 | 40861.0 | 40861.2 | 40856.7 | 40861.6 | 40858.7 | 40858.6 | 40859.0 | 40856.6 | 40856.7 | 40858.6 |
| rs8123881 | 20 | 15819495 | MACROD2 | 40862.0 | 40859.0 | 40859.2 | 40863.8 | 40863.7 | 40860.8 | 40860.7 | 40860.8 | 40860.9 | 40865.4 | 40862.6 | 40862.4 | 40859.0 | 40860.8 | 40860.7 | 40862.4 |
| rs1884897 | 20 | 6612832 |  | 40862.0 | 40859.0 | 40859.2 | 40863.7 | 40864.0 | 40860.7 | 40861.0 | 40861.2 | 40860.8 | 40865.7 | 40862.8 | 40862.7 | 40859.0 | 40860.7 | 40860.8 | 40862.7 |
| rs2236176 | 20 | 21081488 | LINC00237 | 40862.0 | 40859.0 | 40859.2 | 40859.5 | 40863.8 | 40856.4 | 40860.8 | 40860.9 | 40856.6 | 40861.2 | 40858.4 | 40858.2 | 40859.0 | 40856.4 | 40856.6 | 40858.2 |
| rs4809401 | 20 | 62737568 | NPBWR2 | 40862.0 | 40859.0 | 40859.2 | 40859.4 | 40862.7 | 40856.3 | 40859.6 | 40859.7 | 40856.6 | 40860.0 | 40857.1 | 40856.9 | 40859.0 | 40856.3 | 40856.6 | 40856.9 |
| rs6010784 | 20 | 61540319 | DIDO1 | 40862.0 | 40859.0 | 40859.2 | 40864.0 | 40863.7 | 40861.0 | 40860.7 | 40860.9 | 40861.1 | 40865.6 | 40862.8 | 40862.6 | 40859.0 | 40861.0 | 40860.7 | 40862.6 |
| rs6091540 | 20 | 51087862 | LINC01524 | 40862.0 | 40859.0 | 40859.2 | 40855.0 | 40861.9 | 40851.8 | 40858.9 | 40858.9 | 40852.1 | 40854.8 | 40851.8 | 40851.6 | 40859.0 | 40851.8 | 40852.1 | 40851.6 |
| rs2836754 | 21 | 40291740 | AF064858.6 | 40862.0 | 40859.0 | 40859.2 | 40863.1 | 40860.8 | 40860.1 | 40857.8 | 40858.0 | 40860.2 | 40861.8 | 40859.0 | 40858.8 | 40859.0 | 40860.1 | 40857.8 | 40858.8 |
| rs427943 | 21 | 46570896 | ADARB1 | 40862.0 | 40859.0 | 40859.2 | 40862.4 | 40861.5 | 40859.4 | 40858.5 | 40858.7 | 40859.5 | 40861.8 | 40859.1 | 40858.9 | 40859.0 | 40859.4 | 40858.5 | 40858.9 |
| rs914187 | 21 | 42619749 | BACE2 | 40862.0 | 40859.0 | 40859.2 | 40863.2 | 40864.0 | 40860.2 | 40861.0 | 40861.2 | 40860.3 | 40865.2 | 40862.3 | 40862.2 | 40859.0 | 40860.2 | 40860.3 | 40862.2 |
| rs134871 | 22 | 42652716 | TCF20 | 40862.0 | 40859.0 | 40859.2 | 40861.2 | 40863.0 | 40858.2 | 40860.0 | 40860.1 | 40858.4 | 40862.2 | 40859.3 | 40859.2 | 40859.0 | 40858.2 | 40858.4 | 40859.2 |
| rs4820408 | 22 | 40604945 | TNRC6B | 40862.0 | 40859.0 | 40859.2 | 40858.6 | 40864.0 | 40855.6 | 40861.0 | 40861.2 | 40855.8 | 40860.6 | 40857.8 | 40857.6 | 40859.0 | 40855.6 | 40855.8 | 40857.6 |
| **BMI/Pack-years** | | | | | | | | | | | | | | | | | | | |
| SNP | chrom | position | gene_name | DAG1 | DAG2 | DAG3 | DAG4 | DAG5 | DAG6 | DAG7 | DAG8 | DAG9 | DAG10 | DAG11 | DAG12 | C1 | C2 | C3 | C4 |
| rs7550711 | 1 | 110082886 | GPR61 | 45436.0 | 45428.4 | 45428.4 | 45435.0 | 45434.1 | 45427.5 | 45426.4 | 45426.6 | 45427.3 | 45433.1 | 45425.6 | 45425.6 | 45428.4 | 45427.5 | 45426.4 | 45425.6 |
| rs1546924 | 1 | 112273485 | FAM212B | 45436.0 | 45428.4 | 45428.4 | 45437.9 | 45436.3 | 45430.2 | 45428.7 | 45428.7 | 45430.3 | 45438.2 | 45430.6 | 45430.6 | 45428.4 | 45430.2 | 45428.7 | 45430.6 |
| rs4357530 | 1 | 151103153 | SEMA6C | 45436.0 | 45428.4 | 45428.4 | 45437.7 | 45438.0 | 45430.0 | 45430.4 | 45430.4 | 45430.0 | 45439.7 | 45432.0 | 45432.0 | 45428.4 | 45430.0 | 45430.0 | 45432.0 |
| rs4568876 | 1 | 32152518 | COL16A1 | 45436.0 | 45428.4 | 45428.4 | 45434.1 | 45438.0 | 45426.5 | 45430.4 | 45430.4 | 45426.5 | 45436.1 | 45428.5 | 45428.5 | 45428.4 | 45426.5 | 45426.5 | 45428.5 |
| rs4061073 | 1 | 54696743 | SSBP3 | 45436.0 | 45428.4 | 45428.4 | 45437.6 | 45437.2 | 45430.0 | 45429.5 | 45429.5 | 45429.9 | 45438.7 | 45431.1 | 45431.1 | 45428.4 | 45430.0 | 45429.5 | 45431.1 |
| rs2481665 | 1 | 62594677 | INADL | 45436.0 | 45428.4 | 45428.4 | 45437.8 | 45436.7 | 45430.2 | 45429.1 | 45429.1 | 45430.1 | 45438.5 | 45430.8 | 45430.8 | 45428.4 | 45430.2 | 45429.1 | 45430.8 |
| rs11165643 | 1 | 96924097 |  | 45436.0 | 45428.4 | 45428.4 | 45429.4 | 45432.1 | 45422.1 | 45424.5 | 45424.8 | 45421.7 | 45425.5 | 45418.2 | 45418.2 | 45428.4 | 45422.1 | 45421.7 | 45418.2 |
| rs10779751 | 1 | 11284336 | MTOR | 45436.0 | 45428.4 | 45428.4 | 45425.1 | 45437.7 | 45417.5 | 45430.1 | 45430.2 | 45417.4 | 45426.8 | 45419.2 | 45419.2 | 45428.4 | 45417.5 | 45417.4 | 45419.2 |
| rs10920678 | 1 | 190239907 | BRINP3 | 45436.0 | 45428.4 | 45428.4 | 45436.9 | 45435.4 | 45429.3 | 45427.8 | 45427.9 | 45429.3 | 45436.3 | 45428.7 | 45428.7 | 45428.4 | 45429.3 | 45427.8 | 45428.7 |
| rs11583200 | 1 | 50559820 | ELAVL4 | 45436.0 | 45428.4 | 45428.4 | 45431.8 | 45428.0 | 45424.5 | 45420.4 | 45420.8 | 45424.1 | 45423.8 | 45416.5 | 45416.5 | 45428.4 | 45424.5 | 45420.4 | 45416.5 |
| rs1361739 | 1 | 96289734 |  | 45436.0 | 45428.4 | 45428.4 | 45438.0 | 45438.0 | 45430.3 | 45430.3 | 45430.3 | 45430.3 | 45439.9 | 45432.3 | 45432.3 | 45428.4 | 45430.3 | 45430.3 | 45432.3 |
| rs2088518 | 1 | 77951330 | AK5 | 45436.0 | 45428.4 | 45428.4 | 45435.7 | 45435.5 | 45428.2 | 45427.9 | 45428.0 | 45428.1 | 45435.2 | 45427.7 | 45427.7 | 45428.4 | 45428.2 | 45427.9 | 45427.7 |
| rs2275426 | 1 | 46487552 | MAST2 | 45436.0 | 45428.4 | 45428.4 | 45437.4 | 45437.8 | 45429.8 | 45430.1 | 45430.1 | 45429.8 | 45439.1 | 45431.5 | 45431.5 | 45428.4 | 45429.8 | 45429.8 | 45431.5 |
| rs2820292 | 1 | 201784287 | NAV1 | 45436.0 | 45428.4 | 45428.4 | 45435.2 | 45436.6 | 45427.4 | 45429.0 | 45428.9 | 45427.5 | 45435.8 | 45428.0 | 45428.0 | 45428.4 | 45427.4 | 45427.5 | 45428.0 |
| rs284227 | 1 | 82379446 | ADGRL2 | 45436.0 | 45428.4 | 45428.4 | 45431.5 | 45436.8 | 45423.7 | 45429.2 | 45429.1 | 45423.9 | 45432.3 | 45424.5 | 45424.5 | 45428.4 | 45423.7 | 45423.9 | 45424.5 |
| rs4130548 | 1 | 78463868 | DNAJB4 | 45436.0 | 45428.4 | 45428.4 | 45437.1 | 45433.1 | 45429.4 | 45425.5 | 45425.4 | 45429.5 | 45434.2 | 45426.5 | 45426.5 | 45428.4 | 45429.4 | 45425.4 | 45426.5 |
| rs543874 | 1 | 177889480 | SEC16B | 45436.0 | 45428.4 | 45428.4 | 45427.7 | 45437.9 | 45420.1 | 45430.2 | 45430.3 | 45420.0 | 45429.5 | 45421.9 | 45421.9 | 45428.4 | 45420.1 | 45420.0 | 45421.9 |
| rs657452 | 1 | 49589847 | AGBL4 | 45436.0 | 45428.4 | 45428.4 | 45437.3 | 45432.6 | 45429.8 | 45425.0 | 45425.1 | 45429.7 | 45433.9 | 45426.3 | 45426.3 | 45428.4 | 45429.8 | 45425.0 | 45426.3 |
| rs7531118 | 1 | 72837239 |  | 45436.0 | 45428.4 | 45428.4 | 45419.4 | 45437.8 | 45411.8 | 45430.2 | 45430.2 | 45411.7 | 45421.2 | 45413.6 | 45413.6 | 45428.4 | 45411.8 | 45411.7 | 45413.6 |
| rs7551507 | 1 | 74995225 | TNNI3K | 45436.0 | 45428.4 | 45428.4 | 45432.2 | 45436.9 | 45424.7 | 45429.2 | 45429.3 | 45424.6 | 45433.0 | 45425.5 | 45425.5 | 45428.4 | 45424.7 | 45424.6 | 45425.5 |
| rs9660180 | 1 | 1723031 | GNB1 | 45436.0 | 45428.4 | 45428.4 | 45438.0 | 45438.0 | 45430.3 | 45430.3 | 45430.3 | 45430.3 | 45439.9 | 45432.3 | 45432.3 | 45428.4 | 45430.3 | 45430.3 | 45432.3 |
| rs977747 | 1 | 47684677 | TAL1 | 45436.0 | 45428.4 | 45428.4 | 45433.6 | 45437.6 | 45426.0 | 45430.0 | 45430.0 | 45426.0 | 45435.2 | 45427.6 | 45427.6 | 45428.4 | 45426.0 | 45426.0 | 45427.6 |
| rs995258 | 1 | 97431052 |  | 45436.0 | 45428.4 | 45428.4 | 45435.9 | 45437.6 | 45428.3 | 45429.9 | 45430.0 | 45428.2 | 45437.4 | 45429.8 | 45429.8 | 45428.4 | 45428.3 | 45428.2 | 45429.8 |
| rs17406900 | 2 | 203784202 | CARF | 45436.0 | 45428.4 | 45428.4 | 45438.0 | 45437.4 | 45430.4 | 45429.7 | 45429.7 | 45430.4 | 45439.4 | 45431.7 | 45431.7 | 45428.4 | 45430.4 | 45429.7 | 45431.7 |
| rs715 | 2 | 211543055 | CPS1 | 45436.0 | 45428.4 | 45428.4 | 45432.6 | 45434.2 | 45425.2 | 45426.5 | 45426.7 | 45425.0 | 45430.8 | 45423.3 | 45423.3 | 45428.4 | 45425.2 | 45425.0 | 45423.3 |
| rs10182181 | 2 | 25150296 |  | 45436.0 | 45428.4 | 45428.4 | 45429.4 | 45437.6 | 45421.8 | 45429.9 | 45430.0 | 45421.7 | 45430.9 | 45423.4 | 45423.4 | 45428.4 | 45421.8 | 45421.7 | 45423.4 |
| rs1979755 | 2 | 42708405 | KCNG3 | 45436.0 | 45428.4 | 45428.4 | 45435.3 | 45437.9 | 45427.6 | 45430.2 | 45430.2 | 45427.7 | 45437.1 | 45429.5 | 45429.5 | 45428.4 | 45427.6 | 45427.7 | 45429.5 |
| rs1016287 | 2 | 59305625 |  | 45436.0 | 45428.4 | 45428.4 | 45436.3 | 45437.9 | 45428.7 | 45430.3 | 45430.3 | 45428.6 | 45438.2 | 45430.6 | 45430.6 | 45428.4 | 45428.7 | 45428.6 | 45430.6 |
| rs10166736 | 2 | 227900419 | COL4A4 | 45436.0 | 45428.4 | 45428.4 | 45438.0 | 45435.1 | 45430.4 | 45427.5 | 45427.5 | 45430.3 | 45437.1 | 45429.5 | 45429.5 | 45428.4 | 45430.4 | 45427.5 | 45429.5 |
| rs10929925 | 2 | 6155557 |  | 45436.0 | 45428.4 | 45428.4 | 45436.7 | 45435.2 | 45429.0 | 45427.5 | 45427.4 | 45429.1 | 45435.8 | 45428.1 | 45428.1 | 45428.4 | 45429.0 | 45427.4 | 45428.1 |
| rs11126666 | 2 | 26928811 | KCNK3 | 45436.0 | 45428.4 | 45428.4 | 45432.4 | 45437.7 | 45424.8 | 45430.0 | 45430.1 | 45424.7 | 45434.0 | 45426.4 | 45426.4 | 45428.4 | 45424.8 | 45424.7 | 45426.4 |
| rs11677911 | 2 | 237905331 |  | 45436.0 | 45428.4 | 45428.4 | 45437.6 | 45437.3 | 45429.9 | 45429.6 | 45429.6 | 45429.9 | 45438.8 | 45431.2 | 45431.2 | 45428.4 | 45429.9 | 45429.6 | 45431.2 |
| rs12622013 | 2 | 79501362 | CTNNA2 | 45436.0 | 45428.4 | 45428.4 | 45437.7 | 45436.6 | 45430.0 | 45429.0 | 45429.0 | 45430.0 | 45438.3 | 45430.6 | 45430.6 | 45428.4 | 45430.0 | 45429.0 | 45430.6 |
| rs13021737 | 2 | 632348 |  | 45436.0 | 45428.4 | 45428.4 | 45406.5 | 45430.0 | 45399.6 | 45422.3 | 45423.1 | 45398.8 | 45400.5 | 45393.5 | 45393.5 | 45428.4 | 45399.6 | 45398.8 | 45393.5 |
| rs13417156 | 2 | 62848319 | AC092155.4 | 45436.0 | 45428.4 | 45428.4 | 45437.8 | 45438.0 | 45430.1 | 45430.4 | 45430.4 | 45430.1 | 45439.8 | 45432.1 | 45432.1 | 45428.4 | 45430.1 | 45430.1 | 45432.1 |
| rs1460676 | 2 | 164567689 | FIGN | 45436.0 | 45428.4 | 45428.4 | 45438.0 | 45438.0 | 45430.3 | 45430.4 | 45430.4 | 45430.3 | 45440.0 | 45432.3 | 45432.3 | 45428.4 | 45430.3 | 45430.3 | 45432.3 |
| rs1528435 | 2 | 181550962 | AC009478.1 | 45436.0 | 45428.4 | 45428.4 | 45430.7 | 45427.7 | 45423.4 | 45420.0 | 45420.4 | 45423.0 | 45422.3 | 45415.1 | 45415.1 | 45428.4 | 45423.4 | 45420.0 | 45415.1 |
| rs1554622 | 2 | 219606218 | TTLL4 | 45436.0 | 45428.4 | 45428.4 | 45437.4 | 45437.5 | 45429.8 | 45429.8 | 45429.9 | 45429.8 | 45438.9 | 45431.2 | 45431.2 | 45428.4 | 45429.8 | 45429.8 | 45431.2 |
| rs1561277 | 2 | 136092061 | ZRANB3 | 45436.0 | 45428.4 | 45428.4 | 45438.0 | 45435.5 | 45430.4 | 45427.9 | 45427.8 | 45430.4 | 45437.5 | 45429.8 | 45429.8 | 45428.4 | 45430.4 | 45427.8 | 45429.8 |
| rs17203016 | 2 | 208255518 | AC007879.5 | 45436.0 | 45428.4 | 45428.4 | 45438.0 | 45437.6 | 45430.3 | 45430.0 | 45430.0 | 45430.3 | 45439.6 | 45431.9 | 45431.9 | 45428.4 | 45430.3 | 45430.0 | 45431.9 |
| rs2890652 | 2 | 142959931 |  | 45436.0 | 45428.4 | 45428.4 | 45437.3 | 45437.9 | 45429.6 | 45430.3 | 45430.3 | 45429.7 | 45439.2 | 45431.6 | 45431.6 | 45428.4 | 45429.6 | 45429.7 | 45431.6 |
| rs4372836 | 2 | 28973883 | PPP1CB | 45436.0 | 45428.4 | 45428.4 | 45438.0 | 45437.9 | 45430.4 | 45430.3 | 45430.3 | 45430.4 | 45439.9 | 45432.3 | 45432.3 | 45428.4 | 45430.4 | 45430.3 | 45432.3 |
| rs4667682 | 2 | 172127920 |  | 45436.0 | 45428.4 | 45428.4 | 45436.9 | 45438.0 | 45429.3 | 45430.3 | 45430.3 | 45429.3 | 45438.9 | 45431.2 | 45431.2 | 45428.4 | 45429.3 | 45429.3 | 45431.2 |
| rs4988235 | 2 | 136608646 | MCM6 | 45436.0 | 45428.4 | 45428.4 | 45437.9 | 45437.0 | 45430.2 | 45429.3 | 45429.3 | 45430.2 | 45438.8 | 45431.1 | 45431.1 | 45428.4 | 45430.2 | 45429.3 | 45431.1 |
| rs6705646 | 2 | 165567695 | COBLL1 | 45436.0 | 45428.4 | 45428.4 | 45437.7 | 45437.0 | 45430.1 | 45429.4 | 45429.4 | 45430.1 | 45438.7 | 45431.1 | 45431.1 | 45428.4 | 45430.1 | 45429.4 | 45431.1 |
| rs6713510 | 2 | 227034499 |  | 45436.0 | 45428.4 | 45428.4 | 45432.9 | 45438.0 | 45425.3 | 45430.4 | 45430.4 | 45425.3 | 45434.9 | 45427.3 | 45427.3 | 45428.4 | 45425.3 | 45425.3 | 45427.3 |
| rs7599312 | 2 | 213413231 |  | 45436.0 | 45428.4 | 45428.4 | 45438.0 | 45437.7 | 45430.4 | 45430.0 | 45430.0 | 45430.4 | 45439.7 | 45432.0 | 45432.0 | 45428.4 | 45430.4 | 45430.0 | 45432.0 |
| rs929641 | 2 | 58792377 | LINC01122 | 45436.0 | 45428.4 | 45428.4 | 45429.6 | 45437.3 | 45422.1 | 45429.6 | 45429.8 | 45421.9 | 45430.9 | 45423.3 | 45423.3 | 45428.4 | 45422.1 | 45421.9 | 45423.3 |
| rs968059 | 2 | 35404011 | AC012593.1 | 45436.0 | 45428.4 | 45428.4 | 45437.7 | 45437.1 | 45430.1 | 45429.4 | 45429.4 | 45430.0 | 45438.7 | 45431.1 | 45431.1 | 45428.4 | 45430.1 | 45429.4 | 45431.1 |
| rs972540 | 2 | 207244783 |  | 45436.0 | 45428.4 | 45428.4 | 45437.8 | 45436.4 | 45430.1 | 45428.7 | 45428.7 | 45430.1 | 45438.1 | 45430.5 | 45430.5 | 45428.4 | 45430.1 | 45428.7 | 45430.5 |
| rs7640424 | 3 | 107820063 |  | 45436.0 | 45428.4 | 45428.4 | 45437.9 | 45438.0 | 45430.3 | 45430.3 | 45430.3 | 45430.3 | 45439.9 | 45432.3 | 45432.3 | 45428.4 | 45430.3 | 45430.3 | 45432.3 |
| rs2124499 | 3 | 123093541 | ADCY5 | 45436.0 | 45428.4 | 45428.4 | 45437.8 | 45435.6 | 45430.2 | 45427.9 | 45428.0 | 45430.1 | 45437.4 | 45429.7 | 45429.7 | 45428.4 | 45430.2 | 45427.9 | 45429.7 |
| rs7620457 | 3 | 183747266 | HTR3D | 45436.0 | 45428.4 | 45428.4 | 45435.1 | 45433.1 | 45427.3 | 45425.5 | 45425.3 | 45427.5 | 45432.2 | 45424.4 | 45424.4 | 45428.4 | 45427.3 | 45425.3 | 45424.4 |
| rs2710323 | 3 | 52815905 | ITIH1 | 45436.0 | 45428.4 | 45428.4 | 45435.6 | 45437.2 | 45427.9 | 45429.6 | 45429.5 | 45427.9 | 45436.8 | 45429.1 | 45429.1 | 45428.4 | 45427.9 | 45427.9 | 45429.1 |
| rs2612012 | 3 | 53745625 | CACNA1D | 45436.0 | 45428.4 | 45428.4 | 45429.6 | 45434.6 | 45422.2 | 45426.9 | 45427.2 | 45421.9 | 45428.1 | 45420.7 | 45420.7 | 45428.4 | 45422.2 | 45421.9 | 45420.7 |
| rs2371767 | 3 | 64718258 | ADAMTS9-AS2 | 45436.0 | 45428.4 | 45428.4 | 45428.8 | 45438.0 | 45421.1 | 45430.3 | 45430.4 | 45421.1 | 45430.7 | 45423.1 | 45423.1 | 45428.4 | 45421.1 | 45421.1 | 45423.1 |
| rs13078960 | 3 | 85807590 | CADM2 | 45436.0 | 45428.4 | 45428.4 | 45437.8 | 45437.7 | 45430.2 | 45430.0 | 45430.0 | 45430.2 | 45439.5 | 45431.9 | 45431.9 | 45428.4 | 45430.2 | 45430.0 | 45431.9 |
| rs1436351 | 3 | 104617973 |  | 45436.0 | 45428.4 | 45428.4 | 45429.1 | 45437.4 | 45421.6 | 45429.7 | 45429.8 | 45421.5 | 45430.5 | 45423.0 | 45423.0 | 45428.4 | 45421.6 | 45421.5 | 45423.0 |
| rs1516725 | 3 | 185824004 | ETV5 | 45436.0 | 45428.4 | 45428.4 | 45428.6 | 45438.0 | 45420.9 | 45430.3 | 45430.4 | 45420.9 | 45430.5 | 45422.9 | 45422.9 | 45428.4 | 45420.9 | 45420.9 | 45422.9 |
| rs16851483 | 3 | 141275436 | RASA2 | 45436.0 | 45428.4 | 45428.4 | 45436.9 | 45435.3 | 45429.2 | 45427.6 | 45427.6 | 45429.2 | 45436.2 | 45428.4 | 45428.4 | 45428.4 | 45429.2 | 45427.6 | 45428.4 |
| rs2365389 | 3 | 61236462 | FHIT | 45436.0 | 45428.4 | 45428.4 | 45433.3 | 45438.0 | 45425.6 | 45430.3 | 45430.3 | 45425.6 | 45435.3 | 45427.6 | 45427.6 | 45428.4 | 45425.6 | 45425.6 | 45427.6 |
| rs3849570 | 3 | 81792112 | GBE1 | 45436.0 | 45428.4 | 45428.4 | 45436.8 | 45437.4 | 45429.1 | 45429.8 | 45429.7 | 45429.2 | 45438.2 | 45430.5 | 45430.5 | 45428.4 | 45429.1 | 45429.2 | 45430.5 |
| rs4395360 | 3 | 157318257 | PQLC2L | 45436.0 | 45428.4 | 45428.4 | 45435.0 | 45438.0 | 45427.3 | 45430.4 | 45430.4 | 45427.3 | 45437.0 | 45429.3 | 45429.3 | 45428.4 | 45427.3 | 45427.3 | 45429.3 |
| rs6804842 | 3 | 25106437 | AC133680.1 | 45436.0 | 45428.4 | 45428.4 | 45436.7 | 45438.0 | 45429.1 | 45430.4 | 45430.4 | 45429.1 | 45438.7 | 45431.1 | 45431.1 | 45428.4 | 45429.1 | 45429.1 | 45431.1 |
| rs7611238 | 3 | 195072918 | ACAP2 | 45436.0 | 45428.4 | 45428.4 | 45432.7 | 45437.2 | 45425.2 | 45429.5 | 45429.6 | 45425.1 | 45433.9 | 45426.3 | 45426.3 | 45428.4 | 45425.2 | 45425.1 | 45426.3 |
| rs7613875 | 3 | 49971514 | MON1A | 45436.0 | 45428.4 | 45428.4 | 45437.7 | 45438.0 | 45430.1 | 45430.3 | 45430.3 | 45430.1 | 45439.7 | 45432.0 | 45432.0 | 45428.4 | 45430.1 | 45430.1 | 45432.0 |
| rs7649970 | 3 | 12392272 | PPARG | 45436.0 | 45428.4 | 45428.4 | 45434.5 | 45437.7 | 45426.9 | 45430.0 | 45430.1 | 45426.9 | 45436.2 | 45428.6 | 45428.6 | 45428.4 | 45426.9 | 45426.9 | 45428.6 |
| rs876424 | 3 | 131637676 | CPNE4 | 45436.0 | 45428.4 | 45428.4 | 45434.0 | 45437.9 | 45426.4 | 45430.3 | 45430.3 | 45426.4 | 45435.9 | 45428.3 | 45428.3 | 45428.4 | 45426.4 | 45426.4 | 45428.3 |
| rs9867325 | 3 | 136618909 | NCK1 | 45436.0 | 45428.4 | 45428.4 | 45437.9 | 45436.2 | 45430.3 | 45428.6 | 45428.6 | 45430.3 | 45438.1 | 45430.5 | 45430.5 | 45428.4 | 45430.3 | 45428.6 | 45430.5 |
| rs9880211 | 3 | 136107549 | STAG1 | 45436.0 | 45428.4 | 45428.4 | 45437.6 | 45436.5 | 45429.9 | 45428.9 | 45428.9 | 45429.9 | 45438.1 | 45430.5 | 45430.5 | 45428.4 | 45429.9 | 45428.9 | 45430.5 |
| rs13107325 | 4 | 103188709 | SLC39A8 | 45436.0 | 45428.4 | 45428.4 | 45429.9 | 45437.9 | 45422.2 | 45430.3 | 45430.2 | 45422.2 | 45431.8 | 45424.1 | 45424.1 | 45428.4 | 45422.2 | 45422.2 | 45424.1 |
| rs11727676 | 4 | 145659064 | HHIP | 45436.0 | 45428.4 | 45428.4 | 45437.3 | 45438.0 | 45429.7 | 45430.3 | 45430.3 | 45429.7 | 45439.3 | 45431.6 | 45431.6 | 45428.4 | 45429.7 | 45429.7 | 45431.6 |
| rs4833079 | 4 | 38654681 |  | 45436.0 | 45428.4 | 45428.4 | 45432.4 | 45437.7 | 45424.7 | 45430.1 | 45430.0 | 45424.8 | 45434.1 | 45426.4 | 45426.4 | 45428.4 | 45424.7 | 45424.8 | 45426.4 |
| rs10009336 | 4 | 44480783 |  | 45436.0 | 45428.4 | 45428.4 | 45436.4 | 45437.9 | 45428.8 | 45430.2 | 45430.2 | 45428.8 | 45438.3 | 45430.6 | 45430.6 | 45428.4 | 45428.8 | 45428.8 | 45430.6 |
| rs17001654 | 4 | 77129568 | SCARB2 | 45436.0 | 45428.4 | 45428.4 | 45437.7 | 45435.1 | 45430.0 | 45427.4 | 45427.4 | 45430.0 | 45436.8 | 45429.1 | 45429.1 | 45428.4 | 45430.0 | 45427.4 | 45429.1 |
| rs13130484 | 4 | 45175691 |  | 45436.0 | 45428.4 | 45428.4 | 45413.6 | 45437.7 | 45406.1 | 45430.0 | 45430.2 | 45406.0 | 45415.3 | 45407.8 | 45407.8 | 45428.4 | 45406.1 | 45406.0 | 45407.8 |
| rs2391518 | 4 | 130817060 | RP11-422J15.1 | 45436.0 | 45428.4 | 45428.4 | 45437.7 | 45437.7 | 45430.0 | 45430.0 | 45430.0 | 45430.0 | 45439.3 | 45431.7 | 45431.7 | 45428.4 | 45430.0 | 45430.0 | 45431.7 |
| rs6864049 | 5 | 124330522 |  | 45436.0 | 45428.4 | 45428.4 | 45435.8 | 45436.1 | 45428.2 | 45428.5 | 45428.6 | 45428.1 | 45435.8 | 45428.3 | 45428.3 | 45428.4 | 45428.2 | 45428.1 | 45428.3 |
| rs6870983 | 5 | 87697533 | TMEM161B-AS1 | 45436.0 | 45428.4 | 45428.4 | 45429.8 | 45438.0 | 45422.2 | 45430.4 | 45430.3 | 45422.2 | 45431.8 | 45424.2 | 45424.2 | 45428.4 | 45422.2 | 45422.2 | 45424.2 |
| rs11951673 | 5 | 95861012 | CAST | 45436.0 | 45428.4 | 45428.4 | 45426.0 | 45437.9 | 45418.3 | 45430.2 | 45430.2 | 45418.4 | 45427.9 | 45420.2 | 45420.2 | 45428.4 | 45418.3 | 45418.4 | 45420.2 |
| rs150992 | 5 | 98275197 | CTD-2007H13.3 | 45436.0 | 45428.4 | 45428.4 | 45438.0 | 45438.0 | 45430.3 | 45430.3 | 45430.3 | 45430.3 | 45439.9 | 45432.3 | 45432.3 | 45428.4 | 45430.3 | 45430.3 | 45432.3 |
| rs2112347 | 5 | 75015242 | POC5 | 45436.0 | 45428.4 | 45428.4 | 45437.2 | 45436.5 | 45429.5 | 45428.8 | 45428.7 | 45429.6 | 45437.6 | 45429.9 | 45429.9 | 45428.4 | 45429.5 | 45428.7 | 45429.9 |
| rs288232 | 5 | 107419548 | FBXL17 | 45436.0 | 45428.4 | 45428.4 | 45437.6 | 45436.1 | 45429.9 | 45428.5 | 45428.4 | 45430.0 | 45437.7 | 45430.0 | 45430.0 | 45428.4 | 45429.9 | 45428.4 | 45430.0 |
| rs7715256 | 5 | 153537893 | MFAP3 | 45436.0 | 45428.4 | 45428.4 | 45426.3 | 45438.0 | 45418.6 | 45430.4 | 45430.3 | 45418.6 | 45428.3 | 45420.6 | 45420.6 | 45428.4 | 45418.6 | 45418.6 | 45420.6 |
| rs6569648 | 6 | 130349119 | L3MBTL3 | 45436.0 | 45428.4 | 45428.4 | 45437.5 | 45436.3 | 45429.9 | 45428.6 | 45428.7 | 45429.8 | 45437.7 | 45430.1 | 45430.1 | 45428.4 | 45429.9 | 45428.6 | 45430.1 |
| rs13201877 | 6 | 137675541 |  | 45436.0 | 45428.4 | 45428.4 | 45431.7 | 45436.4 | 45424.2 | 45428.7 | 45428.9 | 45424.1 | 45432.1 | 45424.6 | 45424.6 | 45428.4 | 45424.2 | 45424.1 | 45424.6 |
| rs13191362 | 6 | 163033350 | PARK2 | 45436.0 | 45428.4 | 45428.4 | 45435.6 | 45437.8 | 45427.9 | 45430.2 | 45430.1 | 45427.9 | 45437.4 | 45429.7 | 45429.7 | 45428.4 | 45427.9 | 45427.9 | 45429.7 |
| rs943466 | 6 | 33731787 |  | 45436.0 | 45428.4 | 45428.4 | 45437.7 | 45438.0 | 45430.0 | 45430.4 | 45430.4 | 45430.0 | 45439.7 | 45432.0 | 45432.0 | 45428.4 | 45430.0 | 45430.0 | 45432.0 |
| rs206936 | 6 | 34302869 | NUDT3 | 45436.0 | 45428.4 | 45428.4 | 45427.6 | 45437.3 | 45420.0 | 45429.7 | 45429.8 | 45419.9 | 45428.9 | 45421.3 | 45421.3 | 45428.4 | 45420.0 | 45419.9 | 45421.3 |
| rs1358980 | 6 | 43764551 |  | 45436.0 | 45428.4 | 45428.4 | 45437.5 | 45437.9 | 45429.8 | 45430.3 | 45430.3 | 45429.8 | 45439.4 | 45431.7 | 45431.7 | 45428.4 | 45429.8 | 45429.8 | 45431.7 |
| rs17665162 | 6 | 50275258 |  | 45436.0 | 45428.4 | 45428.4 | 45435.6 | 45437.3 | 45428.0 | 45429.6 | 45429.7 | 45428.0 | 45436.9 | 45429.3 | 45429.3 | 45428.4 | 45428.0 | 45428.0 | 45429.3 |
| rs200807 | 6 | 97903674 |  | 45436.0 | 45428.4 | 45428.4 | 45437.9 | 45435.5 | 45430.3 | 45427.8 | 45427.9 | 45430.2 | 45437.3 | 45429.7 | 45429.7 | 45428.4 | 45430.3 | 45427.8 | 45429.7 |
| rs2033529 | 6 | 40348653 |  | 45436.0 | 45428.4 | 45428.4 | 45433.7 | 45437.3 | 45426.2 | 45429.7 | 45429.8 | 45426.1 | 45435.1 | 45427.5 | 45427.5 | 45428.4 | 45426.2 | 45426.1 | 45427.5 |
| rs2228213 | 6 | 12124855 | HIVEP1 | 45436.0 | 45428.4 | 45428.4 | 45437.9 | 45437.6 | 45430.3 | 45430.0 | 45430.0 | 45430.3 | 45439.6 | 45431.9 | 45431.9 | 45428.4 | 45430.3 | 45430.0 | 45431.9 |
| rs3800229 | 6 | 108996963 | FOXO3 | 45436.0 | 45428.4 | 45428.4 | 45432.9 | 45434.1 | 45425.5 | 45426.4 | 45426.6 | 45425.3 | 45431.0 | 45423.5 | 45423.5 | 45428.4 | 45425.5 | 45425.3 | 45423.5 |
| rs539958 | 6 | 160772842 | SLC22A3 | 45436.0 | 45428.4 | 45428.4 | 45438.0 | 45437.4 | 45430.3 | 45429.8 | 45429.8 | 45430.3 | 45439.4 | 45431.7 | 45431.7 | 45428.4 | 45430.3 | 45429.8 | 45431.7 |
| rs6457796 | 6 | 34828553 | UHRF1BP1 | 45436.0 | 45428.4 | 45428.4 | 45434.8 | 45437.7 | 45427.2 | 45430.0 | 45430.1 | 45427.1 | 45436.4 | 45428.8 | 45428.8 | 45428.4 | 45427.2 | 45427.1 | 45428.8 |
| rs6903387 | 6 | 46348834 | RCAN2 | 45436.0 | 45428.4 | 45428.4 | 45437.3 | 45435.1 | 45429.6 | 45427.4 | 45427.3 | 45429.7 | 45436.3 | 45428.6 | 45428.6 | 45428.4 | 45429.6 | 45427.3 | 45428.6 |
| rs9275595 | 6 | 32681355 | XXbac-BPG254F23.7 | 45436.0 | 45428.4 | 45428.4 | 45434.9 | 45437.6 | 45427.3 | 45429.9 | 45430.0 | 45427.3 | 45436.5 | 45428.9 | 45428.9 | 45428.4 | 45427.3 | 45427.3 | 45428.9 |
| rs9364687 | 6 | 163817911 |  | 45436.0 | 45428.4 | 45428.4 | 45437.1 | 45437.0 | 45429.4 | 45429.3 | 45429.3 | 45429.4 | 45438.0 | 45430.3 | 45430.3 | 45428.4 | 45429.4 | 45429.3 | 45430.3 |
| rs9374842 | 6 | 120185665 |  | 45436.0 | 45428.4 | 45428.4 | 45437.0 | 45436.5 | 45429.4 | 45428.8 | 45428.9 | 45429.3 | 45437.5 | 45429.9 | 45429.9 | 45428.4 | 45429.4 | 45428.8 | 45429.9 |
| rs943005 | 6 | 50865820 | RP4-753D5.3 | 45436.0 | 45428.4 | 45428.4 | 45437.0 | 45438.0 | 45429.3 | 45430.3 | 45430.3 | 45429.3 | 45438.9 | 45431.3 | 45431.3 | 45428.4 | 45429.3 | 45429.3 | 45431.3 |
| rs11771526 | 7 | 32342618 | PDE1C | 45436.0 | 45428.4 | 45428.4 | 45438.0 | 45435.6 | 45430.4 | 45428.0 | 45428.0 | 45430.4 | 45437.6 | 45430.0 | 45430.0 | 45428.4 | 45430.4 | 45428.0 | 45430.0 |
| rs10269783 | 7 | 49616203 |  | 45436.0 | 45428.4 | 45428.4 | 45437.6 | 45437.5 | 45430.0 | 45429.8 | 45429.8 | 45429.9 | 45439.0 | 45431.4 | 45431.4 | 45428.4 | 45430.0 | 45429.8 | 45431.4 |
| rs2245368 | 7 | 76608143 | UPK3B | 45436.0 | 45428.4 | 45428.4 | 45421.6 | 45436.8 | 45414.2 | 45429.1 | 45429.3 | 45413.9 | 45422.3 | 45414.9 | 45414.9 | 45428.4 | 45414.2 | 45413.9 | 45414.9 |
| rs9641123 | 7 | 93197732 | CALCR | 45436.0 | 45428.4 | 45428.4 | 45427.1 | 45434.5 | 45419.8 | 45426.9 | 45427.2 | 45419.5 | 45425.6 | 45418.3 | 45418.3 | 45428.4 | 45419.8 | 45419.5 | 45418.3 |
| rs6465468 | 7 | 95169514 | ASB4 | 45436.0 | 45428.4 | 45428.4 | 45437.7 | 45437.0 | 45430.1 | 45429.4 | 45429.4 | 45430.1 | 45438.7 | 45431.1 | 45431.1 | 45428.4 | 45430.1 | 45429.4 | 45431.1 |
| rs10499694 | 7 | 50614173 | DDC | 45436.0 | 45428.4 | 45428.4 | 45437.7 | 45437.3 | 45430.1 | 45429.7 | 45429.7 | 45430.1 | 45439.1 | 45431.4 | 45431.4 | 45428.4 | 45430.1 | 45429.7 | 45431.4 |
| rs1167827 | 7 | 75163169 | HIP1 | 45436.0 | 45428.4 | 45428.4 | 45437.0 | 45430.8 | 45429.5 | 45423.2 | 45423.3 | 45429.4 | 45431.8 | 45424.3 | 45424.3 | 45428.4 | 45429.5 | 45423.2 | 45424.3 |
| rs1593312 | 7 | 131584453 | AC009518.4 | 45436.0 | 45428.4 | 45428.4 | 45433.4 | 45436.1 | 45425.9 | 45428.5 | 45428.6 | 45425.8 | 45433.6 | 45426.1 | 45426.1 | 45428.4 | 45425.9 | 45425.8 | 45426.1 |
| rs1830074 | 7 | 6718674 | RP11-611L7.2 | 45436.0 | 45428.4 | 45428.4 | 45438.0 | 45437.0 | 45430.3 | 45429.4 | 45429.4 | 45430.3 | 45439.0 | 45431.3 | 45431.3 | 45428.4 | 45430.3 | 45429.4 | 45431.3 |
| rs3779273 | 7 | 77828940 | MAGI2 | 45436.0 | 45428.4 | 45428.4 | 45437.7 | 45436.7 | 45430.1 | 45429.0 | 45429.0 | 45430.1 | 45438.4 | 45430.7 | 45430.7 | 45428.4 | 45430.1 | 45429.0 | 45430.7 |
| rs6990042 | 8 | 14173974 | SGCZ | 45436.0 | 45428.4 | 45428.4 | 45435.0 | 45437.8 | 45427.3 | 45430.2 | 45430.1 | 45427.3 | 45436.8 | 45429.1 | 45429.1 | 45428.4 | 45427.3 | 45427.3 | 45429.1 |
| rs11997175 | 8 | 33770070 | RP11-317N12.1 | 45436.0 | 45428.4 | 45428.4 | 45438.0 | 45438.0 | 45430.3 | 45430.3 | 45430.3 | 45430.3 | 45440.0 | 45432.3 | 45432.3 | 45428.4 | 45430.3 | 45430.3 | 45432.3 |
| rs11787111 | 8 | 65192451 | RP11-32K4.1 | 45436.0 | 45428.4 | 45428.4 | 45435.3 | 45436.0 | 45427.7 | 45428.4 | 45428.5 | 45427.6 | 45435.2 | 45427.7 | 45427.7 | 45428.4 | 45427.7 | 45427.6 | 45427.7 |
| rs16907751 | 8 | 81375457 |  | 45436.0 | 45428.4 | 45428.4 | 45435.6 | 45437.9 | 45427.9 | 45430.2 | 45430.2 | 45428.0 | 45437.5 | 45429.8 | 45429.8 | 45428.4 | 45427.9 | 45428.0 | 45429.8 |
| rs12680842 | 8 | 95582606 | RP11-267M23.4 | 45436.0 | 45428.4 | 45428.4 | 45437.9 | 45435.9 | 45430.3 | 45428.2 | 45428.2 | 45430.3 | 45437.8 | 45430.2 | 45430.2 | 45428.4 | 45430.3 | 45428.2 | 45430.2 |
| rs10156366 | 8 | 77246607 |  | 45436.0 | 45428.4 | 45428.4 | 45433.5 | 45437.9 | 45425.9 | 45430.3 | 45430.3 | 45425.9 | 45435.4 | 45427.8 | 45427.8 | 45428.4 | 45425.9 | 45425.9 | 45427.8 |
| rs17149279 | 8 | 9195638 | RP11-115J16.1 | 45436.0 | 45428.4 | 45428.4 | 45436.8 | 45437.9 | 45429.1 | 45430.3 | 45430.3 | 45429.1 | 45438.7 | 45431.0 | 45431.0 | 45428.4 | 45429.1 | 45429.1 | 45431.0 |
| rs2060604 | 8 | 76650334 |  | 45436.0 | 45428.4 | 45428.4 | 45427.1 | 45436.1 | 45419.7 | 45428.4 | 45428.7 | 45419.5 | 45427.2 | 45419.7 | 45419.7 | 45428.4 | 45419.7 | 45419.5 | 45419.7 |
| rs3134353 | 8 | 101947453 | YWHAZ | 45436.0 | 45428.4 | 45428.4 | 45438.0 | 45437.7 | 45430.4 | 45430.0 | 45430.0 | 45430.4 | 45439.7 | 45432.0 | 45432.0 | 45428.4 | 45430.4 | 45430.0 | 45432.0 |
| rs4389974 | 8 | 112381796 |  | 45436.0 | 45428.4 | 45428.4 | 45435.5 | 45430.4 | 45428.1 | 45422.8 | 45423.0 | 45427.9 | 45429.9 | 45422.5 | 45422.5 | 45428.4 | 45428.1 | 45422.8 | 45422.5 |
| rs6985539 | 8 | 62081223 | CLVS1 | 45436.0 | 45428.4 | 45428.4 | 45436.9 | 45431.2 | 45429.4 | 45423.5 | 45423.7 | 45429.2 | 45432.0 | 45424.5 | 45424.5 | 45428.4 | 45429.4 | 45423.5 | 45424.5 |
| rs733594 | 8 | 85077686 | RP11-120I21.3 | 45436.0 | 45428.4 | 45428.4 | 45433.2 | 45436.7 | 45425.4 | 45429.1 | 45428.9 | 45425.6 | 45433.9 | 45426.1 | 45426.1 | 45428.4 | 45425.4 | 45425.6 | 45426.1 |
| rs7844647 | 8 | 34503776 |  | 45436.0 | 45428.4 | 45428.4 | 45437.7 | 45438.0 | 45430.0 | 45430.4 | 45430.4 | 45430.0 | 45439.7 | 45432.0 | 45432.0 | 45428.4 | 45430.0 | 45430.0 | 45432.0 |
| rs10760279 | 9 | 126105291 |  | 45436.0 | 45428.4 | 45428.4 | 45433.5 | 45437.9 | 45425.8 | 45430.3 | 45430.3 | 45425.9 | 45435.4 | 45427.8 | 45427.8 | 45428.4 | 45425.8 | 45425.9 | 45427.8 |
| rs10733682 | 9 | 129460914 | LMX1B | 45436.0 | 45428.4 | 45428.4 | 45430.9 | 45437.8 | 45423.3 | 45430.2 | 45430.2 | 45423.2 | 45432.7 | 45425.1 | 45425.1 | 45428.4 | 45423.3 | 45423.2 | 45425.1 |
| rs2270204 | 9 | 131042734 | SWI5 | 45436.0 | 45428.4 | 45428.4 | 45437.4 | 45437.0 | 45429.7 | 45429.4 | 45429.3 | 45429.8 | 45438.4 | 45430.7 | 45430.7 | 45428.4 | 45429.7 | 45429.3 | 45430.7 |
| rs10975870 | 9 | 6880263 | KDM4C | 45436.0 | 45428.4 | 45428.4 | 45437.9 | 45435.5 | 45430.3 | 45427.9 | 45427.9 | 45430.3 | 45437.4 | 45429.8 | 45429.8 | 45428.4 | 45430.3 | 45427.9 | 45429.8 |
| rs10971721 | 9 | 33827694 | UBE2R2 | 45436.0 | 45428.4 | 45428.4 | 45438.0 | 45434.3 | 45430.3 | 45426.6 | 45426.6 | 45430.4 | 45436.3 | 45428.6 | 45428.6 | 45428.4 | 45430.3 | 45426.6 | 45428.6 |
| rs1928295 | 9 | 120378483 |  | 45436.0 | 45428.4 | 45428.4 | 45431.7 | 45435.3 | 45424.3 | 45427.6 | 45427.8 | 45424.1 | 45431.0 | 45423.6 | 45423.6 | 45428.4 | 45424.3 | 45424.1 | 45423.6 |
| rs2183825 | 9 | 28412375 | LINGO2 | 45436.0 | 45428.4 | 45428.4 | 45435.1 | 45437.7 | 45427.4 | 45430.1 | 45430.0 | 45427.4 | 45436.8 | 45429.1 | 45429.1 | 45428.4 | 45427.4 | 45427.4 | 45429.1 |
| rs4740619 | 9 | 15634326 | CCDC171 | 45436.0 | 45428.4 | 45428.4 | 45437.4 | 45436.9 | 45429.8 | 45429.3 | 45429.3 | 45429.7 | 45438.3 | 45430.7 | 45430.7 | 45428.4 | 45429.8 | 45429.3 | 45430.7 |
| rs6477694 | 9 | 111932342 | FRRS1L | 45436.0 | 45428.4 | 45428.4 | 45433.7 | 45438.0 | 45426.1 | 45430.4 | 45430.4 | 45426.1 | 45435.7 | 45428.1 | 45428.1 | 45428.4 | 45426.1 | 45426.1 | 45428.1 |
| rs17094222 | 10 | 102395440 |  | 45436.0 | 45428.4 | 45428.4 | 45430.6 | 45436.0 | 45422.8 | 45428.4 | 45428.2 | 45423.0 | 45430.6 | 45422.8 | 45422.8 | 45428.4 | 45422.8 | 45423.0 | 45422.8 |
| rs718948 | 10 | 126739338 | CTBP2 | 45436.0 | 45428.4 | 45428.4 | 45433.7 | 45436.0 | 45425.9 | 45428.4 | 45428.2 | 45426.0 | 45433.7 | 45425.9 | 45425.9 | 45428.4 | 45425.9 | 45426.0 | 45425.9 |
| rs11191343 | 10 | 104345225 | SUFU | 45436.0 | 45428.4 | 45428.4 | 45437.1 | 45436.6 | 45429.5 | 45428.9 | 45429.0 | 45429.4 | 45437.7 | 45430.1 | 45430.1 | 45428.4 | 45429.5 | 45428.9 | 45430.1 |
| rs12220375 | 10 | 104901491 | NT5C2 | 45436.0 | 45428.4 | 45428.4 | 45432.8 | 45437.1 | 45425.2 | 45429.5 | 45429.6 | 45425.1 | 45433.8 | 45426.3 | 45426.3 | 45428.4 | 45425.2 | 45425.1 | 45426.3 |
| rs751008 | 10 | 129142417 | DOCK1 | 45436.0 | 45428.4 | 45428.4 | 45437.9 | 45436.2 | 45430.3 | 45428.5 | 45428.6 | 45430.3 | 45438.1 | 45430.5 | 45430.5 | 45428.4 | 45430.3 | 45428.5 | 45430.5 |
| rs7899106 | 10 | 87410904 | GRID1 | 45436.0 | 45428.4 | 45428.4 | 45435.7 | 45435.0 | 45427.9 | 45427.3 | 45427.2 | 45428.1 | 45434.7 | 45426.9 | 45426.9 | 45428.4 | 45427.9 | 45427.2 | 45426.9 |
| rs7903146 | 10 | 114758349 | TCF7L2 | 45436.0 | 45428.4 | 45428.4 | 45429.5 | 45438.0 | 45421.9 | 45430.4 | 45430.4 | 45421.9 | 45431.5 | 45423.9 | 45423.9 | 45428.4 | 45421.9 | 45421.9 | 45423.9 |
| rs1557765 | 11 | 17403639 | NCR3LG1 | 45436.0 | 45428.4 | 45428.4 | 45437.6 | 45438.0 | 45429.9 | 45430.4 | 45430.4 | 45429.9 | 45439.6 | 45431.9 | 45431.9 | 45428.4 | 45429.9 | 45429.9 | 45431.9 |
| rs10742752 | 11 | 45438374 | RP11-430H10.4 | 45436.0 | 45428.4 | 45428.4 | 45431.9 | 45438.0 | 45424.2 | 45430.3 | 45430.3 | 45424.2 | 45433.9 | 45426.2 | 45426.2 | 45428.4 | 45424.2 | 45424.2 | 45426.2 |
| rs10540 | 11 | 494662 | RNH1 | 45436.0 | 45428.4 | 45428.4 | 45437.6 | 45434.7 | 45430.0 | 45427.1 | 45427.1 | 45429.9 | 45436.3 | 45428.7 | 45428.7 | 45428.4 | 45430.0 | 45427.1 | 45428.7 |
| rs10840100 | 11 | 8669437 | TRIM66 | 45436.0 | 45428.4 | 45428.4 | 45434.4 | 45435.3 | 45426.6 | 45427.6 | 45427.4 | 45426.7 | 45433.6 | 45425.8 | 45425.8 | 45428.4 | 45426.6 | 45426.7 | 45425.8 |
| rs11030104 | 11 | 27684517 | BDNF | 45436.0 | 45428.4 | 45428.4 | 45433.6 | 45434.8 | 45426.2 | 45427.2 | 45427.3 | 45426.0 | 45432.4 | 45424.9 | 45424.9 | 45428.4 | 45426.2 | 45426.0 | 45424.9 |
| rs11607976 | 11 | 69279111 |  | 45436.0 | 45428.4 | 45428.4 | 45435.7 | 45438.0 | 45428.1 | 45430.4 | 45430.4 | 45428.1 | 45437.7 | 45430.1 | 45430.1 | 45428.4 | 45428.1 | 45428.1 | 45430.1 |
| rs12286929 | 11 | 115022404 |  | 45436.0 | 45428.4 | 45428.4 | 45429.9 | 45437.2 | 45422.2 | 45429.5 | 45429.4 | 45422.3 | 45431.1 | 45423.3 | 45423.3 | 45428.4 | 45422.2 | 45422.3 | 45423.3 |
| rs1552224 | 11 | 72433098 | ARAP1 | 45436.0 | 45428.4 | 45428.4 | 45433.4 | 45436.9 | 45425.9 | 45429.2 | 45429.3 | 45425.8 | 45434.3 | 45426.7 | 45426.7 | 45428.4 | 45425.9 | 45425.8 | 45426.7 |
| rs1816537 | 11 | 112968651 | NCAM1 | 45436.0 | 45428.4 | 45428.4 | 45437.8 | 45435.0 | 45430.1 | 45427.3 | 45427.3 | 45430.1 | 45436.7 | 45429.0 | 45429.0 | 45428.4 | 45430.1 | 45427.3 | 45429.0 |
| rs2176598 | 11 | 43864278 | HSD17B12 | 45436.0 | 45428.4 | 45428.4 | 45437.2 | 45436.5 | 45429.6 | 45428.9 | 45428.9 | 45429.6 | 45437.7 | 45430.1 | 45430.1 | 45428.4 | 45429.6 | 45428.9 | 45430.1 |
| rs2845885 | 11 | 63869062 | MACROD1 | 45436.0 | 45428.4 | 45428.4 | 45438.0 | 45437.3 | 45430.3 | 45429.6 | 45429.6 | 45430.3 | 45439.2 | 45431.5 | 45431.5 | 45428.4 | 45430.3 | 45429.6 | 45431.5 |
| rs3817334 | 11 | 47650993 | MTCH2 | 45436.0 | 45428.4 | 45428.4 | 45425.5 | 45437.5 | 45417.8 | 45429.8 | 45429.7 | 45417.9 | 45427.0 | 45419.2 | 45419.2 | 45428.4 | 45417.8 | 45417.9 | 45419.2 |
| rs4757142 | 11 | 13325695 | ARNTL | 45436.0 | 45428.4 | 45428.4 | 45432.6 | 45438.0 | 45424.9 | 45430.4 | 45430.4 | 45424.9 | 45434.6 | 45426.9 | 45426.9 | 45428.4 | 45424.9 | 45424.9 | 45426.9 |
| rs17033633 | 12 | 103656343 | C12orf42 | 45436.0 | 45428.4 | 45428.4 | 45437.9 | 45437.8 | 45430.3 | 45430.1 | 45430.1 | 45430.3 | 45439.7 | 45432.0 | 45432.0 | 45428.4 | 45430.3 | 45430.1 | 45432.0 |
| rs1502337 | 12 | 111062852 | TCTN1 | 45436.0 | 45428.4 | 45428.4 | 45433.6 | 45437.4 | 45426.1 | 45429.7 | 45429.8 | 45426.0 | 45435.0 | 45427.4 | 45427.4 | 45428.4 | 45426.1 | 45426.0 | 45427.4 |
| rs11057405 | 12 | 122781897 | CLIP1 | 45436.0 | 45428.4 | 45428.4 | 45437.6 | 45436.8 | 45429.9 | 45429.1 | 45429.1 | 45430.0 | 45438.4 | 45430.7 | 45430.7 | 45428.4 | 45429.9 | 45429.1 | 45430.7 |
| rs11170468 | 12 | 39430048 |  | 45436.0 | 45428.4 | 45428.4 | 45437.3 | 45436.8 | 45429.7 | 45429.2 | 45429.2 | 45429.7 | 45438.1 | 45430.5 | 45430.5 | 45428.4 | 45429.7 | 45429.2 | 45430.5 |
| rs285575 | 12 | 41921665 | PDZRN4 | 45436.0 | 45428.4 | 45428.4 | 45424.9 | 45436.7 | 45417.4 | 45429.0 | 45429.2 | 45417.2 | 45425.5 | 45418.1 | 45418.1 | 45428.4 | 45417.4 | 45417.2 | 45418.1 |
| rs11611246 | 12 | 939480 | WNK1 | 45436.0 | 45428.4 | 45428.4 | 45438.0 | 45435.5 | 45430.3 | 45427.9 | 45427.9 | 45430.3 | 45437.5 | 45429.8 | 45429.8 | 45428.4 | 45430.3 | 45427.9 | 45429.8 |
| rs10773049 | 12 | 124506631 | FAM101A | 45436.0 | 45428.4 | 45428.4 | 45436.5 | 45438.0 | 45428.9 | 45430.3 | 45430.3 | 45428.9 | 45438.5 | 45430.8 | 45430.8 | 45428.4 | 45428.9 | 45428.9 | 45430.8 |
| rs11065987 | 12 | 112072424 |  | 45436.0 | 45428.4 | 45428.4 | 45426.4 | 45437.9 | 45418.7 | 45430.3 | 45430.2 | 45418.7 | 45428.3 | 45420.6 | 45420.6 | 45428.4 | 45418.7 | 45418.7 | 45420.6 |
| rs11168854 | 12 | 49485373 | DHH | 45436.0 | 45428.4 | 45428.4 | 45434.3 | 45437.3 | 45426.8 | 45429.6 | 45429.7 | 45426.7 | 45435.6 | 45428.0 | 45428.0 | 45428.4 | 45426.8 | 45426.7 | 45428.0 |
| rs11247009 | 12 | 132701184 | GALNT9 | 45436.0 | 45428.4 | 45428.4 | 45438.0 | 45436.4 | 45430.3 | 45428.7 | 45428.7 | 45430.3 | 45438.3 | 45430.7 | 45430.7 | 45428.4 | 45430.3 | 45428.7 | 45430.7 |
| rs17630235 | 12 | 112591686 | TRAFD1 | 45436.0 | 45428.4 | 45428.4 | 45427.4 | 45438.0 | 45419.8 | 45430.4 | 45430.4 | 45419.8 | 45429.4 | 45421.8 | 45421.8 | 45428.4 | 45419.8 | 45419.8 | 45421.8 |
| rs2579106 | 12 | 90628230 |  | 45436.0 | 45428.4 | 45428.4 | 45434.3 | 45434.2 | 45426.5 | 45426.6 | 45426.4 | 45426.7 | 45432.5 | 45424.7 | 45424.7 | 45428.4 | 45426.5 | 45426.4 | 45424.7 |
| rs2720298 | 12 | 50189807 |  | 45436.0 | 45428.4 | 45428.4 | 45431.4 | 45438.0 | 45423.8 | 45430.4 | 45430.3 | 45423.8 | 45433.4 | 45425.8 | 45425.8 | 45428.4 | 45423.8 | 45423.8 | 45425.8 |
| rs7138803 | 12 | 50247468 |  | 45436.0 | 45428.4 | 45428.4 | 45431.0 | 45437.9 | 45423.4 | 45430.3 | 45430.3 | 45423.4 | 45432.9 | 45425.3 | 45425.3 | 45428.4 | 45423.4 | 45423.4 | 45425.3 |
| rs9651934 | 12 | 89355709 |  | 45436.0 | 45428.4 | 45428.4 | 45436.8 | 45437.1 | 45429.2 | 45429.4 | 45429.5 | 45429.2 | 45437.9 | 45430.3 | 45430.3 | 45428.4 | 45429.2 | 45429.2 | 45430.3 |
| rs9540493 | 13 | 66205704 |  | 45436.0 | 45428.4 | 45428.4 | 45434.5 | 45437.4 | 45426.9 | 45429.8 | 45429.8 | 45426.9 | 45435.9 | 45428.3 | 45428.3 | 45428.4 | 45426.9 | 45426.9 | 45428.3 |
| rs1441264 | 13 | 79580919 |  | 45436.0 | 45428.4 | 45428.4 | 45437.9 | 45437.4 | 45430.2 | 45429.8 | 45429.8 | 45430.2 | 45439.3 | 45431.6 | 45431.6 | 45428.4 | 45430.2 | 45429.8 | 45431.6 |
| rs1164586 | 13 | 97049141 | HS6ST3 | 45436.0 | 45428.4 | 45428.4 | 45435.0 | 45438.0 | 45427.3 | 45430.4 | 45430.4 | 45427.3 | 45436.9 | 45429.3 | 45429.3 | 45428.4 | 45427.3 | 45427.3 | 45429.3 |
| rs12429545 | 13 | 54102206 |  | 45436.0 | 45428.4 | 45428.4 | 45431.1 | 45438.0 | 45423.4 | 45430.3 | 45430.4 | 45423.4 | 45433.0 | 45425.4 | 45425.4 | 45428.4 | 45423.4 | 45423.4 | 45425.4 |
| rs7332115 | 13 | 33147548 |  | 45436.0 | 45428.4 | 45428.4 | 45438.0 | 45437.8 | 45430.3 | 45430.2 | 45430.2 | 45430.3 | 45439.8 | 45432.1 | 45432.1 | 45428.4 | 45430.3 | 45430.2 | 45432.1 |
| rs9507983 | 13 | 28620036 | FLT3 | 45436.0 | 45428.4 | 45428.4 | 45437.8 | 45437.7 | 45430.1 | 45430.1 | 45430.1 | 45430.1 | 45439.5 | 45431.8 | 45431.8 | 45428.4 | 45430.1 | 45430.1 | 45431.8 |
| rs9563576 | 13 | 58670147 |  | 45436.0 | 45428.4 | 45428.4 | 45434.0 | 45434.9 | 45426.2 | 45427.3 | 45427.1 | 45426.3 | 45432.9 | 45425.1 | 45425.1 | 45428.4 | 45426.2 | 45426.3 | 45425.1 |
| rs12016871 | 13 | 28017782 | MTIF3 | 45436.0 | 45428.4 | 45428.4 | 45433.7 | 45435.2 | 45425.9 | 45427.5 | 45427.3 | 45426.1 | 45432.9 | 45425.0 | 45425.0 | 45428.4 | 45425.9 | 45426.1 | 45425.0 |
| rs17522122 | 14 | 33302882 | AKAP6 | 45436.0 | 45428.4 | 45428.4 | 45430.5 | 45434.9 | 45423.1 | 45427.3 | 45427.5 | 45422.9 | 45429.4 | 45422.0 | 45422.0 | 45428.4 | 45423.1 | 45422.9 | 45422.0 |
| rs12894211 | 14 | 69794551 | GALNT16 | 45436.0 | 45428.4 | 45428.4 | 45438.0 | 45427.3 | 45430.4 | 45419.6 | 45419.6 | 45430.4 | 45429.3 | 45421.6 | 45421.6 | 45428.4 | 45427.3 | 45419.6 | 45421.6 |
| rs3783890 | 14 | 93790276 | BTBD7 | 45436.0 | 45428.4 | 45428.4 | 45438.0 | 45437.9 | 45430.4 | 45430.3 | 45430.3 | 45430.4 | 45439.9 | 45432.3 | 45432.3 | 45428.4 | 45430.4 | 45430.3 | 45432.3 |
| rs10132280 | 14 | 25928179 |  | 45436.0 | 45428.4 | 45428.4 | 45433.5 | 45437.9 | 45425.9 | 45430.3 | 45430.3 | 45425.9 | 45435.5 | 45427.9 | 45427.9 | 45428.4 | 45425.9 | 45425.9 | 45427.9 |
| rs12885454 | 14 | 29736838 | RP11-562L8.1 | 45436.0 | 45428.4 | 45428.4 | 45434.6 | 45437.6 | 45427.0 | 45430.0 | 45430.0 | 45427.0 | 45436.3 | 45428.7 | 45428.7 | 45428.4 | 45427.0 | 45427.0 | 45428.7 |
| rs709400 | 14 | 104149475 | RP11-73M18.2 | 45436.0 | 45428.4 | 45428.4 | 45437.9 | 45426.8 | 45430.2 | 45419.2 | 45419.1 | 45430.2 | 45428.7 | 45421.0 | 45421.0 | 45428.4 | 45426.8 | 45419.1 | 45421.0 |
| rs7143963 | 14 | 103304425 | TRAF3 | 45436.0 | 45428.4 | 45428.4 | 45437.8 | 45438.0 | 45430.2 | 45430.4 | 45430.4 | 45430.2 | 45439.8 | 45432.2 | 45432.2 | 45428.4 | 45430.2 | 45430.2 | 45432.2 |
| rs7144011 | 14 | 79940383 | NRXN3 | 45436.0 | 45428.4 | 45428.4 | 45437.0 | 45436.3 | 45429.2 | 45428.6 | 45428.5 | 45429.3 | 45437.2 | 45429.5 | 45429.5 | 45428.4 | 45429.2 | 45428.5 | 45429.5 |
| rs8016859 | 14 | 30484722 | PRKD1 | 45436.0 | 45428.4 | 45428.4 | 45425.5 | 45437.9 | 45417.8 | 45430.3 | 45430.2 | 45417.9 | 45427.4 | 45419.7 | 45419.7 | 45428.4 | 45417.8 | 45417.9 | 45419.7 |
| rs7164727 | 15 | 73093991 | ADPGK-AS1 | 45436.0 | 45428.4 | 45428.4 | 45434.9 | 45437.5 | 45427.3 | 45429.8 | 45429.9 | 45427.2 | 45436.3 | 45428.7 | 45428.7 | 45428.4 | 45427.3 | 45427.2 | 45428.7 |
| rs12593036 | 15 | 81058652 |  | 45436.0 | 45428.4 | 45428.4 | 45438.0 | 45435.5 | 45430.3 | 45427.8 | 45427.8 | 45430.3 | 45437.4 | 45429.8 | 45429.8 | 45428.4 | 45430.3 | 45427.8 | 45429.8 |
| rs13329567 | 15 | 68104367 | MAP2K5 | 45436.0 | 45428.4 | 45428.4 | 45435.2 | 45436.6 | 45427.7 | 45429.0 | 45429.1 | 45427.6 | 45435.8 | 45428.3 | 45428.3 | 45428.4 | 45427.7 | 45427.6 | 45428.3 |
| rs1439620 | 15 | 93429646 | LINC01578 | 45436.0 | 45428.4 | 45428.4 | 45437.6 | 45437.8 | 45430.0 | 45430.2 | 45430.2 | 45430.0 | 45439.4 | 45431.8 | 45431.8 | 45428.4 | 45430.0 | 45430.0 | 45431.8 |
| rs3736485 | 15 | 51748610 | DMXL2 | 45436.0 | 45428.4 | 45428.4 | 45437.5 | 45436.6 | 45429.8 | 45429.0 | 45428.9 | 45429.8 | 45438.1 | 45430.4 | 45430.4 | 45428.4 | 45429.8 | 45428.9 | 45430.4 |
| rs4984406 | 15 | 95268494 |  | 45436.0 | 45428.4 | 45428.4 | 45433.8 | 45437.5 | 45426.2 | 45429.8 | 45429.9 | 45426.1 | 45435.2 | 45427.7 | 45427.7 | 45428.4 | 45426.2 | 45426.1 | 45427.7 |
| rs12446632 | 16 | 19935389 |  | 45436.0 | 45428.4 | 45428.4 | 45436.2 | 45437.4 | 45428.5 | 45429.8 | 45429.7 | 45428.5 | 45437.6 | 45429.9 | 45429.9 | 45428.4 | 45428.5 | 45428.5 | 45429.9 |
| rs1421085 | 16 | 53800954 | FTO | 45436.0 | 45428.4 | 45428.4 | 45406.6 | 45438.0 | 45398.9 | 45430.4 | 45430.3 | 45398.9 | 45408.6 | 45400.9 | 45400.9 | 45428.4 | 45398.9 | 45398.9 | 45400.9 |
| rs2307022 | 16 | 68381978 | PRMT7 | 45436.0 | 45428.4 | 45428.4 | 45438.0 | 45436.7 | 45430.3 | 45429.0 | 45429.0 | 45430.3 | 45438.6 | 45430.9 | 45430.9 | 45428.4 | 45430.3 | 45429.0 | 45430.9 |
| rs889398 | 16 | 69556715 |  | 45436.0 | 45428.4 | 45428.4 | 45433.2 | 45436.5 | 45425.7 | 45428.8 | 45429.0 | 45425.5 | 45433.6 | 45426.1 | 45426.1 | 45428.4 | 45425.7 | 45425.5 | 45426.1 |
| rs2650492 | 16 | 28333411 | SBK1 | 45436.0 | 45428.4 | 45428.4 | 45438.0 | 45435.7 | 45430.3 | 45428.0 | 45428.0 | 45430.3 | 45437.6 | 45430.0 | 45430.0 | 45428.4 | 45430.3 | 45428.0 | 45430.0 |
| rs12448257 | 16 | 3599655 | NLRC3 | 45436.0 | 45428.4 | 45428.4 | 45436.8 | 45437.3 | 45429.2 | 45429.6 | 45429.7 | 45429.2 | 45438.1 | 45430.5 | 45430.5 | 45428.4 | 45429.2 | 45429.2 | 45430.5 |
| rs2080454 | 16 | 49062590 |  | 45436.0 | 45428.4 | 45428.4 | 45436.3 | 45436.3 | 45428.6 | 45428.7 | 45428.6 | 45428.7 | 45436.6 | 45428.9 | 45428.9 | 45428.4 | 45428.6 | 45428.6 | 45428.9 |
| rs2885415 | 16 | 395396 | AXIN1 | 45436.0 | 45428.4 | 45428.4 | 45437.0 | 45437.0 | 45429.3 | 45429.4 | 45429.3 | 45429.4 | 45438.0 | 45430.3 | 45430.3 | 45428.4 | 45429.3 | 45429.3 | 45430.3 |
| rs3888190 | 16 | 28889486 | ATP2A1 | 45436.0 | 45428.4 | 45428.4 | 45433.0 | 45433.0 | 45425.6 | 45425.4 | 45425.6 | 45425.3 | 45430.0 | 45422.6 | 45422.6 | 45428.4 | 45425.6 | 45425.3 | 45422.6 |
| rs4787491 | 16 | 30015337 | INO80E | 45436.0 | 45428.4 | 45428.4 | 45437.5 | 45438.0 | 45429.9 | 45430.3 | 45430.3 | 45429.9 | 45439.5 | 45431.8 | 45431.8 | 45428.4 | 45429.9 | 45429.9 | 45431.8 |
| rs4985155 | 16 | 15129459 | PDXDC1 | 45436.0 | 45428.4 | 45428.4 | 45438.0 | 45434.2 | 45430.4 | 45426.6 | 45426.5 | 45430.4 | 45436.2 | 45428.5 | 45428.5 | 45428.4 | 45430.4 | 45426.5 | 45428.5 |
| rs7189501 | 16 | 6175429 | RBFOX1 | 45436.0 | 45428.4 | 45428.4 | 45436.3 | 45434.8 | 45428.5 | 45427.1 | 45427.0 | 45428.6 | 45435.0 | 45427.2 | 45427.2 | 45428.4 | 45428.5 | 45427.0 | 45427.2 |
| rs749767 | 16 | 31124407 | KAT8 | 45436.0 | 45428.4 | 45428.4 | 45436.8 | 45437.0 | 45429.2 | 45429.3 | 45429.4 | 45429.2 | 45437.8 | 45430.2 | 45430.2 | 45428.4 | 45429.2 | 45429.2 | 45430.2 |
| rs12150665 | 17 | 34914787 |  | 45436.0 | 45428.4 | 45428.4 | 45436.5 | 45437.5 | 45428.9 | 45429.8 | 45429.9 | 45428.9 | 45438.0 | 45430.4 | 45430.4 | 45428.4 | 45428.9 | 45428.9 | 45430.4 |
| rs7223966 | 17 | 61893398 | DDX42 | 45436.0 | 45428.4 | 45428.4 | 45437.9 | 45437.8 | 45430.3 | 45430.2 | 45430.2 | 45430.3 | 45439.7 | 45432.1 | 45432.1 | 45428.4 | 45430.3 | 45430.2 | 45432.1 |
| rs1000940 | 17 | 5283252 | RABEP1 | 45436.0 | 45428.4 | 45428.4 | 45435.9 | 45436.5 | 45428.2 | 45428.9 | 45428.8 | 45428.3 | 45436.4 | 45428.6 | 45428.6 | 45428.4 | 45428.2 | 45428.3 | 45428.6 |
| rs12940622 | 17 | 78615571 | RPTOR | 45436.0 | 45428.4 | 45428.4 | 45420.5 | 45438.0 | 45412.9 | 45430.3 | 45430.3 | 45412.9 | 45422.4 | 45414.8 | 45414.8 | 45428.4 | 45412.9 | 45412.9 | 45414.8 |
| rs16941731 | 17 | 45317022 |  | 45436.0 | 45428.4 | 45428.4 | 45438.0 | 45437.3 | 45430.3 | 45429.7 | 45429.7 | 45430.4 | 45439.3 | 45431.7 | 45431.7 | 45428.4 | 45430.3 | 45429.7 | 45431.7 |
| rs4986044 | 17 | 21261560 |  | 45436.0 | 45428.4 | 45428.4 | 45437.8 | 45436.9 | 45430.2 | 45429.3 | 45429.3 | 45430.2 | 45438.7 | 45431.1 | 45431.1 | 45428.4 | 45430.2 | 45429.3 | 45431.1 |
| rs6504108 | 17 | 46292923 | SKAP1 | 45436.0 | 45428.4 | 45428.4 | 45438.0 | 45437.0 | 45430.4 | 45429.3 | 45429.3 | 45430.4 | 45439.0 | 45431.3 | 45431.3 | 45428.4 | 45430.4 | 45429.3 | 45431.3 |
| rs9914578 | 17 | 2005136 | SMG6 | 45436.0 | 45428.4 | 45428.4 | 45436.5 | 45437.9 | 45428.9 | 45430.2 | 45430.3 | 45428.9 | 45438.4 | 45430.8 | 45430.8 | 45428.4 | 45428.9 | 45428.9 | 45430.8 |
| rs7226371 | 18 | 1850771 |  | 45436.0 | 45428.4 | 45428.4 | 45434.8 | 45437.0 | 45427.2 | 45429.3 | 45429.4 | 45427.2 | 45435.8 | 45428.2 | 45428.2 | 45428.4 | 45427.2 | 45427.2 | 45428.2 |
| rs7243357 | 18 | 56883319 | GRP | 45436.0 | 45428.4 | 45428.4 | 45435.5 | 45436.4 | 45427.8 | 45428.7 | 45428.6 | 45427.9 | 45435.9 | 45428.1 | 45428.1 | 45428.4 | 45427.8 | 45427.9 | 45428.1 |
| rs12454712 | 18 | 60845884 | BCL2 | 45436.0 | 45428.4 | 45428.4 | 45435.2 | 45437.8 | 45427.6 | 45430.2 | 45430.2 | 45427.5 | 45437.0 | 45429.4 | 45429.4 | 45428.4 | 45427.6 | 45427.5 | 45429.4 |
| rs12961799 | 18 | 12904399 | PTPN2 | 45436.0 | 45428.4 | 45428.4 | 45437.5 | 45435.6 | 45429.8 | 45427.9 | 45427.9 | 45429.8 | 45437.0 | 45429.3 | 45429.3 | 45428.4 | 45429.8 | 45427.9 | 45429.3 |
| rs1788820 | 18 | 21101944 | C18orf8 | 45436.0 | 45428.4 | 45428.4 | 45432.2 | 45436.9 | 45424.4 | 45429.2 | 45429.1 | 45424.5 | 45433.0 | 45425.2 | 45425.2 | 45428.4 | 45424.4 | 45424.5 | 45425.2 |
| rs555267 | 18 | 40992698 |  | 45436.0 | 45428.4 | 45428.4 | 45437.7 | 45437.0 | 45430.0 | 45429.3 | 45429.3 | 45430.1 | 45438.7 | 45431.0 | 45431.0 | 45428.4 | 45430.0 | 45429.3 | 45431.0 |
| rs6567160 | 18 | 57829135 | RNU4-17P | 45436.0 | 45428.4 | 45428.4 | 45425.8 | 45433.8 | 45418.4 | 45426.1 | 45426.5 | 45418.1 | 45423.5 | 45416.2 | 45416.2 | 45428.4 | 45418.4 | 45418.1 | 45416.2 |
| rs7239883 | 18 | 40147671 | LINC00907 | 45436.0 | 45428.4 | 45428.4 | 45436.0 | 45437.7 | 45428.4 | 45430.0 | 45430.0 | 45428.4 | 45437.7 | 45430.0 | 45430.0 | 45428.4 | 45428.4 | 45428.4 | 45430.0 |
| rs11672550 | 19 | 1937193 | CSNK1G2 | 45436.0 | 45428.4 | 45428.4 | 45437.8 | 45437.2 | 45430.2 | 45429.6 | 45429.5 | 45430.2 | 45439.0 | 45431.4 | 45431.4 | 45428.4 | 45430.2 | 45429.5 | 45431.4 |
| rs2304130 | 19 | 19789528 | ZNF101 | 45436.0 | 45428.4 | 45428.4 | 45437.1 | 45438.0 | 45429.5 | 45430.4 | 45430.4 | 45429.5 | 45439.1 | 45431.5 | 45431.5 | 45428.4 | 45429.5 | 45429.5 | 45431.5 |
| rs17513613 | 19 | 30286822 |  | 45436.0 | 45428.4 | 45428.4 | 45435.8 | 45437.9 | 45428.2 | 45430.2 | 45430.2 | 45428.2 | 45437.7 | 45430.1 | 45430.1 | 45428.4 | 45428.2 | 45428.2 | 45430.1 |
| rs33439 | 19 | 30945171 | ZNF536 | 45436.0 | 45428.4 | 45428.4 | 45437.7 | 45437.5 | 45430.1 | 45429.8 | 45429.8 | 45430.1 | 45439.2 | 45431.5 | 45431.5 | 45428.4 | 45430.1 | 45429.8 | 45431.5 |
| rs2075650 | 19 | 45395619 | TOMM40 | 45436.0 | 45428.4 | 45428.4 | 45438.0 | 45433.3 | 45430.3 | 45425.7 | 45425.7 | 45430.3 | 45435.3 | 45427.7 | 45427.7 | 45428.4 | 45430.3 | 45425.7 | 45427.7 |
| rs3810291 | 19 | 47569003 | ZC3H4 | 45436.0 | 45428.4 | 45428.4 | 45437.0 | 45437.8 | 45429.3 | 45430.2 | 45430.2 | 45429.3 | 45438.7 | 45431.1 | 45431.1 | 45428.4 | 45429.3 | 45429.3 | 45431.1 |
| rs11672660 | 19 | 46180184 | GIPR | 45436.0 | 45428.4 | 45428.4 | 45421.7 | 45436.6 | 45414.3 | 45429.0 | 45429.2 | 45414.1 | 45422.3 | 45414.9 | 45414.9 | 45428.4 | 45414.3 | 45414.1 | 45414.9 |
| rs14810 | 19 | 34304903 | KCTD15 | 45436.0 | 45428.4 | 45428.4 | 45436.9 | 45435.8 | 45429.3 | 45428.2 | 45428.2 | 45429.3 | 45436.7 | 45429.1 | 45429.1 | 45428.4 | 45429.3 | 45428.2 | 45429.1 |
| rs17724992 | 19 | 18454825 | PGPEP1 | 45436.0 | 45428.4 | 45428.4 | 45434.8 | 45437.8 | 45427.2 | 45430.2 | 45430.2 | 45427.2 | 45436.7 | 45429.0 | 45429.0 | 45428.4 | 45427.2 | 45427.2 | 45429.0 |
| rs8123881 | 20 | 15819495 | MACROD2 | 45436.0 | 45428.4 | 45428.4 | 45437.7 | 45436.3 | 45430.1 | 45428.7 | 45428.7 | 45430.1 | 45438.1 | 45430.4 | 45430.4 | 45428.4 | 45430.1 | 45428.7 | 45430.4 |
| rs1884897 | 20 | 6612832 |  | 45436.0 | 45428.4 | 45428.4 | 45437.9 | 45437.3 | 45430.2 | 45429.6 | 45429.6 | 45430.2 | 45439.1 | 45431.5 | 45431.5 | 45428.4 | 45430.2 | 45429.6 | 45431.5 |
| rs2236176 | 20 | 21081488 | LINC00237 | 45436.0 | 45428.4 | 45428.4 | 45434.2 | 45437.6 | 45426.5 | 45430.0 | 45429.9 | 45426.6 | 45435.8 | 45428.1 | 45428.1 | 45428.4 | 45426.5 | 45426.6 | 45428.1 |
| rs4809401 | 20 | 62737568 | NPBWR2 | 45436.0 | 45428.4 | 45428.4 | 45432.8 | 45438.0 | 45425.2 | 45430.3 | 45430.4 | 45425.1 | 45434.8 | 45427.1 | 45427.1 | 45428.4 | 45425.2 | 45425.1 | 45427.1 |
| rs6010784 | 20 | 61540319 | DIDO1 | 45436.0 | 45428.4 | 45428.4 | 45437.9 | 45438.0 | 45430.3 | 45430.4 | 45430.4 | 45430.3 | 45439.9 | 45432.2 | 45432.2 | 45428.4 | 45430.3 | 45430.3 | 45432.2 |
| rs6091540 | 20 | 51087862 | LINC01524 | 45436.0 | 45428.4 | 45428.4 | 45429.1 | 45437.1 | 45421.6 | 45429.5 | 45429.6 | 45421.5 | 45430.2 | 45422.7 | 45422.7 | 45428.4 | 45421.6 | 45421.5 | 45422.7 |
| rs2836754 | 21 | 40291740 | AF064858.6 | 45436.0 | 45428.4 | 45428.4 | 45437.3 | 45434.7 | 45429.5 | 45427.0 | 45426.9 | 45429.6 | 45435.9 | 45428.2 | 45428.2 | 45428.4 | 45429.5 | 45426.9 | 45428.2 |
| rs427943 | 21 | 46570896 | ADARB1 | 45436.0 | 45428.4 | 45428.4 | 45436.3 | 45435.4 | 45428.6 | 45427.7 | 45427.6 | 45428.7 | 45435.7 | 45427.9 | 45427.9 | 45428.4 | 45428.6 | 45427.6 | 45427.9 |
| rs914187 | 21 | 42619749 | BACE2 | 45436.0 | 45428.4 | 45428.4 | 45437.5 | 45432.3 | 45429.7 | 45424.6 | 45424.6 | 45429.8 | 45433.7 | 45426.0 | 45426.0 | 45428.4 | 45429.7 | 45424.6 | 45426.0 |
| rs134871 | 22 | 42652716 | TCF20 | 45436.0 | 45428.4 | 45428.4 | 45436.2 | 45437.7 | 45428.5 | 45430.0 | 45430.0 | 45428.5 | 45437.8 | 45430.2 | 45430.2 | 45428.4 | 45428.5 | 45428.5 | 45430.2 |
| rs4820408 | 22 | 40604945 | TNRC6B | 45436.0 | 45428.4 | 45428.4 | 45432.0 | 45437.3 | 45424.4 | 45429.7 | 45429.8 | 45424.3 | 45433.3 | 45425.7 | 45425.7 | 45428.4 | 45424.4 | 45424.3 | 45425.7 |
