# Supplementary material for: Pleiotropy of genetic variants on obesity and smoking phenotypes: Results from the Oncoarray Project of The International Lung Cancer Consortium
Source: PLoS One. 2017 Sep 28;12(9):e0185660. doi: 10.1371/journal.pone.0185660 (PMC5619832; doi:10.1371/journal.pone.0185660)
Supplement: S2 Table — (DOCX) [file pone.0185660.s002.docx]

S2 Table:

The comparison of associations between different BMI-GRSs and smoking categories (n=17,037)

| **Category** | **Before adjustment for BMI** | | | **After adjustment for BMI** | | |
| --- | --- | --- | --- | --- | --- | --- |
|  | **Coef** | **se** | **p-value** | **Coef** | **se** | **p-value** |
| Unweighted GRS based on 241 SNPs |  |  |  |  |  |  |
| Current-Smokers | 0.549 | 0.236 | 0.021 | 0.838 | 0.236 | <0.001 |
| Ex-smokers | 0.490 | 0.229 | 0.032 | 0.321 | 0.227 | 0.157 |
| Smokers | 0.516 | 0.21 | 0.016 | 0.545 | 0.212 | 0.010 |
| Unweighted GRS based on 97 SNPs |  |  |  |  |  |  |
| Current-Smokers | 0.382 | 0.147 | 0.009 | 0.538 | 0.146 | <0.001 |
| Ex-smokers | 0.284 | 0.141 | 0.044 | 0.193 | 0.140 | 0.169 |
| Smokers | 0.327 | 0.132 | 0.013 | 0.343 | 0.131 | 0.009 |
| Weighted GRS based on 241 SNPs |  |  |  |  |  |  |
| Current-Smokers | 0.015 | 0.005 | 0.0029 | 0.022 | 0.005 | <0.001 |
| Ex-smokers | 0.013 | 0.005 | 0.0079 | 0.009 | 0.005 | 0.063 |
| Smokers | 0.015 | 0.005 | 0.0024 | 0.015 | 0.005 | 0.0012 |
| weighted GRS based on 97 SNPs |  |  |  |  |  |  |
| Current-Smokers | 0.012 | 0.004 | 0.0027 | 0.016 | 0.004 | <0.001 |
| Ex-smokers | 0.009 | 0.004 | 0.0116 | 0.007 | 0.004 | 0.067 |
| Smokers | 0.010 | 0.004 | 0.0030 | 0.003 | 0.003 | 0.0017 |
