# Supplementary material for: Pleiotropy of genetic variants on obesity and smoking phenotypes: Results from the Oncoarray Project of The International Lung Cancer Consortium
Source: PLoS One. 2017 Sep 28;12(9):e0185660. doi: 10.1371/journal.pone.0185660 (PMC5619832; doi:10.1371/journal.pone.0185660)
Supplement: S3 Table — (DOCX) [file pone.0185660.s003.docx]

|  | **Un-weighted GRS of 241 SNPs** | | | **Un-weighted GRS of 97 SNPs** | | | **Weighted GRS of 241 SNPs** | | | **Weighted GRS of 97 SNPs** | | |
| --- | --- | --- | --- | --- | --- | --- | --- | --- | --- | --- | --- | --- |
| **Category** | **Coef** | **95%CI** | **p-value** | **Coef** | **95%CI** | **p-value** | **Coef** | **95%CI** | **p-value** | **Coef** | **95%CI** | **p-value** |
| Total (n=12,822) | 0.022 | 0.004-0.039 | 0.014 | 0.02 | 0.009-0.043 | 0.003 | 0.021 | 0.004-0.039 | 0.016 | 0.023 | 0.006-0.041 | 0.023 |
| Stratified by smoking categories |  |  |  |  |  |  |  |  |  |  |  |  |
| Current smokers (n=6,575) | 0.024 | 0.0001-0.048 | 0.049 | 0.022 | -0.002-0.046 | 0.071 | 0.022 | -0.002-0.046 | 0.071 | 0.021 | -0.003-0.045 | 0.092 |
| Ex-smokers (n=6,245) | 0.009 | -0.016-0.034 | 0.472 | 0.020 | -0.005-0.044 | 0.120 | 0.006 | -0.019-0.031 | 0.633 | 0.014 | -0.011-0.039 | 0.271 |
| Stratified by disease status |  |  |  |  |  |  |  |  |  |  |  |  |
| Cases (n=8,078) | 0.018 | -0.004-0.040 | 0.109 | 0.026 | 0.004-0.048 | 0.019 | 0.017 | -0.003-0.046 | 0.127 | 0.024 | 0.003-0.046 | 0.029 |
| Controls (n=4,744) | 0.031 | 0.003-0.060 | 0.030 | 0.028 | 0.000-0.057 | 0.050 | 0.033 | 0.005-0.062 | 0.022 | 0.026 | -0.002-0.055 | 0.070 |

S3 Table: The comparison of partial correlations between different BMI-GRSs and pack-years of smoking in smokers
