# Supplementary material for: Pleiotropy of genetic variants on obesity and smoking phenotypes: Results from the Oncoarray Project of The International Lung Cancer Consortium
Source: PLoS One. 2017 Sep 28;12(9):e0185660. doi: 10.1371/journal.pone.0185660 (PMC5619832; doi:10.1371/journal.pone.0185660)
Supplement: S4 Table — (DOCX) [file pone.0185660.s004.docx]

S4 Table:

The comparison of associations of seven candidate pleiotropic SNPs and BMI before and after adjustment for smoking phenotypes.

| **SNP** | **chr** | **position** | **gene** | **Before adjustment** | | **After adjustment** | |
| --- | --- | --- | --- | --- | --- | --- | --- |
|  |  |  |  | **Estimate** | **P-value** | **Estimate** | **P-value** |
| **Pack-years** | | | | | | | |
| rs13021737 | 2 | 632348 |  | 0.368 | 5.00E-08 | 0.356 | 3.08E-07 |
| rs3888190 | 16 | 28889486 | ATP2A1 | 0.112 | 0.038 | 0.109 | 0.050 |
| rs11165643 | 1 | 96924097 |  | 0.168 | 0.001 | 0.161 | 0.003 |
| rs1528435 | 2 | 181550962 | AC009478.1 | 0.108 | 0.043 | 0.135 | 0.014 |
| rs11583200 | 1 | 50559820 | ELAVL4 | 0.114 | 0.0331 | 0.124 | 0.024 |
| **Smoking status** | | | | | | | |
| rs11030104 | 11 | 27684517 | BDNF | 0.131 | 0.038 | 0.128 | 0.045 |
| rs7550711 | 1 | 110082886 | GPR61 | 0.360 | 0.026 | 0.370 | 0.0255 |
| rs12016871 | 13 | 28017782 | MTIF3 | 0.195 | 0.004 | 0.186 | 0.007 |
| rs12220375 | 10 | 104901491 | NT5C2 | 0.233 | 0.010 | 0.246 | 0.007 |
| rs929641 | 2 | 58792377 | LINC01122 | 0.150 | 0.004 | 0.156 | 0.003 |
| rs6990042 | 8 | 14173974 | SGCZ | 0.084 | 0.101 | 0.111 | 0.032 |
| rs9275595 | 6 | 32681355 | XXbac-BPG254F23.7 | 0.134 | 0.031 | 0.137 | 0.029 |
